# Surfactant aggregation biases aerosol-cloud climate effects

**DOI:** 10.1039/d6cp02514e

**Published:** 2026-11-04

**Authors:** Anne Selent, Pär Håkansson, Sampo Vepsäläinen, Ilari Ainasoja, Sanna Komulainen, Pau Mayorga Delgado, Otto Mankinen, Jiří Mareš, Ritu Ghanghas, Silvia Calderón, Vladimir V. Zhivonitko, Jack J. Lin, Jussi Malila, Ville-Veikko Telkki, Nønne L. Prisle

**Affiliations:** a NMR Research Unit, University of Oulu P.O. Box 3000 FI-90014 Finland parhakansson22@gmail.com ville-veikko.telkki@oulu.fi; b NANOMO Research Unit, University of Oulu P.O. Box 3000 FI-90014 Finland; c Finnish Meteorological Institute P.O. Box 1627 FI-70211 Kuopio Finland; d Centre for Atmospheric Research, University of Oulu P.O. Box 4500 FI-90014 Finland Nonne.Prisle@oulu.fi; e Centre for Molecular Water Science CMWS, Deutsches Elektronen-Synchrotron DESY Notkestraße 85 22607 Hamburg Germany noenne.prisle@desy.de; f Institute of Inorganic and Applied Chemistry, University of Hamburg Martin-Luther-King-Platz 6 20146 Hamburg Germany

## Abstract

Organic surfactants are common constituents of atmospheric aerosols, and their internal organization has potentially important yet insufficiently understood implications for cloud droplet growth and global climate effects. Here, we demonstrate that advanced NMR relaxation and diffusion measurements, combined with state-of-the-art relaxation modeling, reveal aggregation behavior that departs markedly from conventional descriptions. Our NMR analysis shows that in aqueous sodium decanoate solutions, surfactants do not exist solely as monomers below the critical micelle concentration (CMC); instead, they form small clusters comprising a few molecules. Comprehensive modeling that incorporates dynamical and structural parameters from molecular dynamics (MD) simulations yields realistic estimates of micelle dimensions above the CMC, with diameters of approximately 5.5 nm. Water diffusion experiments further indicate the presence of small water-encapsulating aggregates at surfactant concentrations above 550 mM, and ultrafast diffusion exchange spectroscopy (UF-DEXSY) enables quantification of exchange rates between encapsulated and bulk water. Incorporating these aggregation phenomena into thermodynamic modeling of aerosol hygroscopic growth and cloud droplet activation reveals that hygroscopic properties typically measured at lower relative humidity, where aggregation is more pronounced, can significantly misrepresent aerosol cooling potential and introduce substantial biases into global climate predictions.

## Abstract 2

Advanced NMR relaxation, diffusion, and MD-assisted modeling reveal previously overlooked surfactant aggregation in aqueous sodium decanoate. These aggregates can substantially bias predictions of aerosol hygroscopicity and cloud activation.

## Graphical abstract

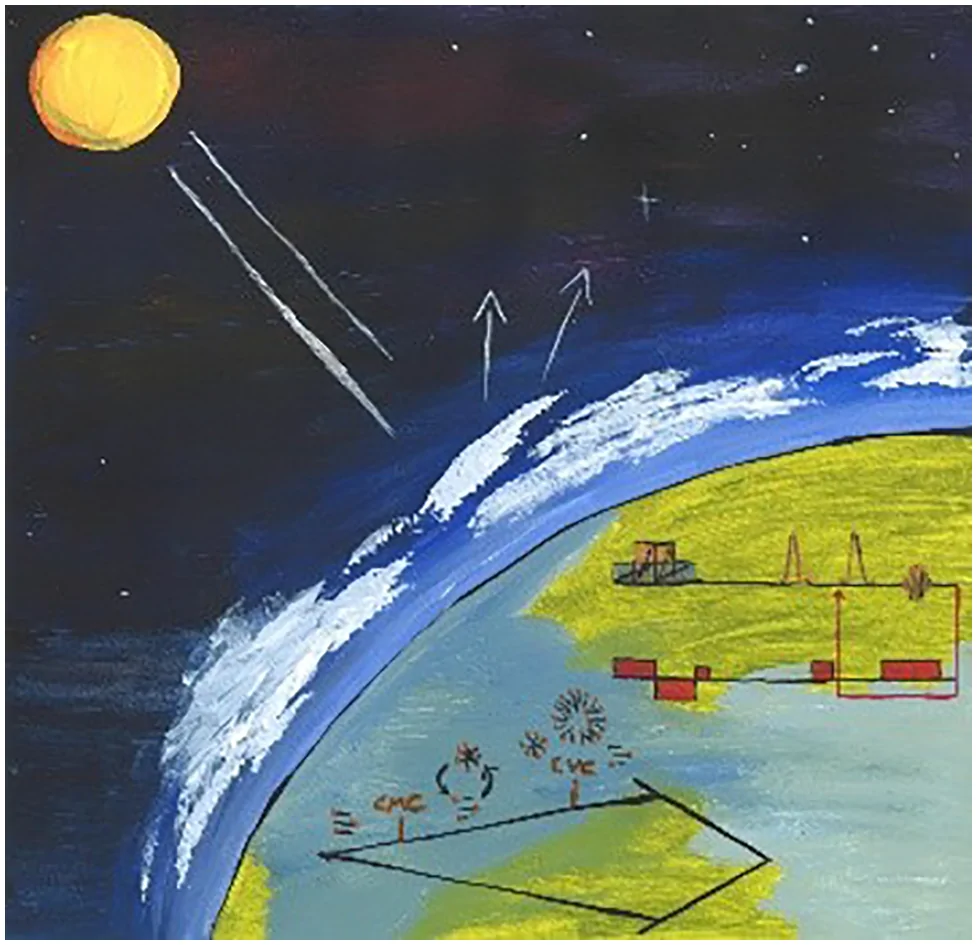

## 1 Introduction

Cloud droplets form in the atmosphere when water condenses onto the surfaces of aerosol particles. Because clouds interact with solar radiation, they can exert a substantial cooling influence on the climate. However, aerosol-cloud-climate interactions vary strongly with ambient conditions and remain among the least well-constrained components of the Earth's climate system.^1,2^ Significant progress has been made in understanding how cloud formation is influenced by partial dissolution^3^ and surface adsorption^4,5^ of particle constituents, as well as by co-condensing vapors.^6,7^

Organic surfactants are common constituents of atmospheric aerosols. The influence of their surface activity on cloud condensation nuclei (CCN) activation has been investigated extensively and involves complex interactions between effects of lowering droplet surface tension and increased water activity from concurrent bulk depletion.^7–11^ Many surfactants can also form aggregates, such as clusters and micelles, within aqueous solutions. Yet, to our knowledge, the presence of surfactant aggregates within the droplet phase has not been explicitly incorporated into predictions of cloud-forming potential. A few studies have considered micelle formation above the CMC as a mechanism that limits the amount of dissolved species in the droplet phase, analogous to finite water solubility.^8^ In most treatments, however, activated cloud droplets are assumed to be sufficiently dilute that micelles or other aggregates do not form.^8,9^ Prior to activation, during the early stages of droplet growth, aggregation may nonetheless play a significant role and substantially modify the shape of droplet growth curves.

Traditionally, surfactants are thought to exist as monomers in aqueous solution at low concentrations, whereas at higher concentrations they assemble into aggregates such as spherical or rod-like micelles, lamellar phases, and vesicles.^12^ The morphology of these aggregates depends on factors including concentration, pH, co-solutes, and the specific surfactant species.^13^ Surfactant organization in aqueous systems has been examined using a wide range of analytical techniques, including small-angle X-ray scattering (SAXS), laser and infrared spectroscopy, surface pressure measurements, scanning electron and atomic force microscopy, and neutron reflection.^13–19^

Nuclear magnetic resonance (NMR) spectroscopy is one of the most powerful tools in bulk chemical analysis and enables non-invasive probing of molecular dynamics without the need for tracers.^20,21^ It has been widely applied to the study of surfactant aggregation.^22–24^ For instance, several NMR-based approaches exist for determining the CMC, relying on changes in chemical shifts, relaxation times, or diffusion coefficients.^23,24^ However, as discussed below, some commonly used empirical models for interpreting NMR data are overly simplified, which can lead to inaccurate conclusions about surfactant aggregation behavior.

Here, we investigate the aggregation of sodium decanoate in aqueous solution using advanced NMR relaxation and diffusion measurements together with relaxation modeling. Decanoic acid – the protonated form of decanoate – is a representative fatty acid and frequently used as proxy for surface active atmospheric aerosol.^5,8,9,25–27^ Fatty acids are ubiquitous in the atmosphere, particularly in marine aerosols,^28^ which constitute a major fraction of global aerosol mass.^8^ Their occurrence in molecular or anionic form depends on aerosol pH. We use the sodium salt of decanoic acid in our experiments because of its higher solubility in water and to mimic conditions in slightly alkaline seawater. Both decanoate and decanoic acid exhibit pronounced surface activity in aqueous environments.^7,8,29^

Diffusion NMR measurements provide access to the translational self-diffusion coefficients of molecules, whereas relaxation times report on both rotational and translational motions.^30,31^ Moreover, the transverse relaxation time *T*_2_ is particularly sensitive to slow molecular dynamics compared with the longitudinal relaxation time *T*_1_.^32^ Combining diffusion, *T*_1_, and *T*_2_ measurements therefore offers a comprehensive view of the dynamics and aggregation behavior of aqueous surfactants. Accurate interpretation of these data, however, requires advanced relaxation modeling.^32,33^ In this work, we incorporate dynamical and structural parameters obtained from molecular dynamics (MD) simulations, thereby reducing the number of adjustable parameters in the relaxation models. In addition to surfactant diffusion, we quantify water diffusion, as the presence of multiple water diffusion coefficients can indicate encapsulation phenomena. Finally, ultrafast diffusion exchange spectroscopy (UF-DEXSY)^34^ enables direct quantification of exchange between encapsulated and bulk water. We further apply sophisticated thermodynamic frameworks^35,36^ together with Köhler theory^37^ to assess how aggregation influences cloud droplet growth.^38^

## 2 Results and discussion

### 2.1 Surfactant aggregation revealed by NMR

The ^1^H NMR spectrum of 704 mM sodium decanoate in water (1% H_2_O, 99% D_2_O; see Materials and methods in SI), shown in Fig. 1A, displays four resonances from the decanoate anion and one from water. The spectral lines remain relatively narrow across the entire concentration range, indicating that the ^1^H nuclei experience effectively isotropic motion on the NMR timescale as a result of molecular and micellar rotation, as well as molecular exchange processes.^32^ The *T*_1_ and *T*_2_ relaxation times of the decanoate methylene ^1^H signal P3 as a function of concentration are presented in Fig. 1B. Signal P3 was selected as the primary reporter throughout this study because it is the most intense resonance and originates from the hydrophobic region within the molecular aggregates. The relaxation times of the remaining signals are presented in Fig. S1. The *T*_1_ values remain nearly constant below 100 mM and then gradually decrease at higher concentrations. Likewise, the decanoate diffusion coefficient *D* (Fig. 1C) begins to decline above 100 mM. Together, these trends indicate a critical micelle concentration (CMC) of approximately 100 mM for sodium decanoate,^24^ consistent with CMCs reported in the literature (50–120 mM; the range may be broad due to the pre-aggregation phenomena discussed below).^38–41^

**Fig. 1:**
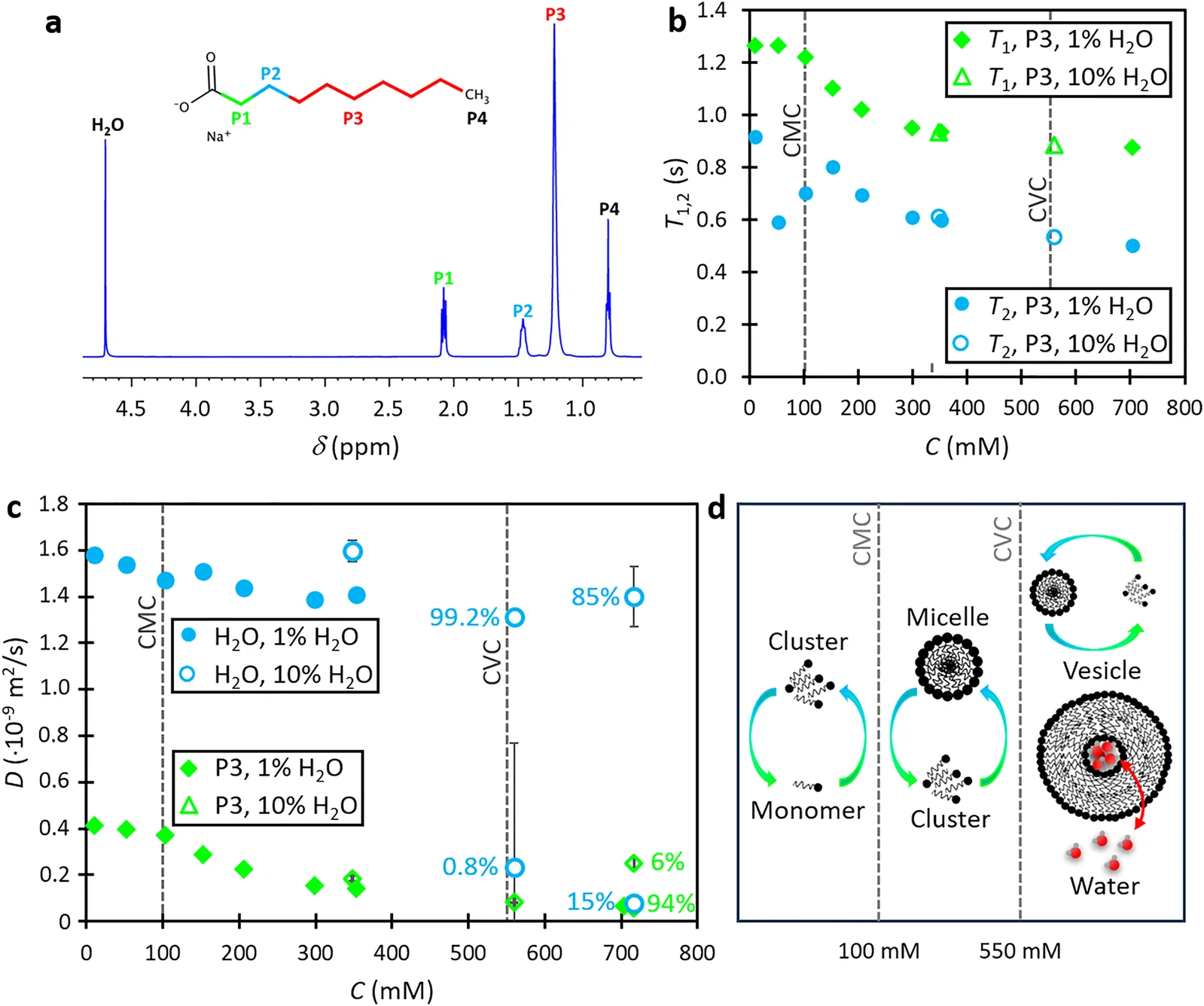
Dynamics and aggregation of sodium decanoate in aqueous solution. (A) ^1^H NMR spectrum of 704 mM sodium decanoate in water (1% H_2_O, 99% D_2_O) acquired at 500 MHz. (B) *T*_1_ and *T*_2_ relaxation times of the methylene resonance P3 as a function of sodium decanoate concentration. (C) Diffusion coefficients of decanoate (green) and water (blue) as a function of concentration. Water diffusion coefficients are higher in samples with higher H_2_O concentrations because they contain a smaller fraction of heavy water.^42^ (D) Schematic illustrations of the aggregate structures characteristic of each concentration regime. Critical micelle and vesicle concentrations (CMC and CVC) are marked by gray vertical dashed lines.

According to the conventional interpretation,^24,43^ surfactants exist as monomers in solution below the CMC. Above the CMC, micelles form and coexist with monomers; the monomer concentration remains fixed at the CMC, while the micelle concentration increases with total surfactant concentration, and the micelle size is assumed to remain constant. Because *T*_1_ and *D* values of micelles are lower than those of monomers, and because exchange between the two environments is fast on the NMR timescale, the observed *T*_1_ and *D* represent population-weighted averages of the monomeric and micellar states. Consequently, at higher concentrations, the measured *T*_1_ and *D* values gradually approach those characteristic of micelles.^23^ The conventional two-site exchange model reproduces the trends in the *T*_1_ and *D* data (Fig. S2). However, the micellar diffusion coefficient obtained from this model, *D*_mic_ = (2 ± 1) × 10^−12^ m^2^ s^−1^, corresponds – *via* the Stokes–Einstein equation (SI eqn (S8))^44^ – to an apparent micelle diameter of 200 ± 100 nm. Given that micelle sizes are typically on the order of twice the surfactant molecular length, and that a decanoate molecule is approximately 1.2 nm long, the obtained size is physically unrealistic, indicating that the conventional analysis is not valid for this system.

Below the CMC, the *T*_2_ values of decanoate (Fig. 1B) are markedly shorter than the corresponding *T*_1_ values. If the surfactants existed solely as monomers in solution, they would be expected to fall within the fast-motion (extreme narrowing) regime, where *T*_1_ and *T*_2_ are equal.^32^ However, the markedly shorter *T*_2_ values observed indicate that, contrary to the conventional model, the surfactants experience restricted motion arising from intermolecular association, suggesting the presence of aggregates or clusters even below the CMC. To investigate this phenomenon, we performed detailed relaxation modeling (see Section S2.1 of SI).^45^ The modelling assumes that intramolecular dipolar relaxation is the dominant relaxation mechanism and that all other contributions are negligible, an approximation that is generally well justified for isotropic surfactant systems.^22^ Under this assumption, the relaxation behaviour can be satisfactorily described within the secular approximation,^22^ using only two parameters, *T*_1_ and *T*_2_. The experimentally observed relaxation curves are well fitted by a single-exponential model, further supporting the validity of this two-parameter description. Multiple intramolecular dipolar interactions were taken into account by computing the explicit time-dependent dipole–dipole distances and corresponding time-correlation functions as a starting point of the relaxation model.

We simulated the dynamics of clusters containing one to five surfactant anions together with Na^+^ counterions (Fig. S4). These simulations yielded the time-correlation functions and translational diffusion coefficients required for the intracluster/molecular dipolar ^1^H NMR relaxation model. Incorporating these MD-derived parameters allowed us to explicitly account for surfactant molecular dynamics and to minimize the number of adjustable parameters compared with conventional approaches.^23^ We assumed fast exchange between coexisting monomeric and multimeric species. We note that exchange associated with the acid–base equilibrium is unlikely to play a major role in relaxation, because the exchanging proton does not directly contribute to the P3 signal, and any indirect effects are expected to be minimal because of the large distance between the exchanging proton and the P3 protons; furthermore, the fraction of protonated decanoic acid is expected to be small under the experimental conditions (pH 7.0–8.5). The best agreement with the experimental relaxation and diffusion data was obtained for a model comprising monomers and pentamers, with monomer fractions of 23% at 10 mM and 0% at 50 and 103 mM. These results provide strong evidence that clusters of surfactant anions exist below the CMC, in contrast to the traditional interpretation. In practice, the system likely contains a distribution of cluster sizes. Notably, sub-CMC (premicellar) aggregation has also been proposed in several earlier studies.^46–48^ For example, using diffusion measurements with radioactive labels and phenomenological modeling, Lindman and Brun^49^ reported substantial premicellar aggregation well below the CMC in aqueous sodium octanoate solutions, consistent with our findings.

The diffusion and relaxation data above the CMC were analyzed using a model that assumes two surfactant populations: larger micelles of fixed size and smaller clusters, with fast exchange between the two environments. The concentration of the cluster population was fixed to the CMC. The conventional model can be viewed as a simplified version of this framework, in which the second population consists of monomers rather than clusters.^23,24^ As the micelle concentration increases, mutual hindrance leads to reduced micellar mobility, resulting in an approximately 20% decrease in the micellar diffusion coefficient at the highest concentration (704 mM).^50,51^ Cluster diffusion is also slightly hindered (by about 6% at the highest concentration). This obstruction effect was explicitly included in our model.^50,51^ The model incorporates information from MD simulations of a 66-surfactant micelle (Fig. S5), which provided the fast intramolecular dipolar relaxation dynamics and the residual order parameter. To avoid overestimating relaxation rates, an additional slow molecular process, molecular protrusion, was included.^52,53^ In this process, a surfactant molecule transiently protrudes from the micelle before returning (illustrated in Fig. S13). The resulting model reproduces the experimental data well (Fig. S14). By integrating MD-derived dynamical and structural parameters and accounting for clustering, obstruction, and protrusion effects, our relaxation modeling yields a micelle diameter of 5.4 nm. This value is one to two orders of magnitude smaller than the estimate obtained from the conventional model (200 ± 100 nm), underscoring the importance of avoiding oversimplified models when interpreting NMR data of surfactant solutions. The resulting size corresponds to 48 decanoate anions, which is comparable to the aggregation number of 30 reported for aqueous sodium octanoate solutions above the CMC.^54^

Diffusion coefficients of water in aqueous sodium decanoate solutions are also shown in Fig. 1C. Above 550 mM, two distinct water diffusion coefficients appear: one at approximately 1.4 × 10^−9^ m^2^ s^−1^ (relative population 85% at 717 mM), corresponding to free water, and another at 8.0 × 10^−11^ m^2^ s^−1^ (15%), which matches the diffusion coefficient of decanoate anions. This strongly indicates that some decanoate anions encapsulate a fraction of the water. It is known that unilamellar vesicles can form in aqueous sodium decanoate solutions,^38^ and we expect these vesicles to encapsulate a fraction of the surrounding water. From this behavior, we infer a critical vesicle concentration (CVC) of about 550 mM. Standard optical techniques, such as nanoparticle tracking analysis,^55^ may not detect vesicles of this size, but our results indicate that they become indirectly observable through multicomponent water diffusion. The emergence of vesicles may also explain why the relaxation model overestimates the diffusion coefficient at the highest concentration, as the model assumes micelles rather than larger vesicular structures. Notably, at 704 mM, the diffusion coefficient of decanoate itself splits into two components – *D* = 2.5 × 10^−10^ m^2^ s^−1^ (6%) and 8.0 × 10^−11^ m^2^ s^−1^ (94%) – suggesting the coexistence of micelles and vesicles, with vesicles being more abundant. Ultrafast diffusion exchange spectroscopy (UF-DEXSY) (Fig. 2) further reveals that the lifetimes of water molecules in the free and encapsulated pools are approximately 140 ± 30 and 48 ± 15 ms, respectively.^34^

**Fig. 2:**
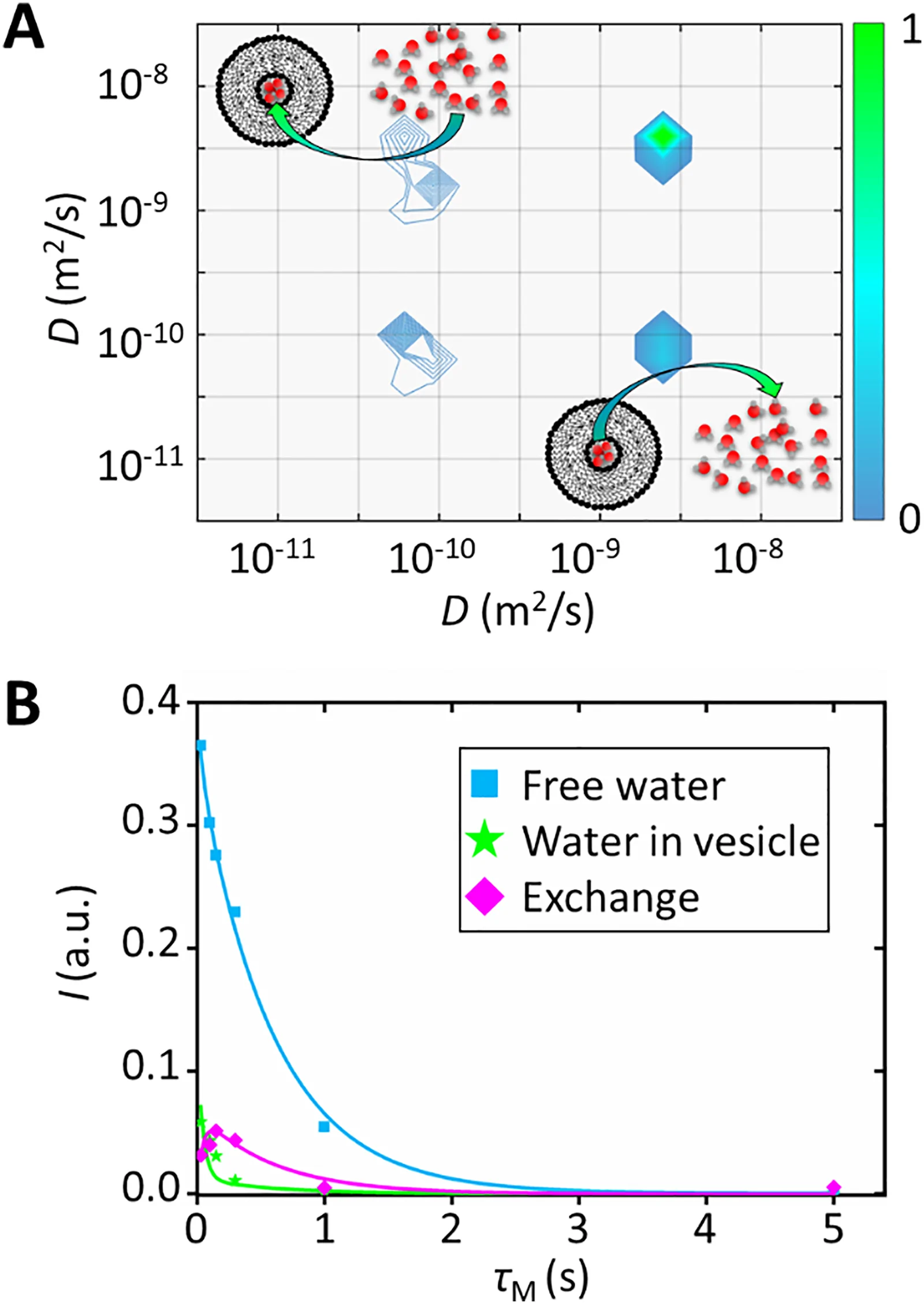
UF-DEXSY analysis of exchange between vesicle-encapsulated and free water. (A) Water diffusion-exchange map of a solution containing 717 mM sodium decanoate in water (10% H_2_O, 90% D_2_O), acquired with a mixing time of 100 ms. The diffusion coefficients of free water and vesicle-encapsulated water are approximately 2 × 10^−9^ and 8 × 10^−11^ m^2^ s^−1^, respectively. Off-diagonal signals indicate exchange of water molecules between the two environments. (B) Peak integrals from the diffusion-exchange maps plotted as a function of mixing time *τ*_M_, together with fits of a two-site exchange model (solid lines). The fit yields an exchange rate of *k* = 28 ± 6 s^−1^.

The decanoate aggregate structures identified in this NMR study are summarized in Fig. 1C. Below the CMC, we observe small clusters composed of a few surfactant anions, rather than monomers as assumed in the conventional model. The decreases in *T*_1_ relaxation time and in the decanoate diffusion coefficient indicate a CMC of approximately 100 mM, a value that remains essentially unchanged across the pH range 7.5–12 (SI Appendix Fig. S17). Above the CMC, micelles and small clusters – rather than monomers – coexist in solution, and the micelles have a diameter of about 5.4 nm (48 decanoate anions). Two distinct water diffusion coefficients appear above 550 mM, indicating the formation of aggregates that encapsulate water, most likely vesicles. UF-DEXSY analysis shows that the exchange rate between vesicle-encapsulated and free water is approximately 28 s^−1^. The presence of two surfactant diffusion coefficients further suggests that, above the CVC, vesicles coexist with micelles and small clusters, with vesicles being the dominant species. Overall, this NMR study demonstrates that integrating experimental diffusion measurements with *T*_1_ and *T*_2_ relaxation data, together with advanced relaxation modeling that incorporates dynamical and structural parameters from MD simulations, yields far more detailed and reliable insight into surfactant aggregation than phenomenological models based on a single NMR observable. In addition, advanced multidimensional relaxation and diffusion techniques – such as UF-DEXSY – provide access to aggregation and exchange phenomena that are difficult to detect by any other method.

#### 2.1.1 Influence of surfactant aggregation on cloud droplet growth

We calculated the equilibrium growth of cloud droplets formed on initially dry, spherical sodium decanoate (NaDec) particles using a thermodynamic framework based on Köhler theory.^37^ The Köhler growth curve (SI eqn (S20)) describes the equilibrium water vapor saturation ratio *S* as a function of droplet diameter *d* and arises from two competing effects: dissolved solutes lower the activity and vapor pressure of water (Raoult effect), while the positive curvature of the droplet surface increases the vapor pressure (Kelvin effect).^8^ The maximum of the Köhler curve defines the critical ambient water saturation *S*_c_ and corresponding critical droplet diameter *d*_c_ required for cloud droplet activation. Bulk-surface partitioning and the resulting droplet surface tension *σ*, driven by the surface activity of decanoate anions, were calculated using the molecular monolayer (ML) surface model of Malila and Prisle^35^ for all droplet sizes along the Köhler growth curve.^4,5^

The effect of sodium decanoate remaining in the bulk aqueous phase on droplet water activity was estimated using several approaches that account for the possible presence of clusters and micelles (Fig. 3A). The comparison of these allows us to assess the impacts of specific aggregation phenomena on droplet growth. As a first approximation, the water activity was expressed as the corrected bulk-phase water mole fraction, *a*_w_ = *x*^B^_w_, under four different assumptions:

**Fig. 3:**
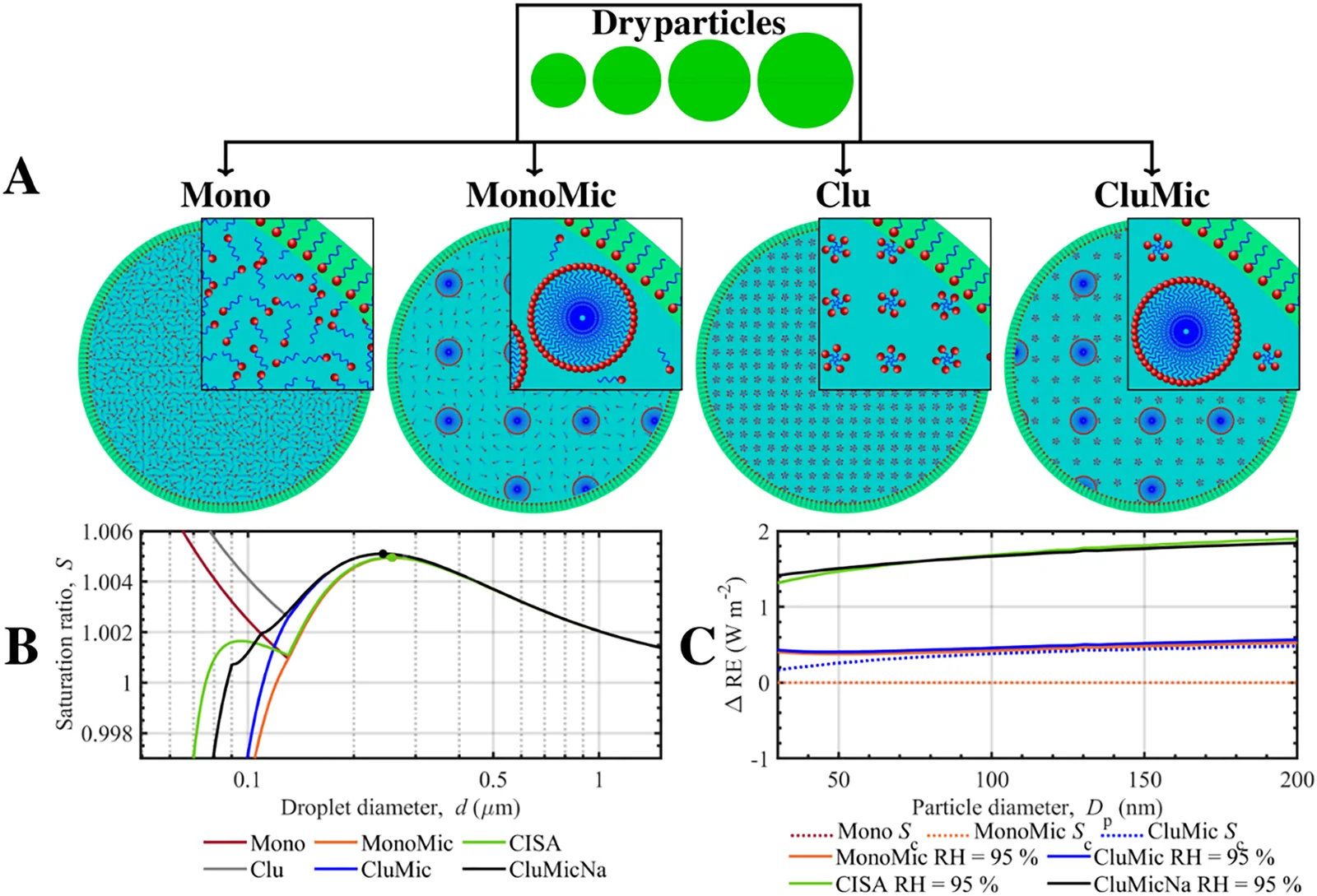
Cloud droplet growth modelling and estimated climate effects. (A) Conceptual illustration of the different water mole-fraction models used in addition to the full thermodynamic CISA framework. These water-activity models are each applied to predict the hygroscopic growth of initially dry, spherical, pure sodium decanoate (NaDec) particles with dry diameters *D*_p_ = 50, 100, 150, and 200 nm. Bulk–surface partitioning of NaDec monomers is calculated using the molecular monolayer model of Malila and Prisle.^35^ The remaining NaDec in the droplet bulk phase is treated as: 1. Mono: dissolved monomers with a maximum concentration equal to the CMC; 2. MonoMic: micelles containing *n*_m_ = 48 Dec^−^ anions coexisting with dissolved NaDec monomers; 3. Clu: clusters containing *n*_c_ = 5 Dec^−^ anions and dissolved Na^+^ counterions, with a maximum concentration equal to the CMC; 4. CluMic: clusters below the CMC and coexisting clusters and micelles above the CMC. (B) Köhler growth curves for sodium decanoate particles with *D*_p_ = 50 nm, calculated using SI eqn (S20) with the four water-activity models listed above, as well as with the counterion-binding formulation (CluMicNa) and the full thermodynamic CISA model. Corresponding curves for all particle sizes are provided in SI Fig. S19. (C) Estimated change in aerosol–cloud–climate radiative effect (ΔRE) when accounting for the formation of clusters and micelles in droplets, relative to a reference case without aggregates. ΔRE is shown as a function of the smallest activating particle diameter *D*_p_. Calculations were performed by extrapolating hygroscopicity parameters (*κ*) derived from Köhler predictions using the different water-activity models, evaluated at the critical point (*S*_c_) and under subsaturated conditions (RH = 95%).

1. *Mono model*: droplets contain only dissolved sodium decanoate monomers (NaDec → Na^+^ + Dec^−^), with the monomer concentration capped at the CMC of sodium decanoate (SI Appendix eqn (S23)).^8,9,35^

2. *MonoMic model*: below the CMC, droplets contain only dissolved monomers; above the CMC, monomers at the CMC concentration coexist with micelles composed of *n*_m_ = 48 decanoate anions (SI Appendix eqn (S24)).

3. *Clu model*: droplets contain only clusters consisting of *n*_c_ = 5 decanoate anions together with dissolved sodium counterions, with the total cluster concentration limited by the CMC (SI Appendix eqn (S25)).

4. *CluMic model*: below the CMC, droplets contain only clusters and dissolved Na^+^ counterions; above the CMC, clusters and Na^+^ ions coexist with micelles (eqn (S26)).

A conceptual illustration of these different assumptions regarding dissolution state and aggregate formation within the droplet is provided in Fig. 3A.

Electrostatic association with decanoate anionic clusters or micelles can substantially reduce the amount of free Na^+^ counterions in the bulk solution.^56^^23^Na NMR relaxation measurements, together with quadrupolar relaxation modeling, show that the fraction of free sodium ions *f*_Na^+^_ decreases markedly below unity as the sodium decanoate concentration increases (Table S8 and Fig. S19).^57^ This effect was incorporated into the CluMic model by scaling the contribution of sodium ions to the water activity according to *f*_Na^+^_, resulting in the CluMicNa formulation (SI eqn (S27)).

Finally, we applied the full thermodynamic CMC-based ionic surfactant activity (CISA) model^36^ to evaluate the water activity at all stages of growing aqueous sodium decanoate droplets. The CISA framework accounts for non-ideal solution effects arising from both the dissociation and micellization of sodium decanoate, as well as the binding of sodium counterions to micelles. In our calculations, micelles containing *n*_m_ = 48 Dec^−^ anions were assumed to coexist with dissolved NaDec monomers, and a constant fraction of 0.68 sodium counterions was taken to be bound to each micelle.

Köhler growth curves for sodium decanoate particles with a dry diameter *D*_p_ = 50 nm, calculated using the different models, are shown in Fig. 3B (with additional curves and corresponding water activities provided in Fig. S19). Differences between the models are most pronounced during the early stages of droplet growth, where solution states are more concentrated and aggregation phenomena prominent.^4,9^ The trends in Fig. 3B are consistent across all particle sizes examined in Fig. S19, which span the range typically relevant for atmospheric cloud formation. The critical activation point (*d*_c_,*S*_c_) is marked on each curve, and the properties of the activating droplets are summarized in Table S11.

Formation of clusters or micelles does not significantly influence the droplet activation threshold *S*_c_, because activation occurs at droplet solution states that remain sufficiently dilute to stay below the CMC.^8,9^ However, prior to activation – during the early stages of droplet growth – accounting for clusters, micelles, and counterion binding substantially alters the shape of the Köhler growth curves. For a given dry particle size and droplet size, the bulk-surface partitioning and surface tension are identical across all models. Thus, the differences between the Köhler curves arise solely from the effects of different considerations regarding aggregates and counterion binding on the droplet water activity.

At a given ambient water saturation *S*, the equilibrium droplet size *d* follows the order CISA < CluMicNa < CluMic < MonoMic. The presence of clusters and micelles substantially reduces the water activity (SI Fig. S19), primarily because aggregate formation allows a larger amount of dissolved sodium counterions to remain in solution above the CMC. When counterion binding is included, the predicted water activity increases slightly, reflecting a weaker Raoult effect. The full thermodynamic CISA model yields higher water activities than the CluMicNa formulation, indicating that part of the reduction in water activity is further mitigated by unfavorable non-ideal interactions between water and micelles.

Hygroscopic growth factors (HGF, SI eqn (S45)) for different ambient relative humidity (RH) conditions were calculated from the outputs of SI eqn (S20) (Fig. S20A). At all RHs, HGF increases with dry particle diameter *D*_p_, reflecting the diminishing influence of bulk-surface partitioning on the sodium decanoate bulk concentration and on the droplet surface tension *σ* in larger droplets.^8,10^ At RH = 99%, the predicted HGF at a given *D*_p_ follows the order CISA < CluMicNa < CluMic < MonoMic. The contrast between the CISA and CluMicNa predictions *versus* those from CluMic and MonoMic clearly highlights the role of counterion binding to micelles in reducing the amount of water taken up by the particles.

The hygroscopicity factor *κ* is widely used in large-scale atmospheric models as a single-value parameter that captures the influence of aerosol chemical composition on water uptake and cloud droplet formation.^58^ These *κ* values are typically derived either from cloud condensation nuclei (CCN) activation measurements at the critical point (supersaturated conditions) or from hygroscopic growth factors measured under subsaturated conditions (RH < 100%). We obtained *κ* parameters for sodium decanoate particles at various ambient humidity levels along the Köhler curves calculated with the different activity models (SI Figs. S20B and S21). From these, we assessed how using *κ* values corresponding to different sub- and supersaturated conditions influences the predicted critical saturation *S*_c_ and the resulting aerosol-cloud radiative effect, when aggregation phenomena are included (SI Fig. S20C). Overall, the obtained *κ* values indicate that sodium decanoate particles exhibit moderate water uptake ability, consistent with other studies of organic aerosols.^59,60^*κ* increases systematically with dry particle size, and all models predict smaller *κ* values under subsaturated conditions than under supersaturated conditions. This behavior reflects the increasing solute fraction in the bulk phase (SI Fig. S24B) as droplets grow and become more dilute, gradually replacing micelles with clusters or monomeric decanoate anions. Under subsaturated conditions, *κ* generally decreases with increasing RH between 95% and 99%, driven by higher solute concentrations arising from the interplay between bulk–surface partitioning and micelle formation in growing droplets. Across most particle sizes and humidity conditions, *κ* values follow the order CISA < CluMicNa < CluMic < MonoMic, consistent with the trends observed for droplet water activity and hygroscopic growth factors.

The response in cloud radiative effect ΔRE to the formation of clusters, micelles, and potential counterion binding in the droplet phase is complex, depending on both the initial dry particle size and the humidity at which particle hygroscopicity is obtained. All estimated changes ΔRE based on properties at the critical point are small and closely aligned (|ΔRE| < 0.5 W m^−2^, Fig. 3C and S20C), reflecting the absence of micelles in droplets at activation. In contrast, using *κ* values obtained under subsaturated conditions introduces a strong warming bias – *i.e.*, a significantly weaker aerosol cooling effect – for all models (|ΔRE| = 0.4–2 W m^−2^) relative to the Mono model (base case). Accounting for micelle formation together with counterion binding produces a substantially stronger warming bias than models that neglect counterion binding. These results demonstrate that, for particles containing components prone to forming aggregates such as clusters and micelles, using *κ* values derived from lower-humidity measurements, where aggregates are more likely to exist, can severely misrepresent aerosol cooling potential in global climate predictions. This issue is particularly important when the aggregating monomers are ionic, as their degree of ionization depends on aerosol acidity and thus on ambient environmental conditions. The present results further indicate that this warming bias becomes even larger when *κ* values are taken from high subsaturated humidity (99%; Fig. S20C). Consequently, *κ* values obtained from ultra-high-humidity laboratory experiments^61^ or ambient measurements may surprisingly not provide more accurate representations of cloud droplets at the point of activation than those derived under more accessible lower humidity conditions.

## Author contributions

A. S.: sample preparation, NMR experiments, analysis of experimental data, writing the first version of the experimental part of the manuscript. P. H.: corresponding author, planning and performing the relaxation modelling with MD simulations, writing the first version of the relaxation modelling part of the manuscript. S. V. atmospheric modeling, related visualizations and descriptions, writing first draft of the atmospheric modeling results. I. A.: sample preparation and NMR experiments. S. K.: planning the sample preparation, sample preparation and NMR experiments. P. M. D.: long MD for computing residence times, and contributions to relaxation modelling. O. M.: carrying out and analyzing of the UF-DEXSY experiments. Jiri Mares: contributions to MD simulations, relaxation modelling, some of the NMR measurements and their analysis. R. G.: Sample preparation and NMR experiments. S. C. contribution to atmospheric modeling and first draft of related descriptions (CISA model). V. V. Z.: planning and supervision of experiments (especially the UF-DEXSY experiments). J. J. L.: contribution to analysis of experimental data and planning of relaxation modelling. J. M.: contribution to analysis of experimental data, planning of relaxation modelling, and atmospheric modeling (climate forcing estimation). V.-V. T.: corresponding author, ideation, planning the study, writing the full version of the manuscript combining the experimental and modelling NMR data. N. L. P.: corresponding author, ideation, planning the study, contribution to analysis of experimental data and planning of relaxation modelling, planning atmospheric modeling and related visualizations, writing sections concerning atmospheric modelling for the full version of the manuscript. All the authors contributed to the final version of the manuscript.

## Conflicts of interest

There are no conflicts to declare.

## Supplementary Material

CP-OLF-D6CP02514E-s001

## Data Availability

Materials and methods as well as supporting figures and tables are available in the supplementary information (SI). Supplementary information is available. See DOI: https://doi.org/10.1039/d6cp02514e.
