# Supplementary material for: Surfactant aggregation biases aerosol-cloud climate effects
Source: Phys Chem Chem Phys. 2026 Nov 4. Online ahead of print. doi: 10.1039/d6cp02514e (PMC13750523; doi:10.1039/d6cp02514e)
Supplement: Supplementary file 1 [file CP-OLF-D6CP02514E-s001.pdf]

Supplementary Information (SI) for Physical Chemistry Chemical Physics.
This journal is © the Owner Societies

Supporting Information for

Surfactant aggregation may bias aerosol cooling potential

Anne Selent[1], Pär Håkansson[1*], Sampo Vepsäläinen[2], Ilari Ainasoja[1], Sanna Komulainen[1], Pau Mayorga Delgado[1], Otto Mankinen[1], Jiří Mareš[1], Ritu Ghanghas[1], Silvia Calderón[2,3], Vladimir V. Zhivonitko[1], Jack J. Lin[2,4], Jussi Malila[2], Ville-Veikko Telkki[1*] & Nønne L. Prisle[4,5,6*]

[1]NMR Research Unit, University of Oulu; P.O. Box 3000, FI-90014, Finland

[2]NANOMO Research Unit, University of Oulu; P.O. Box 3000, FI-90014, Finland

[3]Finnish Meteorological Institute; P.O. Box 1627, FI-70211, Kuopio, Finland

[4]Centre for Atmospheric Research, University of Oulu; P.O. Box 4500, FI-90014, Finland

[5]Centre for Molecular Water Science CMWS, Deutsches Elektronen-Synchrotron DESY, Notkestraße 85, 22607 Hamburg, Germany; email: noenne.prisle@desy.de

[6]Institute of Inorganic and Applied Chemistry, University of Hamburg, Martin-Luther-King-Platz 6, 20146 Hamburg, Germany

***Corresponding authors:**

Pär Håkansson, Ph.D. (e-mail: parhakansson22@gmail.com)

Ville-Veikko Telkki, Prof. (e-mail: ville-veikko.telkki@oulu.fi)

Nønne L. Prisle, Prof. (e-mail: Nonne.Prisle@oulu.fi)

Contents

S1 Materials and methods 3
S1.1 Experimental NMR 3
S1.2 NMR relaxation modeling 5
S1.2.1 Atomistic molecular simulation 6
S1.2.2 Transition from MD simulation to NMR relaxation 8
S1.2.3 Small cluster MD results 10
S1.2.4 Micelle MD results 12
S1.2.5 Parameter estimation from the relaxation and diffusion data 13
S1.2.6 Interpretation of low concentration relaxation/diffusion experiments 14
S1.2.7 Interpretation of relaxation/diffusion experiments above the CMC 16
S1.2.8 Model comparison 19
S1.3 Atmospheric modeling 21
S1.3.1 Köhler theory 21
S1.3.2 Bulk-surface partitioning model 22
S1.3.3 Water activity models 23
S1.3.4 Compound and solution properties 27
S1.3.5 Particle hygroscopicity 30
S1.3.6 Cloud droplet number concentration and climate radiative effect 32
S2 Supplementary figures 34
S3 Supplementary tables 60
References 72

S1 Materials and methods

S1.1 Experimental NMR

Sodium decanoate (98%) was purchased from Sigma-Aldrich. The concentration of sodium decanoate in aqueous samples varied between 10 and 717 mM. The water solvent included either 1% or 10% of deionized H_2O in D_2O (99% D, Sigma-Aldrich). The higher H_2O concentrations were used above the CVC for higher signal-to-noise ratio to better detect the two water diffusion coefficients. Mimicking sea spray aerosols, the effect of inorganic co-solute was studied by adding either 0.1 or 0.5 M NaCl to the sodium decanoate solution (see Fig. S16). In the beginning of the sample preparation process, sodium decanoate was added to an Eppendorf tube. Then water was added, and the sample was mixed until all the sodium decanoate was dissolved. Then a tiny drop of DMSO (DMSO D6 +0.003%TMS, water < 0.02%, 99.80%D, Euriso-top) was added for locking and referencing purposes and the sample was mixed again. The pH of the sample was measured with pH paper, and it increased from 7.0 to 8.5, when the concentration of sodium decanoate increased from 10 to 704 mM. Then the mixed solution was moved into the 5 mm NMR tube, and the tube was sealed with a cap, Teflon tape and parafilm.

^{1}H NMR relaxation and diffusion measurements were performed on a Bruker Avance III 500 MHz spectrometer while the ^{1}H UF-DEXSY experiments were carried out on a Bruker Avance III 400 MHz spectrometer. All measurements were conducted at room temperature (296 K) and at ambient pressure. T_1 relaxation times were measured using the inversion recovery pulse sequence. [1] The recovery time varied logarithmically from 0.001 to 25 s, and the number of steps was 16. The number of scans was 8, the relaxation delay was 5 s and the experiment time was 34 min. T_2 relaxation times were measured using the Carr–Purcell–Meiboom–Gill (CPMG) sequence. [2] The echo time was 0.49 ms, the number of echoes varied from 2 to 8000, and the number of steps was 16. The number of scans was 8, the relaxation delay was 5 s and the experiment time was 19 min. Pulsed-field-gradient stimulated echo (PGSTE) sequence [3] with bipolar gradients [4] was used in the diffusion measurements

(Bruker stebpgp1s sequence). The gradient strength varied from 2% to 98% of the maximum gradient value of 52.43 G/cm, and the number of steps was 16. The diffusion delay Δ (70 – 190 ms) and the length of the gradient pulse δ (3 – 5 ms) was optimized for each sample to obtain sufficient decrease for the echo signal. Number of scans and dummy scans were 8 and 4, respectively, relaxation delay 5 s and the experiment time 20-30 min. In the UF-DEXSY experiments, the number of accumulated scans was 64, the relaxation delay was 60 s and the experiment time was 1 h 5 min. Altogether 6 experiments were performed with the mixing time τ_M varying from 30 ms to 5 s. The other details of the UF-DEXSY experiments are reported in Ref. [5]

Additionally, the effect of pH on CMC was tested. Aqueous solutions with 50, 100, 300 and 700 mM concentration of sodium decanoate were prepared for this purpose. These solutions were divided into several samples. The pH of the samples was either decreased or increased using deuterated HCl and NaOH solutions (Merck, DCl 35 wt. % in D_2O, ≥ 99 atom % D, NaOD 40 wt. % in D2O, 99.5 atom % D). The solutions were further diluted to 1% and 10%, respectively, for easier adjustment of pH. The sodium decanoate itself acted as a buffer, as the pH change occurred very slowly at first and the high concentration samples (500mM and 700 mM) required significantly stronger acid and base solutions. The 50 and 100 mM samples included 0.5 ml of a 90% D_2O and the 300, 500 and 700 mM samples contained 0.8 ml of 97% D_2O. A maximum of 20 µl of acid or base solution was used during the process. Therefore, the concentration of the sodium decanoate was not significantly affected by the pH adjustment. However, as the samples showed clear visual changes when the pH was lowered by more than 1 pH step from the original, only samples with pH 7.5 or higher were taken into account in this analysis. The pH of the samples was measured with a Toledo FiveEasy™ F20.

^{1}H NMR relaxation times and diffusion coefficients of the variable pH samples were measured with a high-field Bruker Avance III 400 MHz spectrometer using 5mm BBO. All measurements were conducted at room temperature (296 K) and at ambient pressure. T_1 relaxation

times were measured using the saturation recovery pulse sequence. The recovery time varied logarithmically from 0.1 to 50 s, and the number of steps was 16. The number of scans was 16, the relaxation delay was 30 s and the experiment time was 3 h 51 min. T_2 relaxation times were measured using the CPMG sequence. The echo time was 1 ms, the number of echoes varied from 2 to 32000, and the number of steps was 16. The number of scans was 16 or 32, the relaxation delay was 30 or 40 s and the experiment time was 2 h 44 min or 6 h 40 min, respectively. PGSTE sequence [3] with bipolar gradients [4] was used in the diffusion measurements (Bruker stebpgp1s sequence). The gradient strength was varied from 2% to 98% of the maximum gradient value of 48.2 G/cm, and the number of steps was 16. The diffusion delay Δ (100 – 200 ms) and the length of the gradient pulse δ (2 – 4 ms) was optimized for each sample to obtain sufficient decrease for the echo signal. Number of scans was 32 or 6, relaxation delay was between 30 and 40 s and the experiment time varied between 4 h 50 min and 9 h 40 min.

S1.2 NMR relaxation modeling

The relaxation modelling concerned the data of the relaxation and diffusion experiments of the samples including sodium decanoate in 1% H_2O and 99% D_2O solution. The translational self-diffusion and experimental relaxation rates (R_1=1/T_1, R_2=1/T_2) as a function of the inverse of total surfactant concentration (C^{-1}) for the four decanoate ^{1}H signals (P1-P4) are shown in Fig. S3. At low C^{-1} (high concentration), R_2 and D depends approximately linearly on C^{-1}. The linear behavior ends around C^{-1} = 0.01 mM^{-1}, enabling the identification of CMC at 100 mM, similarly to previous diffusion [6,7] and relaxation [8] studies.

Relaxation modeling provides further insights on the water-surfactant system. The modelling study has the two parts: the first one is addressing plausible models below the CMC and the second above the CMC. MD simulations were conducted, and information was merged in

analytical models to reduce the number of parameters or constrain them, as well as to parameterize the size of aggregates.

S1.2.1 Atomistic molecular simulation

MD trajectories were computed with the AMBER program package [9] version 2016 in order to extract parameters accounting for dynamics and static properties and thus reducing the number of adjustable parameters.

For modeling surfactant clusters below CMC, a quantum mechanics/molecular mechanics (QM/MM) method was used to separately simulate monomers as well as dimer to pentamer clusters. In these cases, the set of surfactants was treated with the QM method at every time step, providing the forces that enter the simulations, whereas the solvent water molecules and sodium ions were described classically. To provide a model input for higher surfactant concentrations, three micelles with 11, 25, 66 surfactants were simulated with the classical MM, with details outlined below. Motivation for the use of either the QM or MM are further discussed below, but, briefly, the important weak interactions for few surfactants in close proximity benefit from the QM representation, whereas computational cost makes the classical MM relevant for the larger aggregates.

Common for all simulations is the flexible single point charge water force field (SPC/Fw), [10] and the steps conducted to reach an equilibrated temperature at 293 K. The simulation timespan and the number of atoms of the simulated systems are provided in Table S1. The setup of each system involves the initial energy minimization of the complete system (water, surfactants and sodium ions) consisting of 6000 steps, with alternating steepest descent and the conjugate gradient algorithms. All systems underwent first a 30 ps constant volume temperature equilibration to reach 293 K in contact with the Langevin thermostat. [11] Subsequently, equilibration was performed with a constant number of particles and constant temperature and pressure (NTP) with the target temperature

of 293 K, and volume altered to reach on average the 1 bar pressure target, with time-spans noted in table S1 (in the range 0.3 - 6 ns) using the Langevin thermostat. The particle-mesh Ewald method [12] was used for the long-range electrostatic interactions, with a cutoff at 9 Å. The simulations were performed with periodic boundary conditions, and the typical box dimensions are given in Table S1.

In the cluster simulations, quantum-mechanically computed forces replaced the classical force field for intra and inter surfactant interactions (treated as a QM region) for monomer to pentamer cluster. The QM region was embedded in the explicit SPC/Fw waters mentioned above. The QM region was modelled with the parameter model 3 (PM3) Hamiltonian, [13] a semiempirical QM method. Clearly, the weak hydrophobic interactions between surfactants may have a dependence on the QM method used and this is an active field of research. [14] To explore the dependence of QM method, the dimer surfactant system was simulated with the recently developed PM6-DH+ Hamiltonian, [14] particularly addressing the non-covalent interactions. However, within the NMR relaxation relevant data extracted here (see Table S2), no drastic difference was seen in carbon chain order and dynamics that would be anticipated to change the conclusions drawn and, thus, all the systems (1-5 surfactants) were simulated with the PM3 Hamiltonian.

Figure S4 shows snapshots of simulations of 1-5 surfactants clusters. The clusters remained intact over the 1-2 ns long simulations. It is noted that 3 and 5 surfactants clusters have headgroups on the opposite sides of the cluster.

Micelle simulations were done using an all-classical MM method, with force field parameters taken from the AMBER/GAFF (General Amber Force Field) that has turned out to be accurate in bilayer simulations. [15] To proceed with the MM simulations, the partial charges of the decanoate surfactant were computed with the antechamber module of AMBER and the semi-empirical method (AM1-BCC) prior starting the simulation. Representative snapshots of the simulations of the three larger aggregates (micelles) are shown in Fig. S5. The simulated micelles

remained intact over the 1-5 ns long simulations. The final relaxation model is based on the largest 67 surfactant simulation over 5 ns where 66 monomers remain in the micelle.

S1.2.2 Transition from MD simulation to NMR relaxation

Intra-cluster/molecule dipolar ^{1}H NMR relaxation rates are [16]

$$R_{1,\text{intra}}^{\text{HH}}(\omega_\text{H}) = \frac{3}{10} c_{\text{HH}}^2 \left[J_\text{intra}(\omega_\text{H}) + 4J_\text{intra}(2\omega_\text{H})\right], \tag{1}$$

and

$$R_{2,\text{intra}}^{\text{HH}}(\omega_\text{H}) = \frac{3}{20} c_{\text{HH}}^2 \left[3J_\text{intra}(0) + 5J_\text{intra}(\omega_\text{H}) + 2J_\text{intra}(2\omega_\text{H})\right], \tag{2}$$

where

$$c_{\text{HH}} = \left\{-\frac{\mu_0 {\gamma_\text{H}}^2 \hbar}{4\pi}\right\}, \tag{3}$$

$\hbar$ is the Planck constant in the angular units, μ_0 is the vacuum permeability and γ_H is the proton gyromagnetic ratio. The relaxation rates are linear combinations of spectral densities ($J_\text{intra}(\omega)$) evaluated at zero, once and twice the Larmor frequency (ω_H). Here, we assume that the spectral densities are sums of three Lorentzian functions:

$$J_\text{intra}(n\omega) = \sum_i s_i \frac{\tau_i}{1+(n\omega\tau_i)^2}, \tag{4}$$

where $s_i = \{a_1(1-S^2), a_2(1-S^2), S^2\}$, $a_2 = 1 - a_1$, and the total sum of weights s_i is unity (see Tables S2 and S3). Hence, the time correlation function (TCF), which is the inverse Fourier transform of the spectral density function, is represented as a sum of exponential functions. [16] For intramolecular dipole-dipole (DD) relaxation, the TCFs are computed as:

$$G^{pl}(\tau) = \sum_{i=1}^{Npl} \sum_{j=1}^{N} \sum_{k=-2}^{2} \left\langle \frac{D_{k0}^{(2)*}\left(\Omega_0^{ij}\right)}{\left(r_0^{ij}\right)^3} \frac{D_{k0}^{(2)}\left(\Omega_\tau^{ij}\right)}{\left(r_\tau^{ij}\right)^3} \right\rangle, \tag{5}$$

where each of the observable protons of type *pl* (*pl* = P1, P2, P3 and P4 in Fig. 1A) that are in total *Npl* have their own dipole-dipole TCF ($G^{pl}(\tau)$), with $D_{k0}^{(2)}$ second rank Wigner matrix elements [17] with time dependent Euler angles Ω_τ^{ij} of the vector connecting spins *i* and *j* and the corresponding distance (r_τ^{ij}) computed as a sum over all the protons (*N, i≠j*) in the cluster, where the bracket in Eq. 5 denote an ensemble average. Note, the distances are intramolecular for monomer and intra-cluster for a cluster/micelle. Furthermore, the initial point $G^{pl}(0)$, Eq. 5, is the ensemble average of inverse sixth power of distance that may be translated to an effective distance (r_{eff}).

Here we note that the MD simulations provide a strength compared to analytical relaxation models, [6] since molecular details are obtained without adjustable parameters and, in principle, Eq. 5 (and, consequently, Eqs. 1, 2) may be computed directly from MD simulations. However, achieving sufficient statistics and size of simulations poses a challenge for atomistic simulation techniques. [7,18,19] Due to this fact and for comparison with previous works, [6,7,19,20] and to allow for parameterized micelle size, it is valuable to analyze and fit the outcome of MD result of Eq. 5 in terms of a commonly used two-step model, [18–21]

$$G(t)/G(0) = \left[a_1 e^{-\frac{t}{\tau_1}} + (1-a_1)e^{-\frac{t}{\tau_2}}\right](1-S^2) + S^2 e^{-\frac{t}{\tau_{\text{eff}}}}, \qquad (6)$$

where the first bracket represents the fast dynamics with pico-second correlation times (τ_1, τ_2), and the final term the slow aggregate dynamics with residual order parameter S^2 weighting these two contributions. The parameter S^2 reflects the residual dipole-dipole order in a local molecular frame [22] and is here determined from the TCFs. To avoid the need for exceptionally long simulations of the smaller clusters (1-5 surfactants), we assume that the longest correlation time and the slowest translational diffusion of the intact clusters are due to overall cluster reorientation ($\tau_{\text{eff}} = \tau_{\text{R}}$) and mobility (we discuss the micelle dynamics below). Correlation time τ_{R} and translational diffusion

coefficient D_{tr} are accessible through Stokes-Einstein-Debye [23] (SED) and Stokes-Einstein [24] (SE) equations, respectively:

$$\tau_{\mathrm{R}} = \frac{4\pi\eta R_{\mathrm{H}}^3}{3k_{\mathrm{B}}T} \quad (7)$$

$$D_{\mathrm{tr}} = \frac{k_{\mathrm{B}}T}{6\pi\eta R_{\mathrm{H}}}. \quad (8)$$

Here, T is the temperature, η is the viscosity (0.890 and 1.095 mPa·s at 298 K and 1 bar for H_2O and D_2O, respectively), [25,26] R_H is the hydrodynamic radius and k_B is the Boltzmann constant. The diffusion coefficient can also be computed from the mean square displacements (MSQD) extracted from the MD trajectories:

$$D_{\mathrm{tr}} = \lim_{t\to\infty} \langle |r(0) - r(t)|^2 \rangle / 6t, \quad (9)$$

where $r(t)$ is one atom position within the cluster. An estimate of hydrodynamic radius $\tilde{R}_{\mathrm{H}}$ for flexible polymer can be computed with [27]

$$\tilde{R}_{\mathrm{H}}^{-1} = \langle \frac{1}{|r_m - r_n|} \rangle, \quad (10)$$

where the ensemble average is over the possible atom pairs $m \neq n$ and averaged over MD-trajectory snapshots of the surfactants in cluster (Boltzmann configurations). $\tilde{R}_{\mathrm{H}}$ is convenient to compute to estimate the size of a cluster, however, it does not contain counter-ions and solvent shell and may thus underestimate the radius in comparison with the SE equation (Eq. 8). In order to reduce computational cost but still obtain accurate estimate a value from Eq. 10 is calibrated with Eq. 8 below.

S1.2.3 Small cluster MD results

In Fig. S6, the fits of Eq. 6 with the MD simulated DD-TCF of Eq. 5 are given. For the monomer, adjustable parameters are a_1, τ_1, τ_2 and τ_{eff} with S^2 explored with a grid of values. The dimer to

pentamer clusters were analyzed with fixed $\tau_{\mathrm{eff}} = \tau_{\mathrm{R}}$ using Eq. 7 and $\tau_{\mathrm{R}}(\text{multimer}) = \tau_{\mathrm{R}}(\text{monomer}) \times (\tilde{R}_{\mathrm{H}}^{3}(\text{multimer})/\tilde{R}_{\mathrm{H}}^{3}(\text{monomer}))$. In this way, we rely on the trend in size dictated by $\tilde{R}_{\mathrm{H}}$ and reduce the need for long simulations to capture the slowest decay rate in detail. As noted above, all simulations were done with H_2O and the timescale of ensemble averages were scaled with the ratio of D_2O and H_2O viscosities given above.

In Figs. S7A and S7B, the MSQD for water and monomer are displayed, and the figure illustrates also the estimation of diffusion constant with Eq. 9. The diffusion coefficients of dimer to pentamer were computed from $D_{\mathrm{tr}}(\text{monomer}) \times [\tilde{R}_{\mathrm{H}}\,(\text{monomer})/\tilde{R}_{\mathrm{H}}\,(\text{multimer})]$, hence, removing the need to fit MSQD (Fig. S7C). The translational diffusion and TCF data are summarized in Table S2.

Based on the MD simulation, the water diffusion constant was estimated to $1.86 \cdot 10^{-9}\ \mathrm{m^2 s^{-1}}$ (see Fig. S7A, using the water data of the dimer simulation in Table S1). This is in good agreement with the experimentally observed water diffusion coefficients (see Fig. 1C from the main text) and with the D_2O diffusion coefficient of $1.7 \cdot 10^{-9}\ \mathrm{m^2 s^{-1}}$ recorded at 295 K, [28] and points towards relevant dynamics in the MD simulation and no significant bias introduced by rescaling the MD simulation to D_2O viscosity for a dilute surfactant concentration.

From Table S2, it is noted that monomer diffusion coefficient is 60% higher than the low concentration experimental diffusion coefficient (see the first panel in Fig. 1C from the main text). Furthermore, it is seen that r_{eff} decrease and S^2 increases with larger cluster. This can be expected with an increased density of surfactants and leads to an increased contribution of slow dynamics in a modelling of relaxation rates. The slowest dynamics are in the range of sub nanoseconds (see τ_{R}).

S1.2.4 Micelle MD results

The MD simulated DD-TCFs and MSQD (Eq. 5) of the 66-surfactant micelles are shown in Fig. S8, along with the fits of Eqs. 6 and 9 for extracting parameters (shown in Table S3). There is some variability in the DD-amplitude and corresponding r_{eff} distance as well as in effective order parameter S. It is noted that r_{eff}, due to the inverse power 6 average, is dominated by the closest intra-surfactant proton distance (approx. 1.7 Å). The radius R_{H}, determined from Eq. 8, and therefore the MSQD estimated diffusion constant is larger than the one estimated with Eq. 9. This can be expected since R_{H} is affected by close ions and solvent in addition to the surfactants.

To avoid the need to simulate every surfactant concentration and also to bring physical insight to the MD micelle results (Eq. 5), the correlation time τ_{eff} in the parameterized form is discussed. Often in the literature of analytical models, [29–31] plausible explanation of slower dynamics relevant to the DD-relaxation is the micelle overall reorientation (parameterized in Eq. 7) and surfactant interface diffusion (D_{S}). With these processes describing slower dynamics we have [31]

$$\frac{1}{\tau_{\text{eff}}} = \frac{1}{\tau_{\text{D}}} + \frac{1}{\tau_{\text{R}}};\ \tau_{\text{D}} = \frac{R_{\text{H}}^2}{6D_{\text{S}}}, \tag{11}$$

where τ_{D} is the surfactant surface diffusion correlation time, τ_{R} is given by Eq. 7 (~30 ns) and D_{S} is the interface surfactant translational diffusion [30] (on a curved spherical micelle interface). The observations from TCFs in Figs. S8A and S8B are that they decay a factor of 10 faster than τ_{R}, meaning τ_{D} plays a dominating role. One approach to determine D_{S} is as an adjustable parameter in fitting Eq. 6 to MD computed TCFs. However, here we consider the estimate of D_{S} based on MD simulation of 66-surfactant micelle (Table S3), by fixating the center of the mass of the micelle and from the initial slope of head group MSQD (= $4D_{\text{S}}t$ for a surface process) that is dominated by interface surfactant translational diffusion, to find the value: $D_{\text{S}} = 6.7 \cdot 10^{-10}$ m^2s^{-1}. This gives $\tau_{\text{D}}(R_{\text{H}}) = 2$ ns and is kept fixed in analyzing TCFs in Fig. S6. The D_{S} is in the regime of free surfactant diffusion and therefore τ_{D} the more important slow process for DD-relaxation (see Eq. 11).

Due to finite time of the MD simulation, there may be additional explanations of slow dynamics, for instance, surfactant protrusion or exchange that is slower than the ns-regime covered by MD simulation, as discussed and explored within model 3 and a 160 ns MD trajectory below.

S1.2.5 Parameter estimation from the relaxation and diffusion data

The low and high concentration (below and above the CMC) experimental relaxation and diffusion data (denoted *EXP*) were analyzed separately. Models are formulated in the following sections, containing a few adjustable parameters ($\boldsymbol{x}$) that are estimated from *EXP*. Details on the method used can be found from our previous work [32] and the review article. [33] To summarize, the parameter distribution (a conditional probability distribution) $P(\boldsymbol{x}|EXP, I)$ given by the experimental data was sought, where I denotes prior assumptions. The Bayes theorem was used to relate the model parameterization x given the experimental data *EXP* and the prior assumptions I to the experimental likelihood $P(EXP|\boldsymbol{x}, I)$:

$$P(\boldsymbol{x}|EXP, I) \propto P(EXP|\boldsymbol{x}, I)P(\boldsymbol{x}|I), \tag{12}$$

where $P(\boldsymbol{x}|I)$ is the prior assumption on $\boldsymbol{x}$. [33] The probability distribution of the parameterization $\boldsymbol{x}$ was sampled by Markov Chain Monte Carlo (MCMC), [32] and it allowed both a parameter estimate (mean of $P(\boldsymbol{x}|EXP, I)$) and their confidence interval to be computed. The "uninformed" prior was used, where, prior to data processing, a minimum and maximum of x was chosen with uniform probability between these constraints. The experimental likelihood takes the form $P(EXP|\boldsymbol{x}, I) = \exp(-\chi^2)$, where χ^2 is the sum of square cost-function:

$$\chi^2 = \frac{1}{2}\sum_{i=1}^{N\exp} \frac{(EXP_i - MODEL_i)^2}{\sigma_i^2}, \tag{13}$$

N_{exp} is the number of experimental data, (σ_i^2) is the uncertainty (variance) of an experimental observation i estimated for the experimental data. The uncertainty in diffusion was set to be the

standard error of the diffusion coefficients measured independently for the four ^{1}H signals of the surfactant. The uncertainties of relaxation rates R_1 and R_2 were estimated from the fits of experimental data with exponentially dependent function and extracting the standard error reported in Origin 2018 software. The uncertainties extracted in this way typically underestimate the errors, but they were assumed to capture the relative size of uncertainties within (R_1, R_2)-data and, to be on the safe side, the uncertainties were multiplied by the factor of 3.

S1.2.6 Interpretation of low concentration relaxation/diffusion experiments

Figure S3 shows a significant variability in the $R_2 = 1/T_2$ and a significant difference between R_2 and R_1 below the CMC (above $C^{-1} = 0.01$ mM^{-1}). Given that the simulated monomer diffusion coefficient is about 60% higher that the experimental value, we assume that clusters (A) and monomers (B) exist together, there is a fast exchange between these two sites, and the observed relaxation rates and diffusion coefficients are the weighted averages of those in the two sites:

$$R_i^{\mathrm{model}}(\omega_{\mathrm{H}}) = X_{\mathrm{A}}\left[R_{i,\mathrm{intra}}^{\mathrm{HH,A}}(\omega_{\mathrm{H}}) + R_{i,\mathrm{inter}}^{\mathrm{HY,A}}(\omega_{\mathrm{H}})\right] + (1 - X_{\mathrm{A}})\left[R_{i,\mathrm{intra}}^{\mathrm{HH,B}}(\omega_{\mathrm{H}}) + R_{i,\mathrm{inter}}^{\mathrm{HY,B}}(\omega_{\mathrm{H}})\right], \quad (14)$$

and

$$D^{\mathrm{model}} = X_{\mathrm{A}} D^{\mathrm{A}} + (1 - X_{\mathrm{A}}) D^{\mathrm{B}}. \quad (15)$$

Eq. 14 represents the observed longitudinal and transverse relaxation rates (i=1,2) and Eq. 15 models the observed translational diffusion coefficient, and X_{A} is the cluster molar fraction. In Eq. 14, the intra molecular DD contribution is included (see Eqs. 1 and 2), along with the inter DD contribution between the surfactant protons (H) and solvent (Y={D,H}). The diffusion driven inter-DD relaxation is dependent on closest spin contact distance, which was estimated from MD to be about 2.2 Å, with details in our previous work and textbook. [16,32] However, although the closest contact distance was short, the inter-DD contribution had an almost negligible contribution.

In Fig. S9, a monomer model ($X_{\mathrm{A}} = 0$ in Eqs. 14 and 15) without adjustable parameters for the diffusion and relaxation data at low surfactant concentration is shown. The model overestimates diffusion coefficients and have the equal R_1 and R_2 values, which is contrary to the experimental observations, where $R_1 < R_2$. These observations contradict the conventional model, which assumes that only monomers exist below the CMC [34]. It is noted that chemical shift anisotropy is in the regime of a few ppm for protons and gives a minor relaxation contribution, less than the included inter-DD mechanism and cannot support the monomer model. The studied protons do not undergo chemical exchange and therefore the exchange is not included in the model. In principle, one could make a new water force field [10] to slow down the dynamics (for instance with a drastic change of water partial charges) and this could give a better agreement of the monomer model, but this would lead to a significant underestimation of water diffusion (that is currently well explained), so it is not a justified way to explain the experimental data.

Figure S10 displays the combination of monomer and pentamer in the model (Eqs. 14 and 15), with the mole fraction X_{A} as the only adjustable parameter (bounded between 0 and 1). This model improves the fit significantly (χ^2 decreased by factor 28) as compared to the monomer model, and, contrary to the monomer model, $R_1 < R_2$ like in the experiments. Therefore, the model indicates that small clusters exist below the CMC. Clearly, the fit could be improved and there may be more than two aggregate sizes present and/or a rate of aggregate breakup and formation. Earlier MD simulations of smaller surfactants (sodium octanoate) suggested also that the monomers are not stable, and, in addition to larger and relatively stable micelles, there are smaller clusters, which are continuously formed and broken in the simulation time scale (20 ns). [35] However, to the best of our knowledge, this is the first combined experimental and modelling observation of the discrepancy of the monomer model.

S1.2.7 Interpretation of relaxation/diffusion experiments above the CMC

Above the CMC (below C^{-1} = 0.01 mM^{-1}), R_2 and D is approximately linearly dependent on C^{-1} (see Fig. S3). Three models were tested for explaining the observed dependence. Model 1 assumes two surfactant populations: the first within larger micelles of fixed size and the second within smaller clusters. Model 2 assumes that all surfactants form micelles, *i.e.*, there are no monomers or clusters, and the size of micelles increases with concentration as calculated by Stokes-Einstein equation from D. Model 3 is otherwise identical to model 1, but it accounts for the effect of molecular protrusion/exchange. Common for all the models are the use of MD parameterization. All the models describe combined translational diffusion and (R_1, R_2) relaxation data.

Model 1 follows Eqs. 14 and 15, but here A and B refers to the micelles and small clusters, respectively. The conventional model assumes monomers coexisting with micelles, [34] but, as the modelling results below the CMC suggest that the monomers are not stable, small clusters are assumed instead of monomers in Model 1. Following the conventional model, the concentration of surfactants in small clusters, C_B, is fixed to the CMC. Therefore, the mole fraction of surfactants in micelles is $X_A = (C - C_B)/C$, where C is the total concentration, and the mole fraction of surfactants in the smaller cluster is $X_B = 1 - X_A$.

As the diffusion delay Δ is about 100 ms, micelles and clusters move over a micrometer during the diffusion experiment, and thus other micelles may hinder their diffusion at the higher concentrations. If the solution includes larger and smaller spherical particles (in this case larger micelles A and smaller clusters B), the diffusion coefficients D_A and D_B decreased due to the geometric obstruction are given by [7,36]

$$\frac{D_A}{D_A^0} = (1 + 2\Phi_A)^{-1}; \ \frac{D_B}{D_B^0} = (1 + 0.5\Phi_A)^{-1}. \quad (16)$$

Here, D_A^0 and D_B^0 are the diffusion coefficients micelles and clusters without the obstructing particles, and $\Phi_A = \Phi X_A$ is the volume fraction of micelles, where Φ is the total volume fraction of surfactants,

varying from 0.03 to 0.13 when the concentration increases from 153 to 704 mM. It is noted that for ionic surfactants, as in this study, the obstruction may be larger than the geometry constraint due to the additional interactions. [7]

Model 1 (Eqs. 14 and 15) incorporates information from the MD simulation of the 66-surfactant micelle (Table S3), from where the fast dynamics of intramolecular DD relaxation and effective order parameter S is obtained. The intermolecular DD solvent contribution is found to be less than 1% of the intramolecular DD contribution. The average closest contact of 5 Å for protons P1-P4 is used for simplicity. The amplitude of TCF in Eq. 5 and subsequent relaxation rates has the sixth power inverse dependence on r_{eff} that is known from MD simulation. Due to the r_{eff}^{-6} dependence and the possibility that MD simulations with other force fields might give slightly different outcome, we allowed for some variability in the picometer range for r_{eff}. However, it is not physically reasonable that r_{eff} would be larger than that for the monomer (see Table S2), setting the upper boundary for r_{eff}. The lower boundary is chosen lower than three statistical standard errors reduction. The micelle radius is an adjustable parameter fixating the slow dynamics (τ_{eff}), where the interface diffusion constant is determined from the MD simulation (see Eq. 11).

The model including small pentamer clusters along with the larger micelles gave the best fit (see Fig. S11). The resulting parameter values and their 95% confidence interval are given in Table S4. Model 1 captures the main trends of experimental data, overestimating the diffusion coefficient at high concentration by 40% and systematically overestimating the relaxation rates. The fitted r_{eff} is larger than the MD simulated result and leads to a reduction of the relaxation rates compared to the MD simulated result. The determined micelle radius is 26.6 Å and the dominating slow process for DD-relaxation is the interface translational diffusion with the correlation time τ_{D} in the 1 ns regime. Apart from a bias in relaxation rate, Model 1 captures the trend of data well. It is noted that the field dependence at high fields for the model is small (a largest difference in R_1 of 0.05% comparing 9.4 and 11.75 T), consistent with the small difference for experimental data at 90%

D_2O solvent composition. The χ^2 is large, which motivates the consideration of one more process discussed in Model 3 below.

Model 2 assumes all the surfactant monomers form micelles above CMC with the radius consistent with that calculated by the Stokes-Einstein relation (Eq. 8) based on the experimentally observed diffusion coefficient. The radius is in the range 6.3-23 Å, when the geometric obstruction factor is taken into account (6.7-30 Å if dilute sample is assumed). The model follows from Eqs. 14 and 15 with $X_\mathrm{A} = 1$. The model of slow dynamics follows Eq. 11 with τ_D (dependent on the radius and interface diffusion) replaced by a constant τ_eff, since this gives a lower χ^2. A physical motivation for τ_eff may be a similar reorganization of surfactants in the micelle, however, not driven by interface diffusion process. The fitted parameters are given in Table S5 and the best fit illustrated in Fig. S12. The diffusion agreement is by definition exact (see Fig. S12), however, the overall agreement is worse than in the case of Model 1 (by a factor of 2.5 in χ^2). The main shortcoming are too large relaxation rates, which cannot be fixed by changing the effective proton distance, because it is unrealistic to increase boundary further than given by a single shortest proton distance. Removing the obstruction gives a slight increase in χ^2.

Model 3. Models 1 and 2 are fixated at too large values of relaxation rates. This limitation is alleviated by Model 3 considering a process, which is not available in the five nanoseconds long MD simulations. Figure S13 illustrated how the surfactant may partially leave the micelle. Such process is postulated as molecular protrusion. [37,38] Similar effect may be achieved with surfactant leaving in an exchange process. If the protrusion is slow, it provides a relaxation pathway not accounted for by current MD simulation with a few ns-timespan. In Model 3, the micelle part A in Eq. 14 is extended with a surfactant residence time at micelle:

$$R_{i,\mathrm{intra}}^{\mathrm{HH,A}}(\omega_\mathrm{H}) \rightarrow (1-f)R_{i,\mathrm{intra}}^{\mathrm{HH,mono}} + fR_{i,\mathrm{intra}}^{\mathrm{HH,A}}(\omega_\mathrm{H}), \quad (17)$$

where f is the fraction of micellar surfactants that undergo the micelle relaxation of Model 1, with the effective correlation time (see also Eq. 11) of $\frac{1}{\tau_{\text{eff}}} = \frac{1}{\tau_{\text{D}}} + \frac{1}{\tau_{\text{R}}} + \frac{1}{\tau_{\text{m}}}$, where τ_{m} is the residence time characterizing molecular protrusion. For the fraction temporarily not constrained by micelle $(1-f)$, a smaller relaxation rate known from monomer MD simulation (Table S2) is assumed. The same small pentamer clusters (B) (see Eq. 14) as in Model 1 are considered.

The adjustable parameters for Model 3 are provided in Table S6, showing slightly larger radius at 27 Å as compared to Model 1. With the given relaxation data, a precise estimate of τ_{m} is not possible; one can just deduce that it is slower that than τ_{D} (1.8 ns). The latter is also the dominating (and micelle size dependent) correlation time. The best fits are provided in Fig. S14, overall matching the data well, with a 40% overestimation of translational diffusion at 700 mM concentration.

Additional MD trajectory of 160 ns length was generated to find support or disprove the hypothesis of protrusion/exchange in Model 3 and Eq. 17. The statistical estimate of residence time of surfactant within 35 Å of center of mass of micelle ($\tau_{\text{m}} = 155$ ns) and outside the same boundary ($\tau_{\text{o}} = 25$ ns). Based on these two sites, the fraction of surfactants within micelle is given by [39] $f = \frac{\tau_{\text{m}}}{\tau_{\text{m}}+\tau_{\text{o}}} = 0.86$. Note that this represents an exchange process with surfactant geometrically being outside the micelle. The details of protrusions contain the additional choice as how to set the MD selection criteria and is not explored here. The data gives support to the hypothesis of Model 3, with MD suggesting a long residence time and largest fraction 0.7. Considering the model result (Table S6), the higher fraction at the CH_3 part of the chain is consistent with this group having less probability of leaving the micelle in a protrusion/exchange event.

S1.2.8 Model comparison

In the above relaxation study, we aimed at reducing the number of adjustable parameters and conventional relaxation model assumptions by conducting molecular dynamics simulations.

However, there are restrictions in what can be simulated with sufficient statistics (multiple aggregates in same simulation *etc*.), and input from MD are merged with analytical relaxation model contributions. In particular transition from Model 1 to 3 involves to large extent the same physics description, where Model 3 assumes that there are dynamics, which we have not covered in the MD but the postulate [37] and parameterization accounts for it. Note, that Model 3 has significantly more parameters in addition to a constrained DD-amplitude ($\boldsymbol{r}_{eff}$). This makes it interesting to compare Models 1 and 3 in a Bayesian sense, to get a handle on if we have a significantly better model given the data or if it is just because of more parameters that the fit has improved. [40]

As described in Refs. [40] and, [41] we can consider the probability of model-j (M_j) as

$$P(M_j|EXP) = P(EXP|M_j) \cdot P(M_j), \qquad (18)$$

where left hand side is the probability of model M_j given the experimental data and right side is the so-called Bayesian evidence (also denoted marginal likelihood) times the prior probability of M_j. [40] The latter is the probability of the model prior to any data analysis. It is assumed that the prior probability is same for Models 1 and 3. Due to this assumption, by computing the ratio of Bayesian evidence, we obtain the sought probability of the two models (see Eq. 18). The logarithm of the Bayesian evidence is given by [40]

$$\ln[P(EXP|M_j)] = -\frac{1}{2}\int_0^1 \langle \chi^2(\boldsymbol{x}_j)\rangle_\beta d\beta, \qquad (19)$$

using the cost-function (Eq. 13); β is the "temperature" in a thermodynamic integral and the bracket represent an integral over parameter space ($\boldsymbol{x}$) at the temperature β. The integrand is computed with MCMC and is displayed in Fig. S15.

The Bayesian evidence computed for Models 1-3 in Table S7 by numerically integrating the MCMC output in Fig. S15. The Bayesian ratio [40] ($\ln[\frac{P(EXP|M_3)}{P(EXP|M_1)}]$) for model 1 and 3 is larger than 10^5 and provides decisive evidence for model M_3 in a statistical sense. [41] There are big differences in

the Bayesian evidence (Table S7), and this can be understood from the differences in quality of fit (χ^2_{opt}) and the narrow-explored parameter intervals constrained by the molecular arguments (discussed above).

S1.3 Atmospheric modeling

S1.3.1 Köhler theory

The process of cloud droplet equilibrium growth and activation is described by Köhler theory. [42,43] The Köhler equation gives the equilibrium water vapor saturation ratio S as a function of the aqueous droplet diameter d

$$S \equiv \frac{p_{\text{w}}}{p_{\text{w}}^0} = a_{\text{w}} \exp\left(\frac{4\overline{v_{\text{w}}}\sigma}{RTd}\right), \tag{20}$$

where p_{w} is the equilibrium partial pressure of water over the solution droplet, p_{w}^0 is the saturation vapor pressure over a flat surface of pure water, a_{w} is the droplet solution water activity, $\overline{v_{\text{w}}} = M_{\text{w}}/\rho_{\text{w}}$ is the partial molar volume of water, σ is the droplet surface tension, R is the universal gas constant, and T is the temperature in Kelvin. The presence of solutes decreases the water activity and vapor pressure of water in the droplet phase, according to the Raoult effect. At the same time, the vapor pressure over a positively curved interface is enhanced compared to over a flat surface of the same substance. This is also known as the curvature or Kelvin effect and described by the exponential term of Eq. 20. Together, these two effects define a critical point $(d_{\text{c}}, S_{\text{c}})$ at the maximum of the Köhler growth curve, which constitutes the threshold for diffusion limited droplet growth. The critical water saturation is also referred to in terms of the critical supersaturation $\text{SS}_{\text{c}} = (S_{\text{c}} - 1) \cdot 100\%$. Each droplet is assumed to form by water condensation on a spherical dry particle with diameter D_{p}. The dry particles are assumed to consist purely of sodium decanoate (NaDec). Using a single-species proxy is typical in aerosol process studies, to ensure well-defined compound properties and eliminate

confounding chemical interactions. Decanoic acid and its corresponding base decanoate are used to represent surface active atmospheric aerosol and in particular abundant fatty acids in several previous works.[43–48] As the droplet grows by condensation of water (w) vapor, the total (T) amount of sodium decanoate $n_{\mathrm{NaDec}}^{\mathrm{T}}$ in the droplet phase remains constant. The total amount of water $n_{\mathrm{w}}^{\mathrm{T}}$ at each droplet diameter d is determined iteratively, by assuming volume additivity for components in droplet phase and describing the droplet solution density as a weighted average of the composition.[43,49]

S1.3.2 Bulk-surface partitioning model

The surface activity of sodium decanoate in aqueous solutions is taken explicitly into account at all stages of droplet growth by calculating the bulk–surface partitioning and droplet surface tension resulting from surface adsorption using the molecular monolayer (ML) model of Malila and Prisle.[49] The model has previously been successfully applied for a range of surface-active aerosol components and conditions of cloud droplet formation.[44,49–53] At each diameter, the growing aqueous droplet is divided into a surface monolayer of thickness δ and an interior bulk phase with diameter $d - 2\delta$. The surface is described as a separate liquid phase with a composition distinct from the bulk. The compositions of the droplet bulk (B) and surface (S) are calculated from the partitioning of each droplet component $j = \{\mathrm{w,NaDec}\}$ between these phases. This is done iteratively using the semi-empirical relation between the droplet surface tension σ, parameterized in terms of the composition of the bulk, Eq. 35, and weighted by volume of components in the surface as

$$\sigma(\boldsymbol{x}^{\mathrm{B}}, T) = \frac{\sum_j \sigma_j v_j x_j^{\mathrm{S}}}{\sum_j v_j x_j^{\mathrm{S}}}, \quad (21)$$

where $\boldsymbol{x}^{\mathrm{B}}$ is the bulk composition vector, v_j is the liquid phase molecular volume, σ_j the surface tension (at T = 25°C), and x_j^{S} and x_j^{B} are the mole fractions of component j in the droplet surface and bulk phase, respectively. The thickness of the surface monolayer is

$$\delta = \left(\frac{6}{\pi}\sum_j v_j\, x_j^S\right)^{1/3}. \qquad (22)$$

Mass conservation ($n_j^{\mathrm{T}} = n_j^{\mathrm{S}} + n_j^{\mathrm{B}}$) is imposed for each compound j in the droplet as a boundary condition. An ionic surfactant is assumed to partition to the droplet surface in its undissociated form to preserve charge neutrality of each phase. For NaDec, this means that surface adsorption does not lead to excess free sodium ions (Na^+) in the bulk phase.

S1.3.3 Water activity models

For the calculation of the water activity term in Eq. 20, we use several approaches to account for the formation of micelles and/or clusters in the growing aqueous droplets. The bulk–surface partitioning is evaluated for NaDec monomers because experimental surface tension isotherms are typically given in terms of total monomer concentration in the bulk, regardless of the actual solution state. [44,45,49–53] Therefore, the total amount of NaDec in the bulk phase $n_{\mathrm{NaDec}}^{\mathrm{B}}$, and the surface tension σ, at a given droplet size and total composition $\{n_{\mathrm{w}}^{\mathrm{T}}, n_{\mathrm{NaDec}}^{\mathrm{T}}\}$ are identical for all water activity calculations.

Effective water mole fraction. The droplet water activity can be approximated as a corrected water bulk mole fraction $x_{\mathrm{w}}^{\mathrm{B}}$ with different assumptions regarding the dissolution state and aggregate formation of NaDec in the droplet bulk phase. A conceptual representation of these assumptions is shown in Fig. 3A in the main text. In each case, NaDec dissolved in droplet bulk phase is assumed to be fully dissociated into sodium and decanoate (Dec^-) ions, NaDec ⟶ Na^+ + Dec^-.

In the first model (Mono), all NaDec exists as dissociated monomers up to the CMC. At concentrations above the CMC, the droplet bulk is assumed to contain an amount of dissolved NaDec equal to the CMC, $n_{\mathrm{NaDec,CMC}}^{\mathrm{B}}$, while excess NaDec above the CMC remains undissolved and has negligible impact on the droplet volume and solution state. [43] While CMC is constant for all droplets, the maximum amount of dissolved NaDec increases as the droplet volume grows with

increasing d until all NaDec in the bulk phase is dissolved, $n^{B}_{NaDec,CMC} \geq n^{B}_{NaDec}$. The water activity is then calculated as

$$a_w = \frac{n^{B}_{w}}{n^{B}_{w} + n^{B}_{Na^+} + n^{B}_{Dec^-}} \tag{23}$$

where $n^{B}_{Na^+} = n^{B}_{Dec^-} = n^{B}_{NaDec} \leq n^{B}_{NaDec,CMC}$.

In the second model (MonoMic), the amount of NaDec exceeding the CMC is assumed to form micelles (Mic) co-existing with the monomers in the droplet bulk. The number n_m of Dec^- monomers is assumed to be the same for all micelles and independent of the NaDec concentration. Based on NMR measurements, we therefore use the constant value $n_m = 48$ for all micelles during droplet growth. The water activity is calculated as

$$a_w = \frac{n^{B}_{w}}{n^{B}_{w} + n^{B}_{Na^+} + n^{B}_{Dec^-} + n^{B}_{Mic}} \tag{24}$$

where n^{B}_{Mic} is the number of micelles in the droplet bulk. Each NaDec monomer in the droplet bulk is assumed to dissociate, including those forming micelles above the CMC. Therefore, $n^{B}_{Na^+} = n^{B}_{Dec^-} + n_m \cdot n^{B}_{Mic} = n^{B}_{NaDec}$ and $n^{B}_{Dec^-} \leq n^{B}_{NaDec,CMC}$.

In the third model (Clu), all NaDec in the droplet bulk is assumed to form clusters. Each cluster (Clu) consists of n_c Dec^- monomers and for all clusters during droplet growth we use a constant aggregation number $n_c = 5$ based on NMR measurements. Similar to the Mono model, excess NaDec above the CMC is assumed to remain undissolved and have negligible impact on the droplet volume and solution state. The water activity is calculated as

$$a_w = \frac{n^{B}_{w}}{n^{B}_{w} + n^{B}_{Na^+} + n^{B}_{Clu}}, \tag{25}$$

where n^{B}_{Clu} is the number of clusters in the droplet bulk and $n^{B}_{Na^+} = n_c \cdot n^{B}_{Clu} \leq n^{B}_{NaDec,CMC}$.

In the fourth model (CluMic), NaDec in the droplet bulk forms clusters at concentrations below the CMC, which co-exist with micelles above the CMC. The droplet water activity is calculated as

$$a_{\mathrm{w}} = \frac{n_{\mathrm{w}}^{\mathrm{B}}}{n_{\mathrm{w}}^{\mathrm{B}} + n_{\mathrm{Clu}}^{\mathrm{B}} + n_{\mathrm{Mic}}^{\mathrm{B}} + n_{\mathrm{Na^+}}^{\mathrm{B}}} \tag{26}$$

where $n_{\mathrm{Na^+}}^{\mathrm{B}} = n_{\mathrm{c}} \cdot n_{\mathrm{Clu}}^{\mathrm{B}} + n_{\mathrm{m}} \cdot n_{\mathrm{Mic}}^{\mathrm{B}} = n_{\mathrm{NaDec}}^{\mathrm{B}}$ and $n_{\mathrm{c}} \cdot n_{\mathrm{Clu}}^{\mathrm{B}} \leq n_{\mathrm{NaDec,CMC}}^{\mathrm{B}}$.

Modified number of free sodium ions in the droplet bulk. In addition to the above, we have modified the amount of free of Na^+ counterions in the bulk solution of the CluMic model to account for electrostatic association to the clusters and/or micelles. This model is designated CluMicNa. A simple parameterization of the fraction of free sodium ions, $f_{\mathrm{Na^+}}$, was constructed based on linear interpolation of $f_{\mathrm{Na^+}}$ by ^{23}Na NMR relaxation measurements and associated quadrupolar relaxation modelling provided in Table S8. [54] At NaDec bulk concentrations below 50 mM, $f_{\mathrm{Na^+}} = 1$ and above 700 mM, $f_{\mathrm{Na^+}} = 0.41$. Figure 18 shows the NMR data from Table S8 and the linear interpolation with these boundary conditions. The droplet water activity is then calculated as

$$a_{\mathrm{w}} = \frac{n_{\mathrm{w}}^{\mathrm{B}}}{n_{\mathrm{w}}^{\mathrm{B}} + n_{\mathrm{Clu}}^{\mathrm{B}} + n_{\mathrm{Mic}}^{\mathrm{B}} + f_{\mathrm{Na^+}} n_{\mathrm{Na^+}}^{\mathrm{B}}}, \tag{27}$$

where, as for the CluMic model, $n_{\mathrm{Na^+}}^{\mathrm{B}} = n_{\mathrm{c}} \cdot n_{\mathrm{Clu}}^{\mathrm{B}} + n_{\mathrm{m}} \cdot n_{\mathrm{Mic}}^{\mathrm{B}} = n_{\mathrm{NaDec}}^{\mathrm{B}}$ and $n_{\mathrm{c}} \cdot n_{\mathrm{Clu}}^{\mathrm{B}} \leq n_{\mathrm{NaDec,CMC}}^{\mathrm{B}}$.

The CISA activity model. In addition to the corrected water mole fraction models, we also calculate droplet water activity along the Köhler growth curves using the CMC-based Ionic Surfactant Activity model (CISA). [55] This comprehensive thermodynamic model was developed for estimating activity coefficients of components in binary and ternary aqueous ionic surfactant solutions and all model parameters required for binary aqueous NaDec are already available. The CISA model includes consideration of both dissociation and micellization of the ionic surfactants. Above the CMC, surfactant monomers MX form micelles (labeled as Mic) according to the reaction

$$q\mathrm{M}^{+} + n_{\mathrm{m}}\mathrm{X}^{-} = (\mathrm{M}_{q}\mathrm{X}_{n_{\mathrm{m}}})^{q-n_{\mathrm{m}}} = \mathrm{Mic}. \quad (28)$$

Based on the NMR observations, the micelle aggregation number is set to $n_{\mathrm{m}} = 48$, similar to the other models with micelle formation, and the number of counterions bound to each micelle is $q = 0.68n_{\mathrm{m}}$.[56] The activity of water is calculated as

$$a_{\mathrm{w}} = \exp\left[-\frac{\phi^{\mathrm{ion}}}{s}(m_{\mathrm{M}} + m_{\mathrm{X}} + m_{\mathrm{Mic}})\right], \quad (29)$$

where ϕ^{ion} is the osmotic coefficient, s is the molality of pure water, m_{M} and m_{X} are the molalities of the sodium and decanoate ions, respectively, in solution and m_{Mic} represents the molality of micelles. The reference state of each ion is a hypothetical 1 molal solution, for which the ratio $a_i m_i^{-1}$ tends to unity as the molality approaches zero.[57] The osmotic coefficient is given as

$$\phi^{\mathrm{ion}} = 1 + \frac{2}{\sum m_i}\left\{-\frac{A^{\phi}I^{3/2}}{1+b\sqrt{I}} + m_{\mathrm{M}}\left[m_{\mathrm{X}}\left(B_{\mathrm{MX}}^{\phi} + EC_{\mathrm{MX}}^{\phi}\right) + m_{\mathrm{Mic}}\left(B_{\mathrm{MMic}}^{\phi} + EC_{\mathrm{MMic}}^{\phi}\right)\right]\right\}, \quad (30)$$

where b is a model constant equal to 1.2 $\mathrm{kg}^{0.5}\mathrm{mol}^{-0.5}$ and I is the ionic strength calculated as

$$I = 0.5(m_{\mathrm{M}} + m_{\mathrm{X}} + \eta^2(q - n_{\mathrm{m}})^2 m_{\mathrm{Mic}}), \quad (31)$$

where $\eta = 0.5$ is shielding factor to reduce the effect of the micellar charge on the ionic strength of the solution. The equivalent electrical molality E is calculated as

$$E = m_{\mathrm{X}} + \eta(n_{\mathrm{m}} - q)m_{\mathrm{Mic}}. \quad (32)$$

The model parameters of the form C_{ca} and B_{ca} where "c" and "a" stand for cation and anion, respectively, account for pair and triple interactions between Na^{+} and Dec^{-} ions and Na^{+}ions and micelles. The parameter B_{ca}^{ϕ} is calculated as

$$B_{\mathrm{ca}}^{\phi} = \beta_{\mathrm{ca}}^{(0)} + \beta_{\mathrm{ca}}^{(1)}\exp(-\alpha_1\sqrt{I}), \quad (33)$$

where $\alpha_1 = 2.0\ \mathrm{kg}^{0.5}\ \mathrm{mol}^{-0.5}$ for all symmetric electrolytes. The model parameters C_{ca}^{ϕ} for Eq. 30 and β_{ca} for Eq. 33 are given in Table S9, where $\beta_{\mathrm{ca}}^{(0)}$ values are listed in the first row and $\beta_{\mathrm{ca}}^{(1)}$ values in the second row. The Debye-Hückel limiting slope $A^{\Phi} = A^{\gamma}/3$ is given as

$$A^{\gamma} = (2\pi N_{\mathrm{A}}\rho_{\mathrm{w}})^{\frac{1}{2}}\left(\frac{e^2}{4\pi\varepsilon_0 D k_{\mathrm{B}} T}\right)^{\frac{3}{2}}, \tag{34}$$

where k_{B} is the Boltzmann constant, N_{A} is the Avogadro number, T the temperature, ρ_{w} the density of water, [58] e the electron charge, D the dielectric constant of water [59] and ε_0 the permittivity of vacuum.

S1.3.4 Compound and solution properties

Properties of the aqueous droplet solutions and individual pure components used in the Köhler calculations are provided in the following. We do not explicitly consider pH in our calculations. To describe the slightly alkaline conditions of both sea water and aqueous aerosols in the marine environments, as well as the NMR experiments in the present work, we consider the decanoate-decanoic acid equilibrium to be overwhelmingly shifted towards decanoate ($pKa = 4.9$).[60] Molar masses, densities and surface tensions for pure water and sodium decanoate are given in Table S10.

Aqueous surface tension. We used the Langmuir-Szyszkowski equation with a parameterization scheme similar to that used by Prisle et al. [43] to calculate the surface tension of aqueous NaDec solutions as

$$\sigma(x, T) = \sigma_{\mathrm{w}} - a \ln\left(1 + \frac{m_{\mathrm{NaDec}}}{b}\right), \tag{35}$$

where m_{NaDec} is the bulk phase molality of sodium decanoate and $a = 10.1337\ \mathrm{mN\ m^{-1}}$ and $b = 8.696 \cdot 10^{-4}\ \mathrm{mol\ kg^{-1}}$ are model parameters. [61] The aqueous surface tension is constrained at a minimum value of $22.5\ \mathrm{mN\ m^{-1}}$, corresponding to the value of Eq. 35 at the CMC (Eq. 43).

Binary solution density. We calculate the composition-dependent density of a binary water–sodium decanoate solution with the method of Calderón and Prisle: [62]

$$\rho(T, m_{\mathrm{NaDec}}) = \frac{1+m_{\mathrm{NaDec}}M_{\mathrm{NaDec}}}{1/\rho_{\mathrm{w}}+1\cdot10^{-6}\Phi_{\mathrm{NaDec}}m_{\mathrm{NaDec}}}. \quad (36)$$

Here, m_{NaDec} and M_{NaDec} are the molality and molar mass of NaDec, respectively, ρ_{w} is the density of pure water (Table S10), and Φ_{NaDec} is the apparent partial molal volume of NaDec. The value of Φ_{NaDec} changes due to micellization according to

$$\Phi_{\mathrm{NaDec}} = H\,(\mathrm{CMC} - m_{\mathrm{NaDec}})\big(\Phi^{\infty}_{\mathrm{NaDec}} + A_v\sqrt{m_{\mathrm{NaDec}}} + B^{V}_{\mathrm{NaDec}}m_{\mathrm{NaDec}}\big) + H(m_{\mathrm{NaDec}} - \mathrm{CMC})\big((1-\xi)\Phi_{\mathrm{NaDec,CMC}} + \xi V_{\mathrm{Mic}}\big), \quad (37)$$

where the CMC is given in molality, $\Phi^{\infty}_{\mathrm{NaDec}}$ and $\Phi_{\mathrm{NaDec,CMC}}$ are the apparent molal volume in cm^3 mol^{-1} of NaDec at infinite dilution and at the CMC, respectively, H is the Heaviside step function, and ξ is the degree of micellization (Eq. 44). The model parameter B^{V}_{NaDec} is used to include the effect of surfactant–surfactant interactions with consistent units of cm^3 mol^{-2} kg. These model parameters are obtained from temperature-dependent parameterizations as

$$\Phi^{\infty}_{\mathrm{NaDec}} = 0.2762T + 82.06 \quad (38)$$

$$\Phi_{\mathrm{NaDec,CMC}} = 0.3004T + 75.65 \quad (39)$$

and

$$B^{V}_{\mathrm{NaDec}} = 0.002785T^2 - 1.648T + 244.5. \quad (40)$$

The variable V_{Mic} is the surfactant molal volume in the micellar form and depends on the molar volume change due to micellization as $V_{\mathrm{Mic}} = \Phi_{\mathrm{NaDec,CMC}} + \Delta V_{\mathrm{Mic}}$. The temperature-dependent parameter ΔV_{Mic} in units of cm^3 mol^{-1} is calculated as

$$\Delta V_{\mathrm{Mic}} = -0.1107T + 44.5. \quad (41)$$

The variable A_v represents the Debye-Hückel limiting slope in volumetric units $\left(\mathrm{cm}^3\ \mathrm{kg}^{1/2}\ \mathrm{mol}^{-3/2}\right)$, and is calculated as

$$A_v = 1.50619 + 0.0130073(T - 273.15) + 4.8307 \cdot 10^{-5}(T - 273.15)^2 + 8.95087 \cdot 10^{-7}(T - 273.15)^3 - 3.727 \cdot 10^{-9}(T - 273.15)^4 + 2.3942 \cdot 10^{-11}(T - 273.15)^5. \quad (42)$$

Critical micelle concentration (CMC). The temperature-dependent critical micelle concentration is estimated with the model of Blanco et al. [63] as

$$\ln x_{\mathrm{CMC}} = \ln x_{\mathrm{CMC}}^{*}\left\{1 + \beta_1 T^{*}\left[\frac{1}{2-\beta}\left(\frac{T^{*}}{T} - 2 - \frac{\beta_0 - 2}{\beta_1 T^{*}}\right) + \frac{1}{2-\beta^{*}}\left(1 + \frac{\beta_0 - 2}{\beta_1 T^{*}}\right)\right]\right\} + \frac{\Delta C_{\mathrm{P,m}}^{0*}}{(2-\beta)R}\left(1 - \frac{T^{*}}{T} - \ln\frac{T}{T^{*}}\right) + \frac{\alpha}{(2-\beta)R}\left(\frac{T^{*2} - T^{2}}{2T} + T^{*}\ln\frac{T}{T^{*}}\right) \quad (43)$$

where R is the ideal gas constant, T is the temperature (in K), T^* = 314.2 K, $\ln x_{\mathrm{CMC}}^{*} = -6.308$, $\Delta C_{\mathrm{P,m}}^{0*} = -507.4\ \mathrm{Jmol^{-1}\ K^{-1}}$, $\beta_0 = 0.06$ and $\beta_1 = 0.0052\ \mathrm{K^{-1}}$ while $\beta = \beta_0 + \beta_1 T$, and $\frac{\alpha}{R} = -1.6367\ \mathrm{K^{-1}}$.

The CMC of sodium decanoate in binary aqueous solution at 298.15 K is this way estimated as $x_{\mathrm{CMC}} = 0.002043$ [55].

Degree of micellization. In the MonoMic, CluMic, CISA and CluMicNa models, the extent or degree of micellization is quantified from the CMC in molal concentration as

$$\xi = \left(\frac{(\mathrm{sgn}(m_{\mathrm{NaDec}} - \mathrm{CMC}) + 1)}{2}\right)\frac{m_{\mathrm{NaDec}} - \mathrm{CMC}}{m_{\mathrm{NaDec}}} \quad (44)$$

where sgn is the sign function. [64] The variable ξ describes the fraction of the surfactant anions left in the droplet bulk after bulk–surface partitioning that forms micelles vs. remaining as monomers or clusters above the CMC, depending on the water activity model. An example of ξ as a function of droplet size for dry particles of D_{p}=50 nm is shown in Fig. S24C.

S1.3.5 Particle hygroscopicity

The droplet solution states and conditions predicted at the critical point of activation (d_c, S_c) will generally vary between droplets comprising different components. [51,65] To directly compare the various effects of micelles and clusters on droplet growth under the same humidity conditions, we evaluate the hygroscopic growth factor

$$\mathrm{HGF(RH)} = \frac{d(\mathrm{RH})}{D_\mathrm{p}}. \tag{S45}$$

The relative humidity RH is the ambient water saturation ratio S expressed as a percentage, RH = $S \cdot 100\%$. The equilibrium droplet size d corresponding to the growth of a dry particle with size D_p at a given RH is obtained from Eq. 20 under the different considerations of micelle and cluster formation and counter ion binding in the droplet phase.

The impact of specific particle components on water uptake can be quantified in terms of a single so-called hygroscopicity parameter κ. [66] We here evaluate κ for NaDec particles at different points of the Köhler curves predicted with each of the water activity models. Hygroscopicity parameters corresponding to the critical supersaturation of cloud droplet activation, are obtained similarly to Prisle et al. [67] from the κ-Köhler equation [66]

$$S = \frac{d^3 - D_\mathrm{p}^3}{d^3 - D_\mathrm{p}^3(1-\kappa)} \exp\left(\frac{4\overline{v}_\mathrm{w}\sigma}{RTd}\right), \tag{46}$$

by minimizing the distance between S_c calculated from Eq. 20 with the different water activity models and with Eq. 46 by varying both κ and the droplet diameter d independently. With this method, the d_c of the κ-Köhler curve from Eq. 46 may not match the critical point of the original Köhler curve as well as S_c (Fig. S22). This mimics the situation when κ values are obtained from laboratory or field measurements of S_c where the corresponding d_c is typically not known. For sub-saturated conditions, κ values are derived from HGF similarly to Jaatinen et al. [68] by reformulating Eq. 46 in terms of HGF (Eq. 45) and rearranging to solve for κ

$$\kappa = \frac{\mathrm{HGF}^3 - 1}{S} \exp\left(\frac{4\sigma M_\mathrm{w}}{RT\rho_\mathrm{w} D_\mathrm{p}\mathrm{HGF}}\right) + 1 - \mathrm{HGF}^3. \quad (47)$$

In the κ-Köhler formulation, all particle components are assumed to be either fully soluble or insoluble in the aqueous droplet. In the original formulation, κ is assumed to be constant across different humidity conditions. This has been both confirmed and disproved in subsequent work. [53,66,68–74] One factor leading to discrepancies between κ values obtained at different saturation conditions is that the droplet surface tension is typically assumed to be equal to that of pure water. For surface active species, the total water uptake or "effective" κ [67] will depend on both the droplet and dry particle sizes, due to the changing bulk–surface partitioning and droplet surface tension. [65,67,72,75] By including the surface tension corresponding to the droplet state where κ is evaluated, we here obtain a measure of the "intrinsic" κ value for NaDec at this state, representing specifically effects of water activity on droplet growth. For both RH = 99 % and 95 %, micelles are present in the droplets and the surface tension used for obtaining κ is constant σ = 22.5 mN m^{-1}, equal to the value at the CMC. For κ values derived at the critical supersaturation, droplet surface tensions vary between 45.0 − 54.9 mN m^{-1} for the MonoMic model and 43.5 − 51.5 mN m^{-1} for the CluMic model in the particle size range of D_p = 30 − 200 nm. Inverting κ values with the actual droplet surface tension means that this must be accounted for when calculating κ-Köhler curves using Eq. 46.

κ−values obtained at both supersaturated (Eq. 46) and subsaturated (Eq. 47) conditions are shown in Figs. S20B and S21. For each water activity model, κ values at a given humidity condition (RH = 99 % and 95 %, or S_c) increase with dry particle size and increase from subsaturated to supersaturated conditions at a given D_p. κ values for a given D_p obtained at subsaturated conditions decrease with RH, except for the MonoMic model at larger dry sizes. These trends reflect the varying solution state as the amount of solute in the droplet bulk (Figs. S23A,B and 24A,B) is governed by a complex balance between bulk−surface partitioning (Figs. S23D and 24D) and degree of micellization (Figs. S23C and S24C) as droplets grow and dilute. When micelles are present, κ values

tend to be larger for more concentrated droplets, even if the degree of micellization is higher, because the surface partitioning factor is simultaneously lower.

S1.3.6 Cloud droplet number concentration and climate radiative effect

We obtain a rough quantitative estimate of the change in climate radiative effect due to aerosol−cloud interactions when the formation of clusters or micelles within nascent cloud droplets is taken into account. The approach has been outlined in previous studies. [50,76,77] Only the radiative effects arising from changes in reflectivity of marine stratocumulus and stratus clouds are considered. These are the most prevailing clouds in the Earth system. [78] Due to negligible reflectance of the ocean surface, a change in cloud optical properties translates directly into a change in radiative effect. This method therefore provides an order of magnitude estimate of the total radiative effect.

Cloud droplet number concentration N for a given aerosol population is assumed to depend on the ambient supersaturation SS as $N \propto \mathrm{SS}^k$, where $k \approx 0.5$ [76]. Thus, assuming that N changes inversely with changing critical supersaturation $\mathrm{SS_c}$ due to change in activating droplet properties, the relative change in cloud droplet number concentration is estimated as [50]

$$\frac{\Delta N}{N_0} = -\frac{(\mathrm{SS_c})^k - (\mathrm{SS_c^0})^k}{(\mathrm{SS_c^0})^k} = \frac{(\mathrm{SS_c^0})^k - (\mathrm{SS_c})^k}{(\mathrm{SS_c^0})^k}, \tag{48}$$

where $\mathrm{SS_c^0}$ and N_0 are the critical supersaturation and cloud droplet number concentration for droplet activation in a given reference case. At constant atmospheric liquid water content, the change in cloud albedo A is

$$\Delta A = \frac{A(1-A)}{3}\frac{\Delta N}{N_0}, \tag{49}$$

and the change in aerosol–cloud radiative effect due to change in cloud albedo ΔA is now given as

$$\Delta RE = -Ff\tau_\mathrm{a}^2\Delta A, \tag{50}$$

where $F = 340$ W m^{-2} is the solar constant, $f = 0.3$ is the fractional coverage of marine stratus and stratocumulus clouds with albedo $A \approx 0.5$, and $\tau_a = 0.76$ is the transmittance of the atmosphere at the visible part of electromagnetic spectrum. [50]

The reference SS_c^0 values are obtained from κ-Köhler curves (Eq. 46) with κ values and droplet surface tensions obtained from Köhler model calculations and using the Mono water activity model at the critical supersaturation. These SS_c^0 closely resemble those of the original Köhler curves for the Mono model. The same constant surface tensions are used in all other κ-Köhler calculations for each dry particle size, where also the bulk−surface partitioning is the same. This ensures that the comparison only includes effects of micelles and/or clusters in the droplet bulk and does not reflect various surface effects related to bulk–surface partitioning of NaDec and droplet surface tension.

S2 Supplementary figures

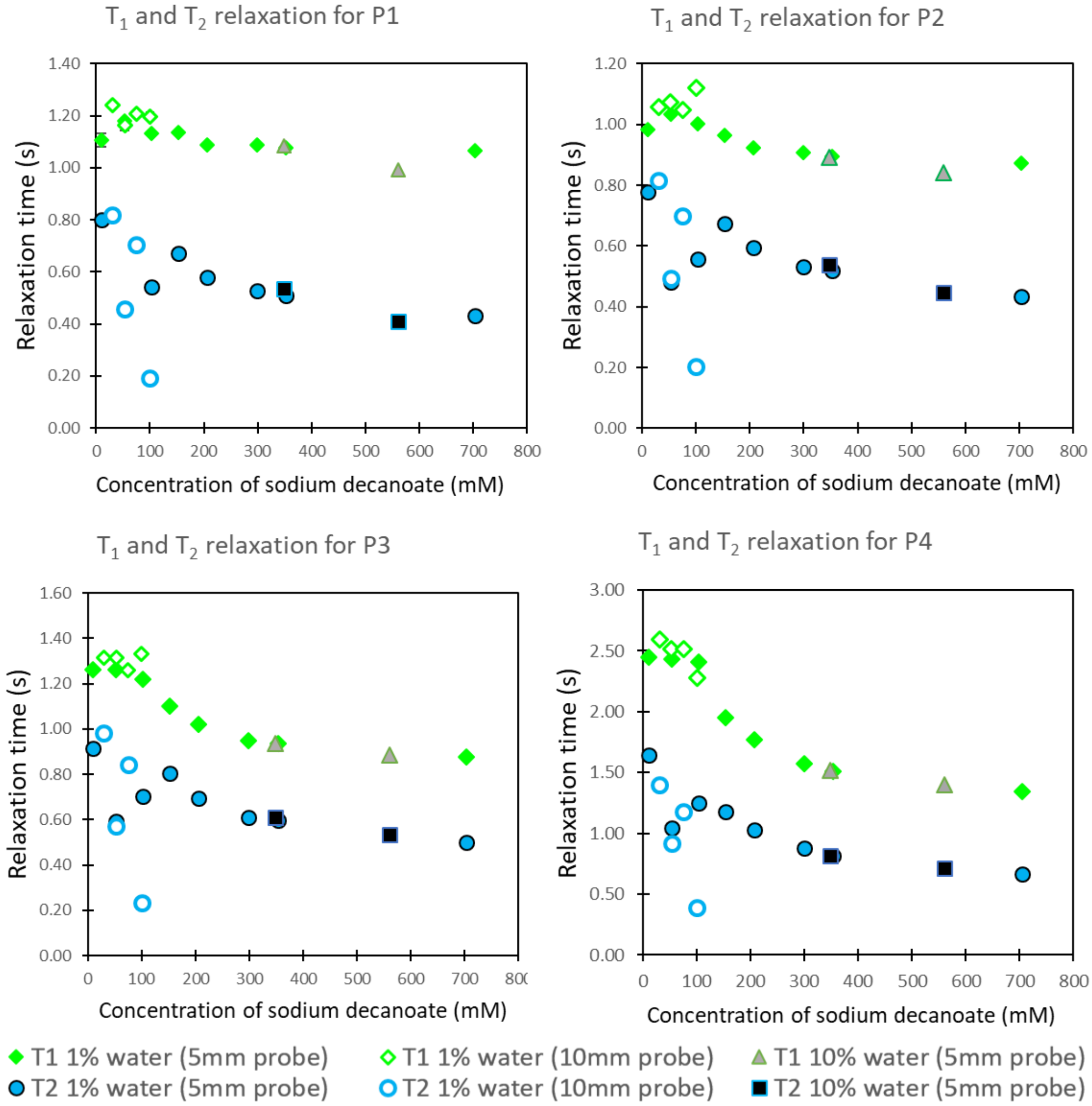

Figure S1. T_1 and T_2 relaxation times of decanoate as a function of concentration. Relaxation times are separately shown for peaks P1 – P4 indicated in Fig. 1A from the main text. The experiments were performed at 500 MHz field using either 1% or 10% of H_2O in D_2O.

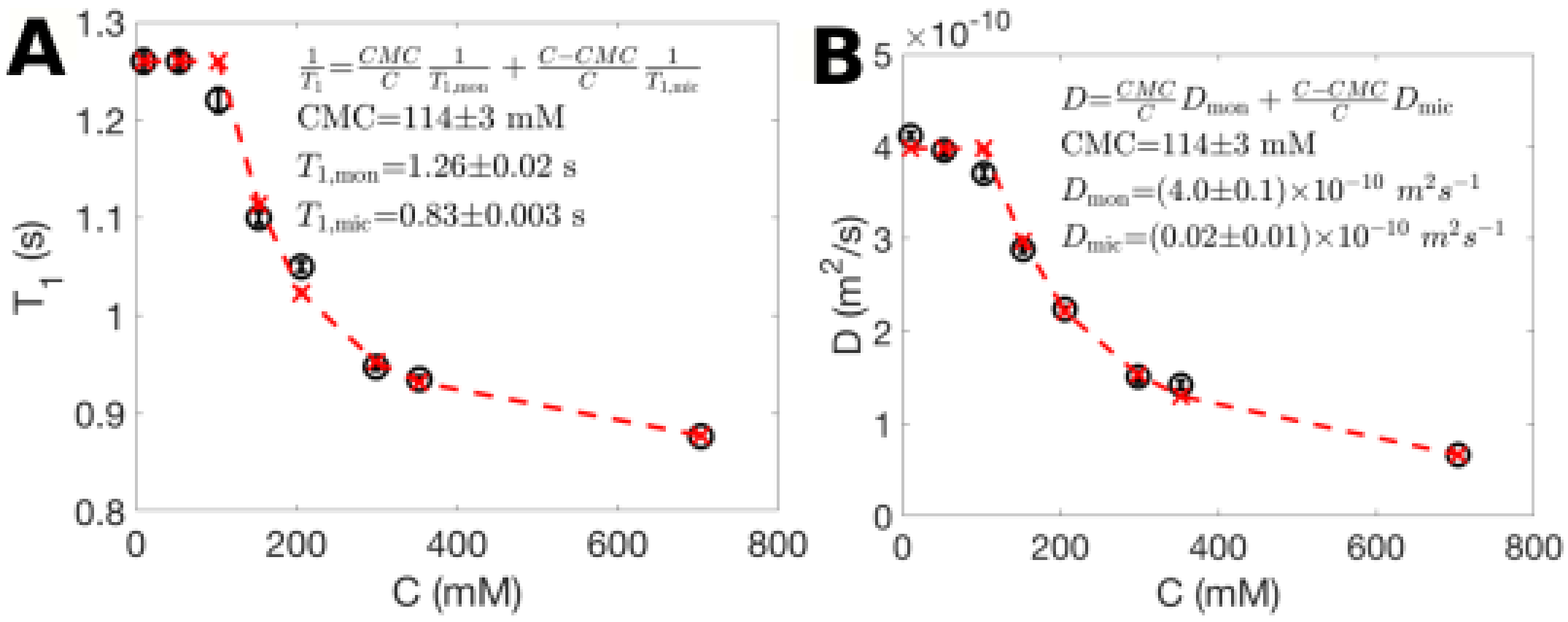

Figure S2. Fits of the conventional two site exchange model with the experimental NMR relaxation and diffusion data. (**A**) T_1 and (**B**) D data of decanoate peak P3 (black spheres). The fits are shown by red dashed lines and crosses. The conventional model assumes that, below the CMC, surfactants exist as monomers characterized by T_1 and D values of $T_{1,\text{mon}}$ and D_{mon}. Above the CMC, both monomers and micelles exist, and the concentration of monomers is constant (CMC). The number of micelles increases with increasing concentration, but the size of micelles is constant. T_1 and D of micelles are $T_{1,\text{mic}}$ and D_{mic}. The equations shown in figure describe the concentration dependence of observed T_1 and D above CMC. The global parameter estimation from T_1 and D (adjustable parameters: CMC, $T_{1\text{mono}}$, $T_{1\text{mic}}$, D_{mono} and D_{mic}) was done via the Monte Carlo procedure summarized in Methods leading to the cost function $\chi^2 = 44$ for the 16 data points.

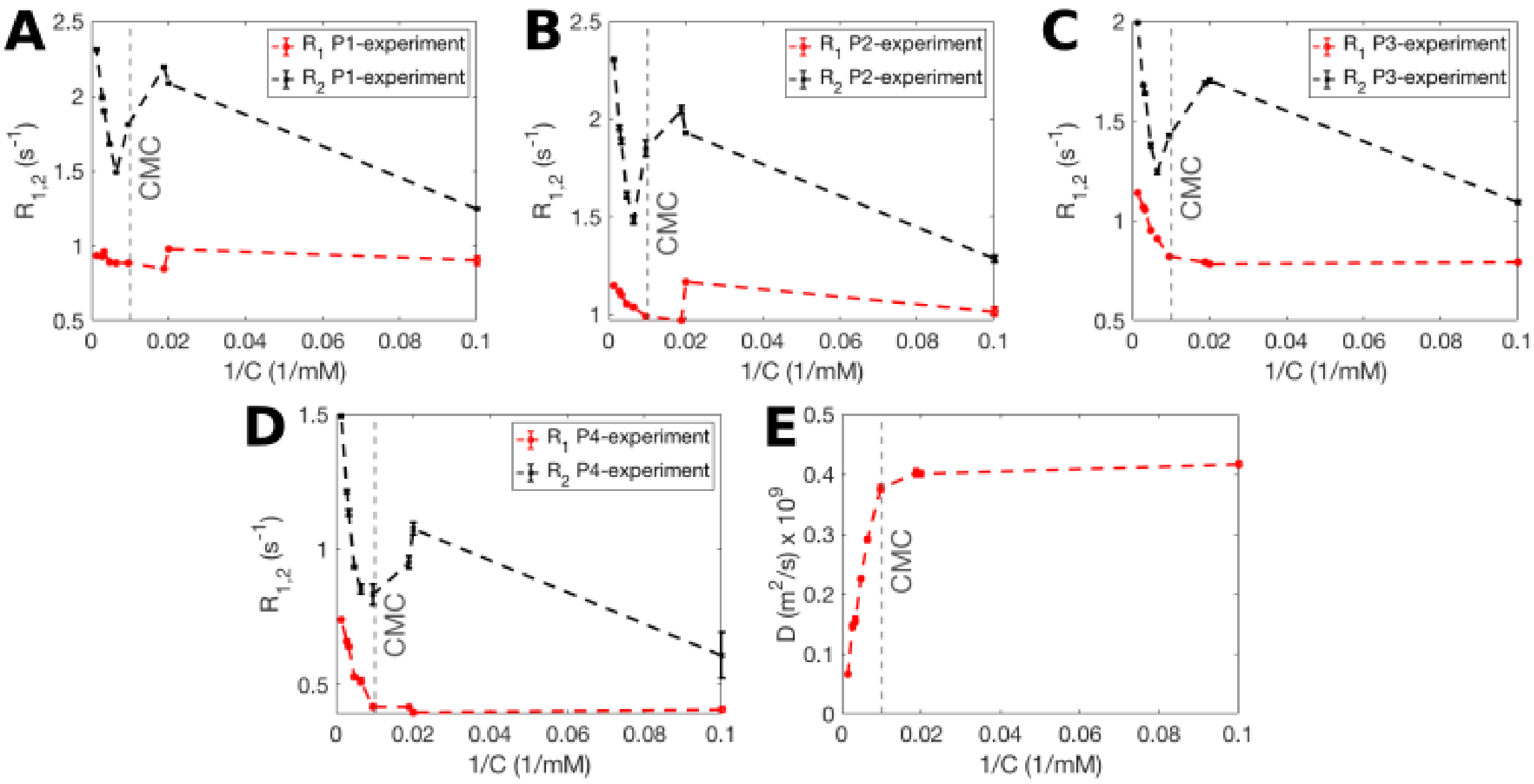

Figure S3. Relaxation rates and translational diffusion coefficients as a function of inverse of the surfactant concentration. (**A**)-(**D**) Relaxation rates of decanoate ^{1}H signals P1-P4. (**E**) Diffusion coefficient of decanoate. The critical micelle concentration (CMC) is indicated by gray vertical dotted lines. The experiments were performed at the 500 MHz field.

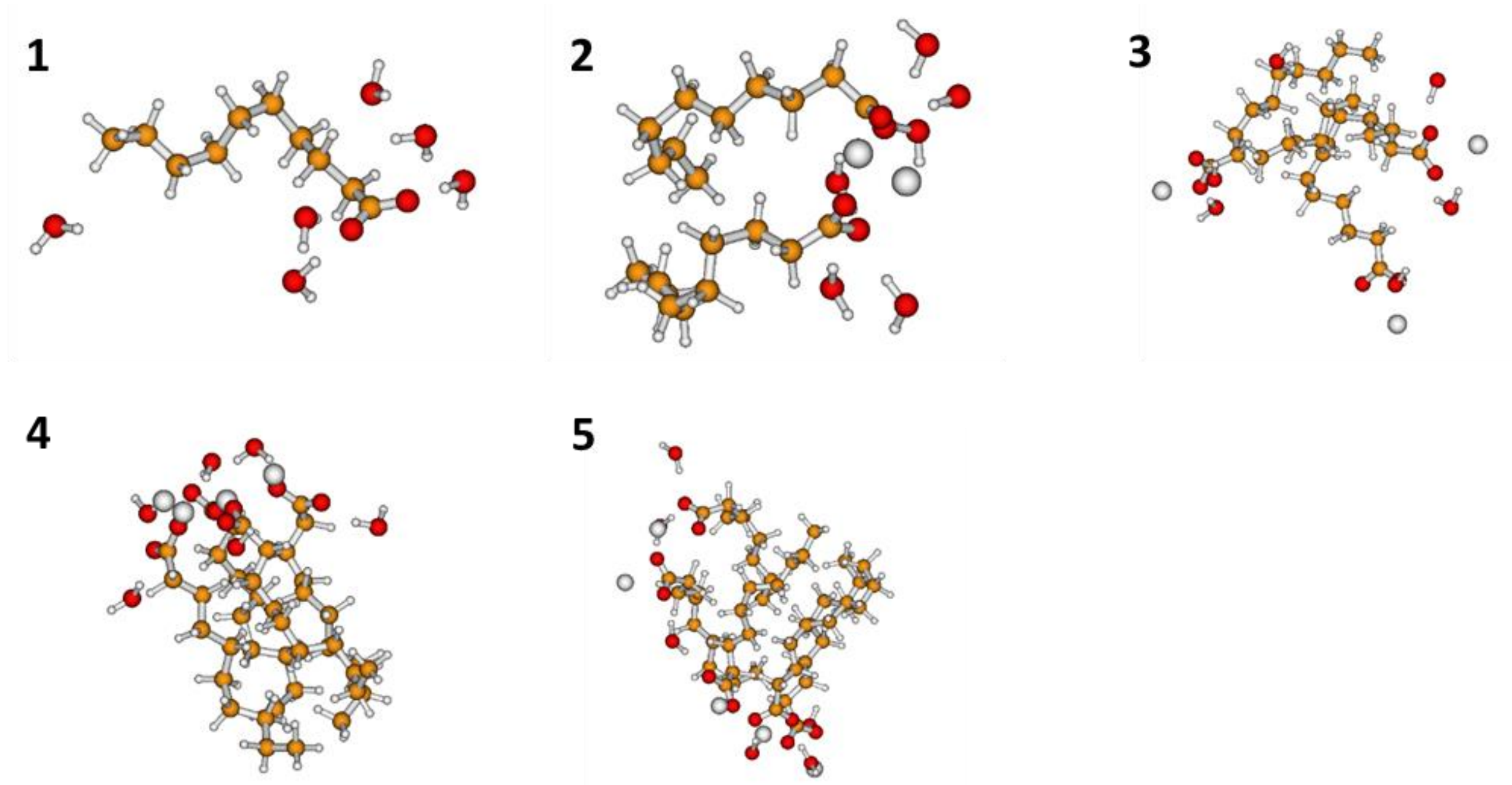

Figure S4. Snapshots of monomer to pentamer surfactant clusters MD (QM/MM) simulations.

The snapshots include six water molecules and sodium ions (grey).

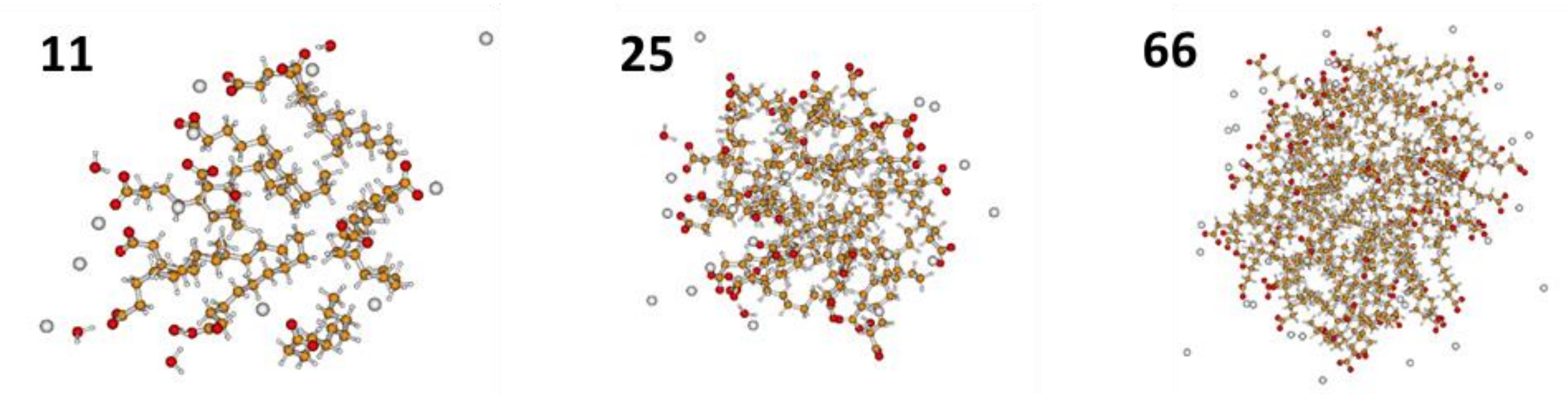

Figure S5. Snapshots of all classical MM simulations of the micelle type clusters including 11, 25 and 66 surfactants. The simulations include six water molecules and sodium ions (grey).

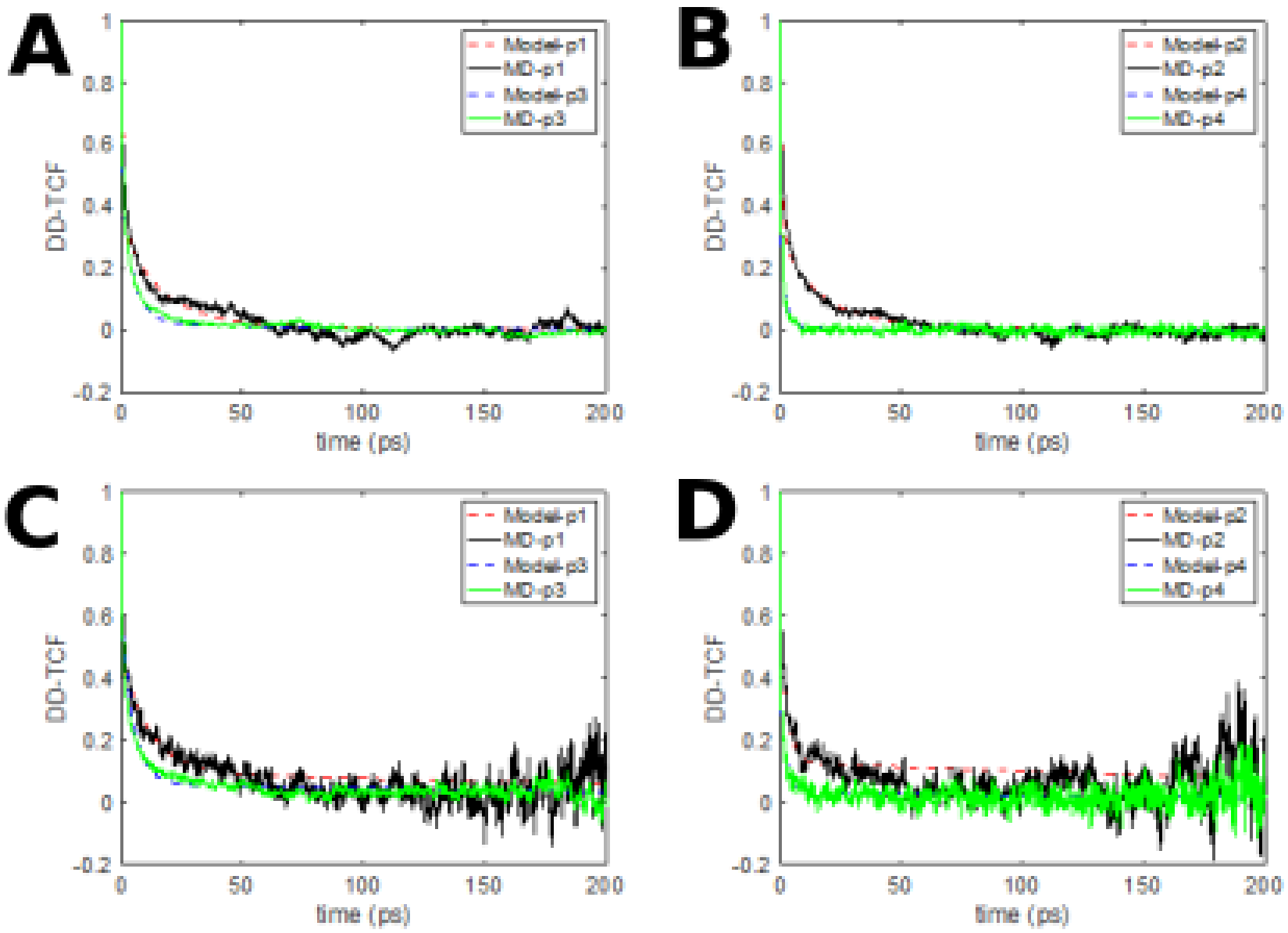

Figure S6. Time correlation functions (TCFs) of clusters. (**A**) and (**B**) Dipole-dipole TCF (Eq. S5) from MD simulation together with fits of two-step model (Eq. 6) of monomer. (**C**) and (**D**) corresponding results for pentamer. Labels p1-p4 indicate traces for individual protons sites indicated in Fig. 1A from the main text.

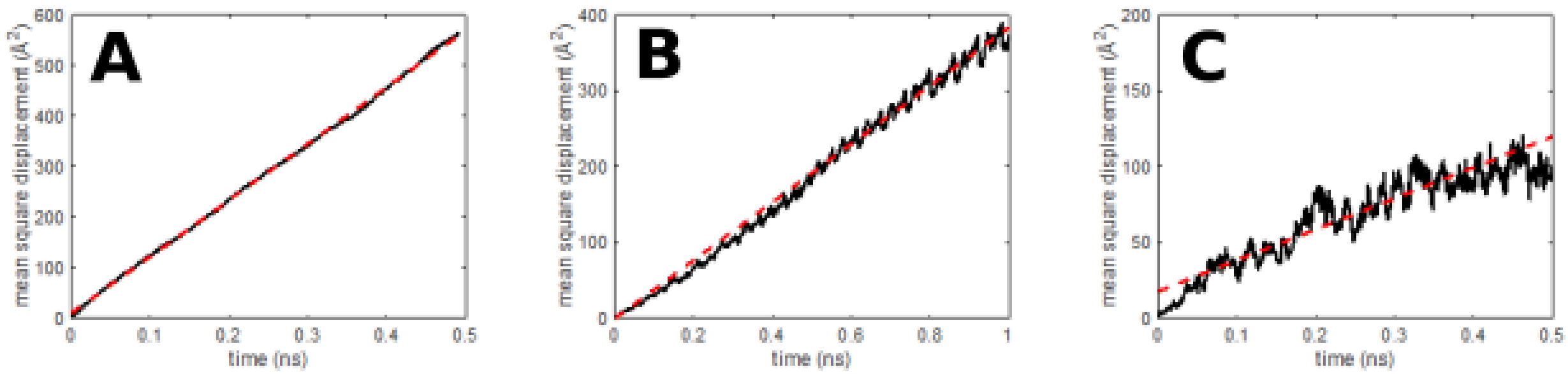

Figure S7. MD simulated mean square displacements. (**A**) Water, (**B**) monomer (protons P1) and (**C**) pentamer (protons P1). Linear fits with the simulated data are shown by dashed red lines. The fits were used to calculate the diffusion coefficient of water and monomer by using Eq. 9. Diffusion coefficient of pentamer was calculated based on $[\tilde{R}_H\ \text{(monomer)}/\tilde{R}_H\ \text{(multimer)}]$ ratio and Eq. 8.

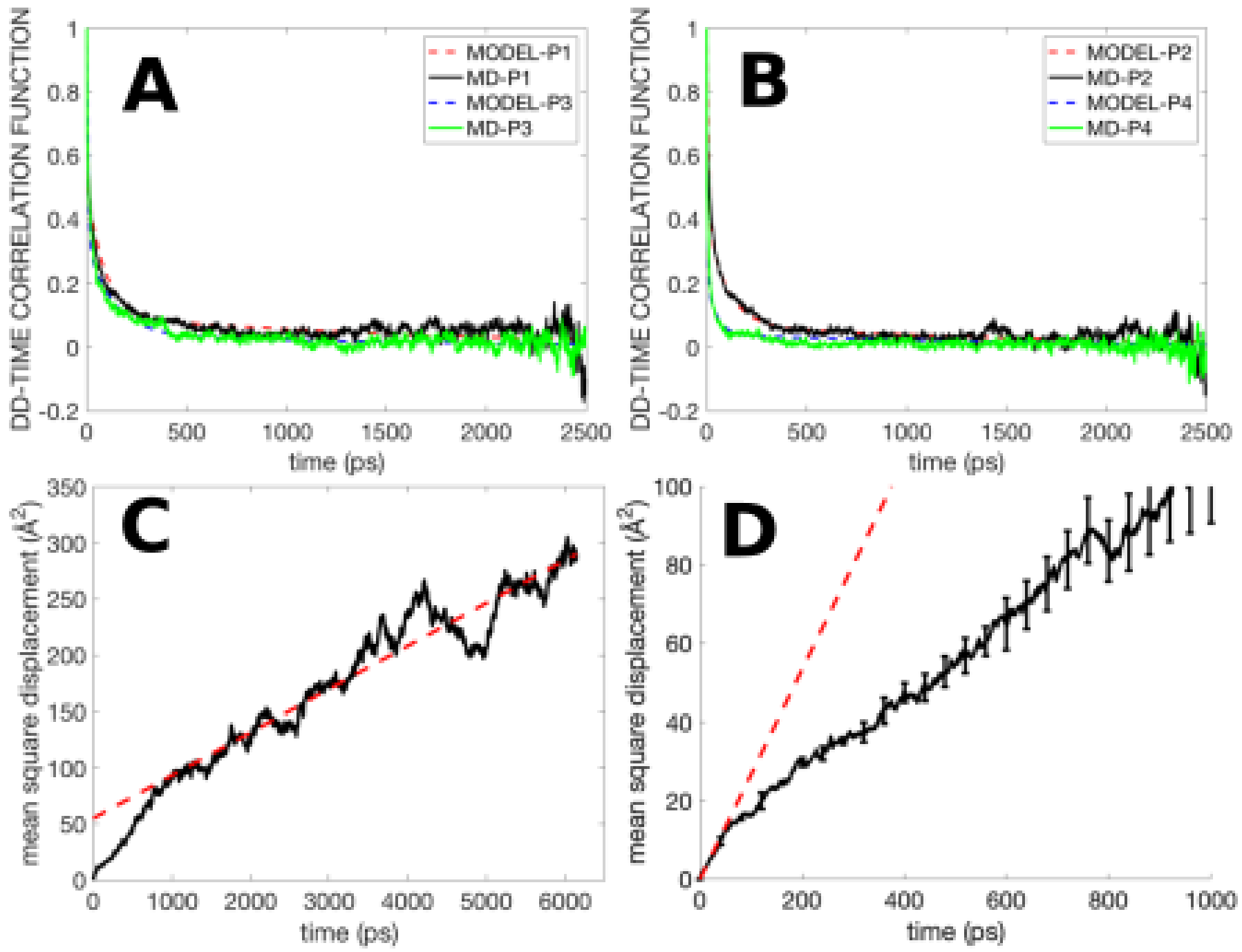

Figure S8. Time-correlation functions and mean square displacements of the micelle. (**A**) and (**B**) MD simulated dipole-dipole time correlation functions (Eq. 5) of the 66-surfactant micelle (solid lines) along with the fits of the two-step model (Eq. 6) with the data (dashed lines). (**C**) MD simulated mean square surfactant displacement, in the limit of long times representing micelle displacement, along with a linear fit with the data. (**D**) mean square headgroup carbon displacement with the center of mass of micelle fixed, approximating surface displacement at short time.

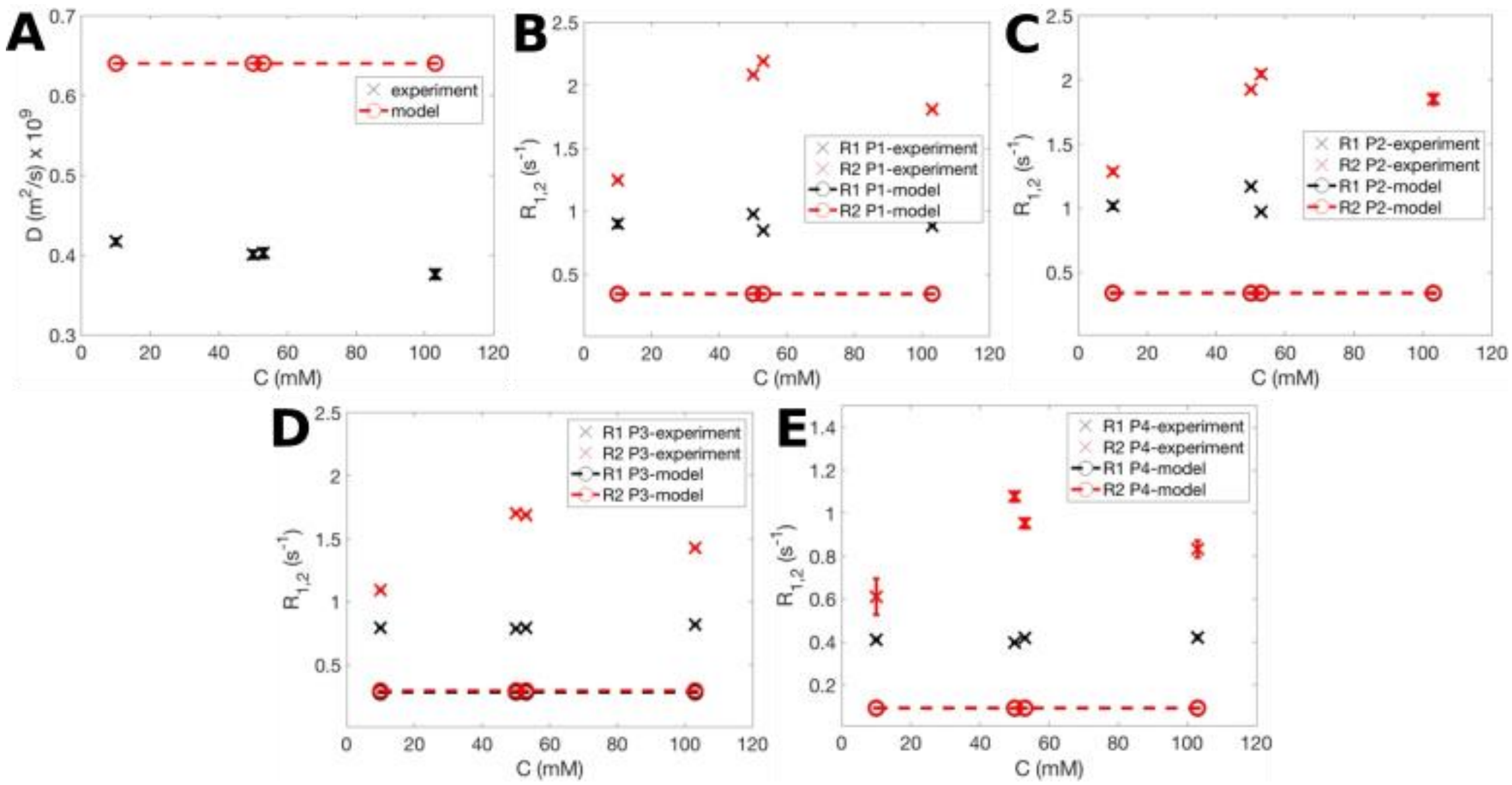

Figure S9. Fits of the monomer model with the experimental relaxation and diffusion data below the CMC. (**A**) Diffusion coefficients. (**B**)-(**E**) Relaxation rates R_1 and R_2 for peaks P1-P4. The simulation takes into account the intra- and inter-molecular dipole-dipole mechanisms. The model does not include any adjustable parameters. The sum of the square cost-function $\chi^2 = 2.1 \cdot 10^6$. The resonance frequency is 500 MHz.

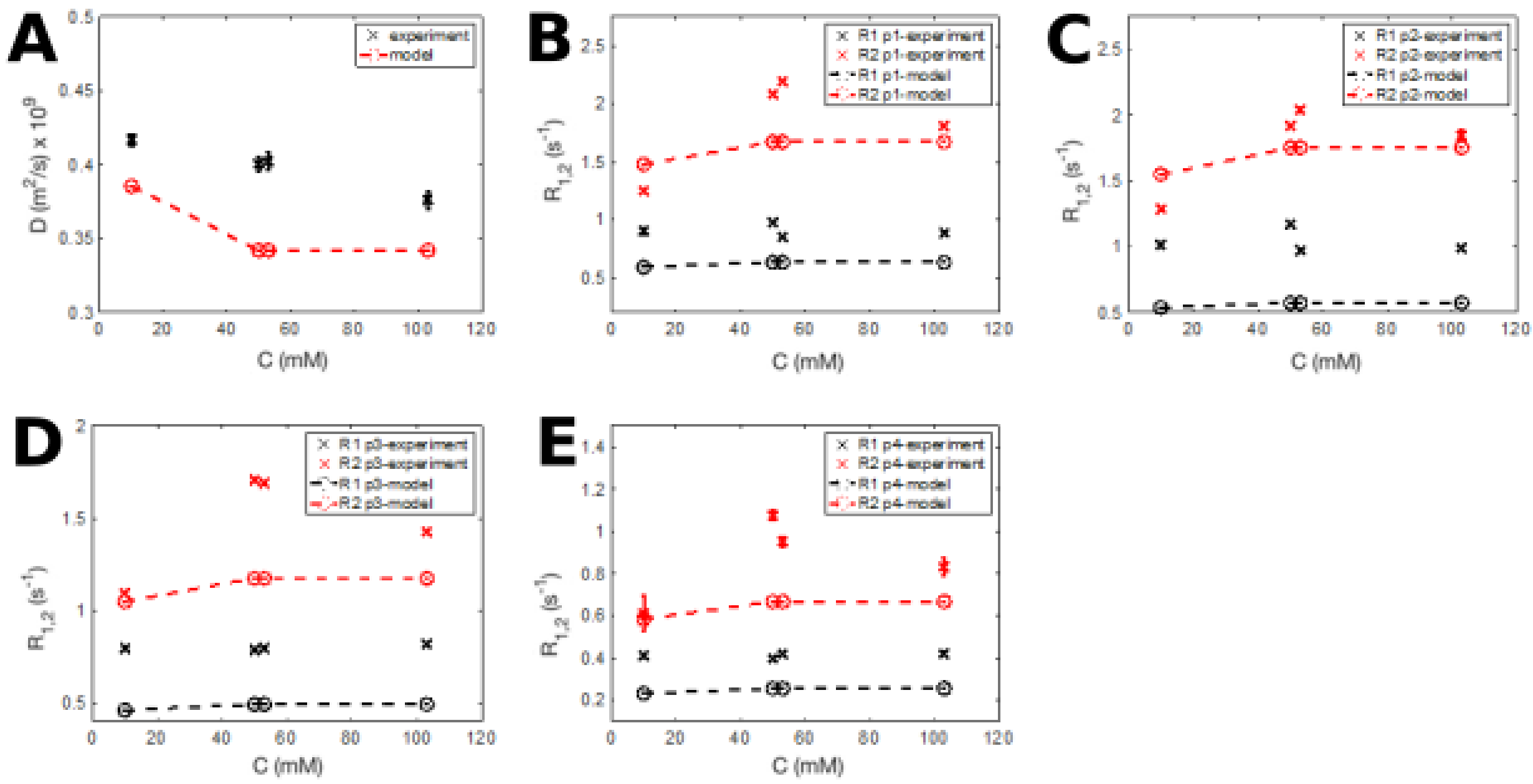

Figure S10. Fits of the monomer-pentamer two site model with the experimental relaxation and diffusion data. (**A**) Diffusion coefficients. (**B**)-(**E**) Relaxation rates R_1 and R_2 for peaks P1-P4. The model includes intra- and intermolecular dipole-dipole relaxation mechanisms. The fitted pentamer fractions at C = 10, 50 and 103 mM are X_A = 0.77, 1, and 1, *i.e.*, the monomer fraction X_B = 1- X_A is zero at the higher concentrations (but below the CMC). The sum of the square cost-function χ^2 = $7.5{\cdot}10^4$.

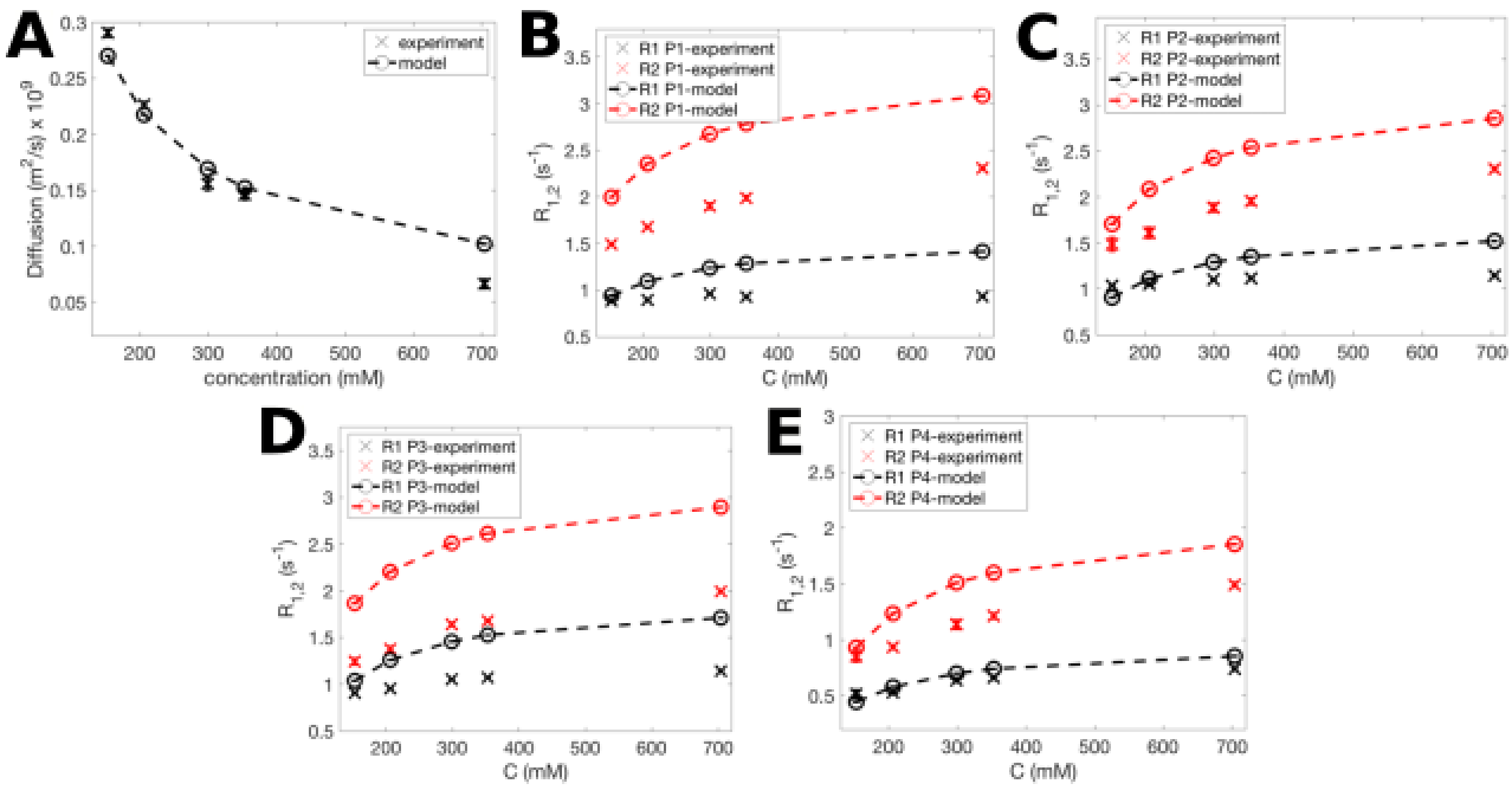

Figure S11. Global fit of Model 1 with the diffusion and relaxation data above the CMC. The model is based on the MD-simulation with a fixed small size cluster (pentamer) and a large 66-surfactant micelle. The sum of the square cost-function $\chi^2 = 2.3 \cdot 10^5$. The adjustable parameters are in Table S4.

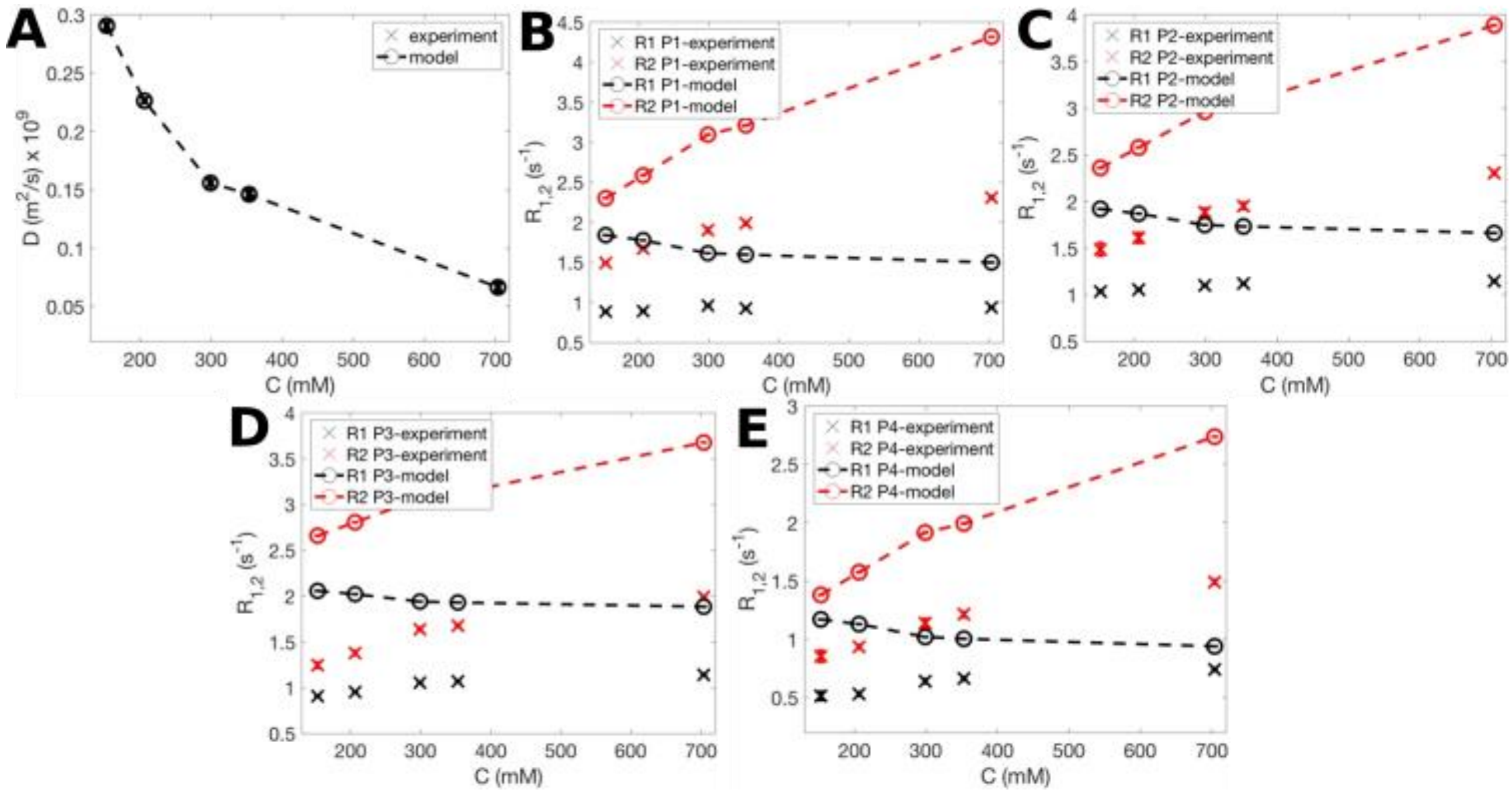

Figure S12. Global fit of Model 2 with the diffusion and relaxation data above the CMC. In the model assumes that only micelles exist above the CMC, without any monomers or smaller clusters. The radius of the micelle was calculated by the Stokes-Einstein equation using the experimentally observed diffusion coefficient. Fast dynamics was predicted with MD-simulation of 66-surfactant micelle. The sum of the square cost-function $\chi^2 = 5.9 \cdot 10^5$. The adjustable parameters are in Table S5.

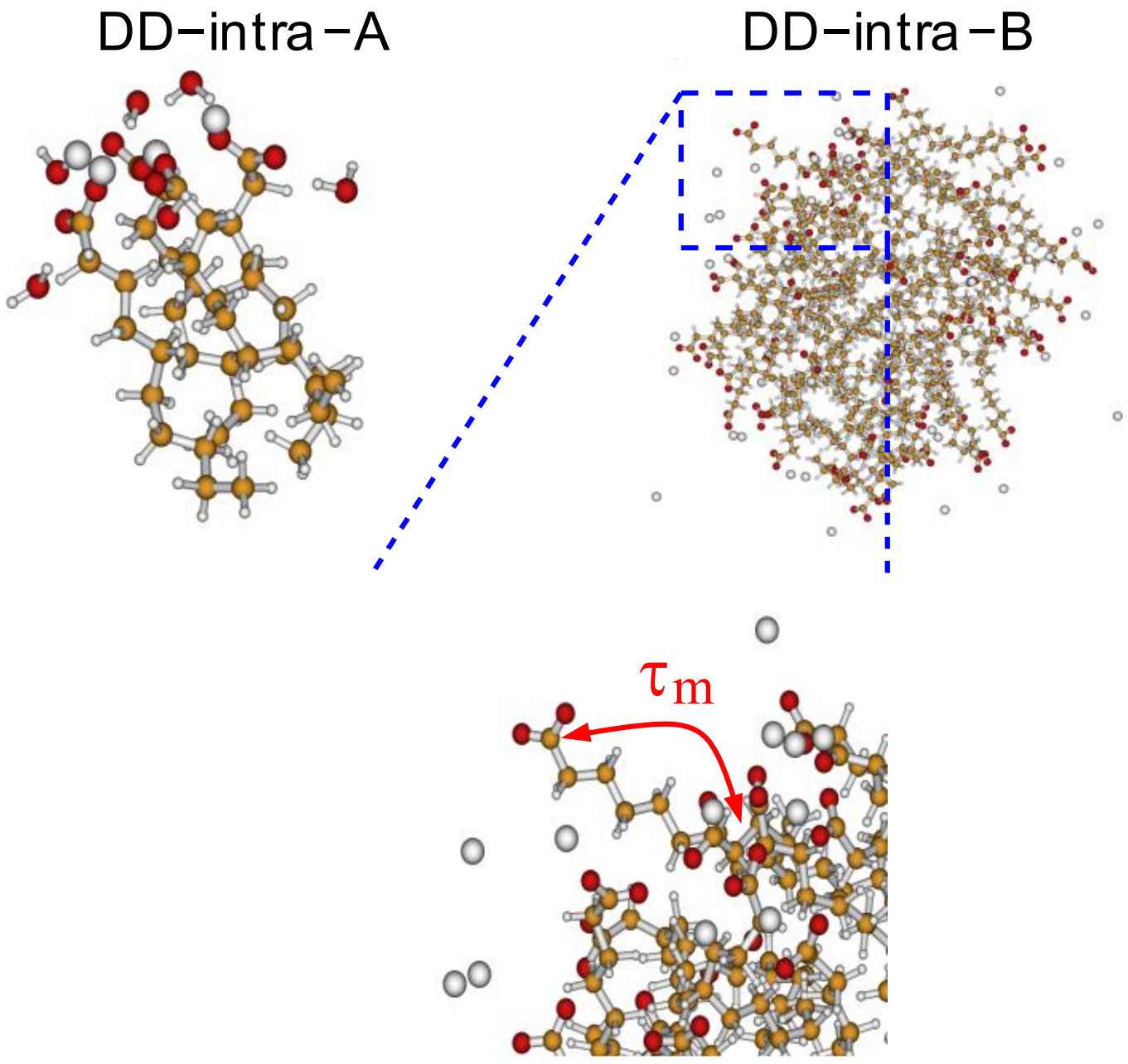

Figure S13. Illustration of the molecular protrusion taken into account in Model 3. The figure includes a small cluster A and a large micelle B. The protrusion takes place on the surface of the micelle and is characterized by the residence time τ_{m}.

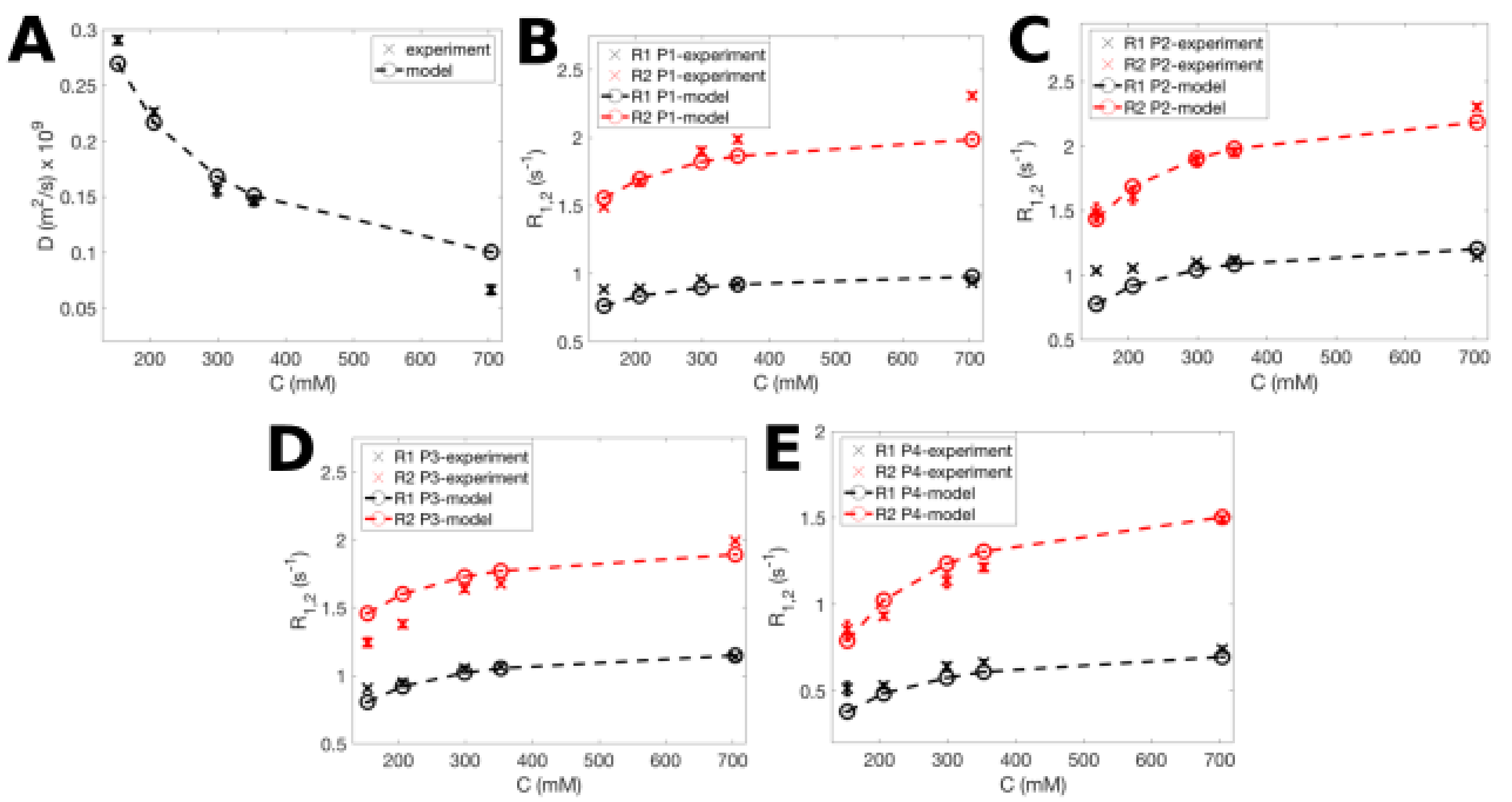

Figure S14. Global fit of Model 3 with the diffusion and relaxation data above the CMC. The model is based on the MD-simulation with a fixed small size cluster (pentamer) and a large 66-surfactant micelle, similarly to Model 1. However, it takes into account molecular protrusion as well. The sum of the square cost-function $\chi^2 = 2.5 \cdot 10^3$. The adjustable parameters are in Table S6.

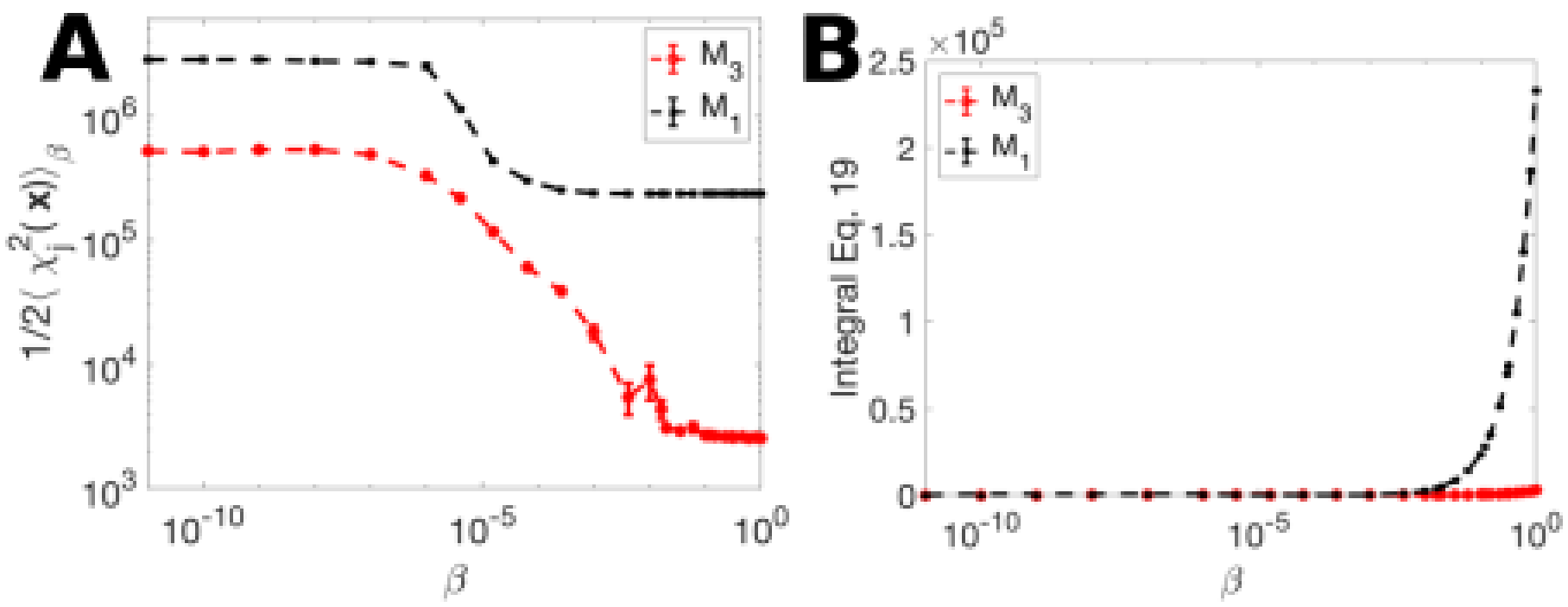

Figure S15. (A) the integrand and (B) cumulative integral of Eq. 19 computed with MCMC for Model 1 (black) and 3 (red).

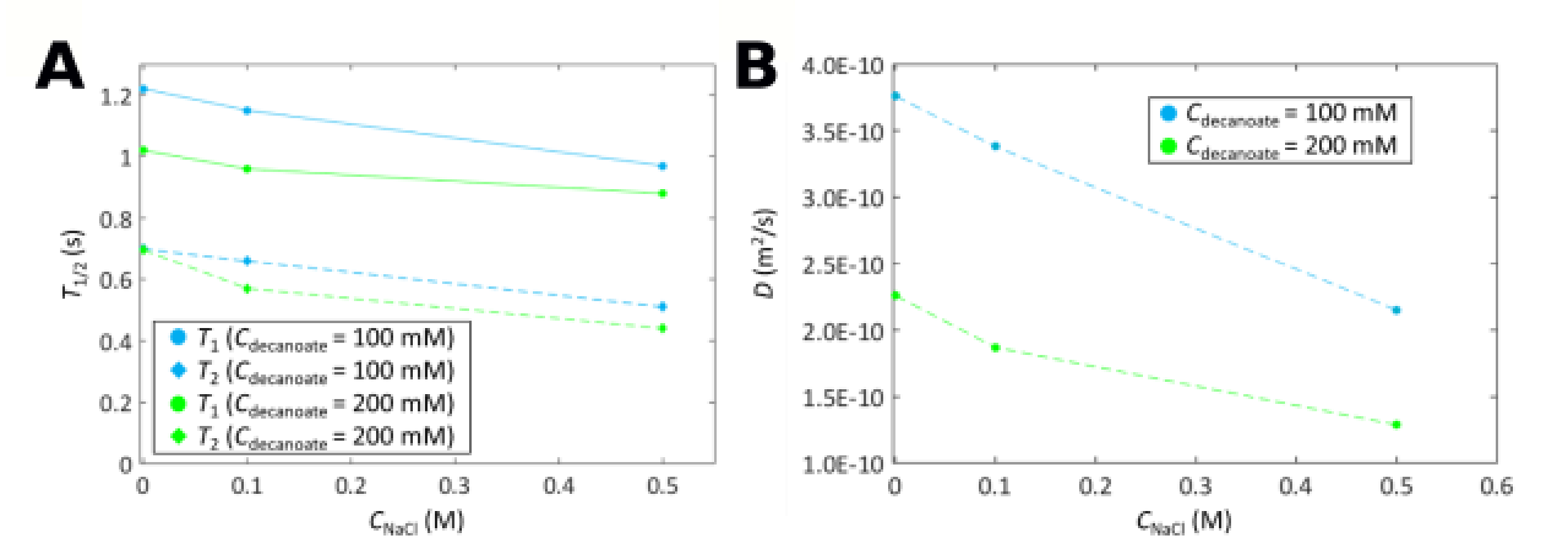

Figure S16. Effect of salt (NaCl) on the relaxation times and diffusion coefficient of decanoate. (**A**) T_1 and T_2 relaxation times of the methylene peak P3 as a function of NaCl concentration, measured at two different decanoate concentrations (100 and 200 mM). When the salt concentration increases from 0 to 0.5 M, T_1 and T_2 decrease about 20% and 30%, respectively. (**B**) Diffusion coefficient of decanoate as a function of NaCl concentration, measured at two different decanoate concentrations (100 and 200 mM). D decreases about 40% when the salt concentration increases from 0 to 0.5 M. Overall, the relaxation and diffusion measurements indicate that the dynamics of the surfactant cluster/micelle system is slowing down with increasing salt concentration. However, detailed modelling of these effects is out of the scope of the current work.

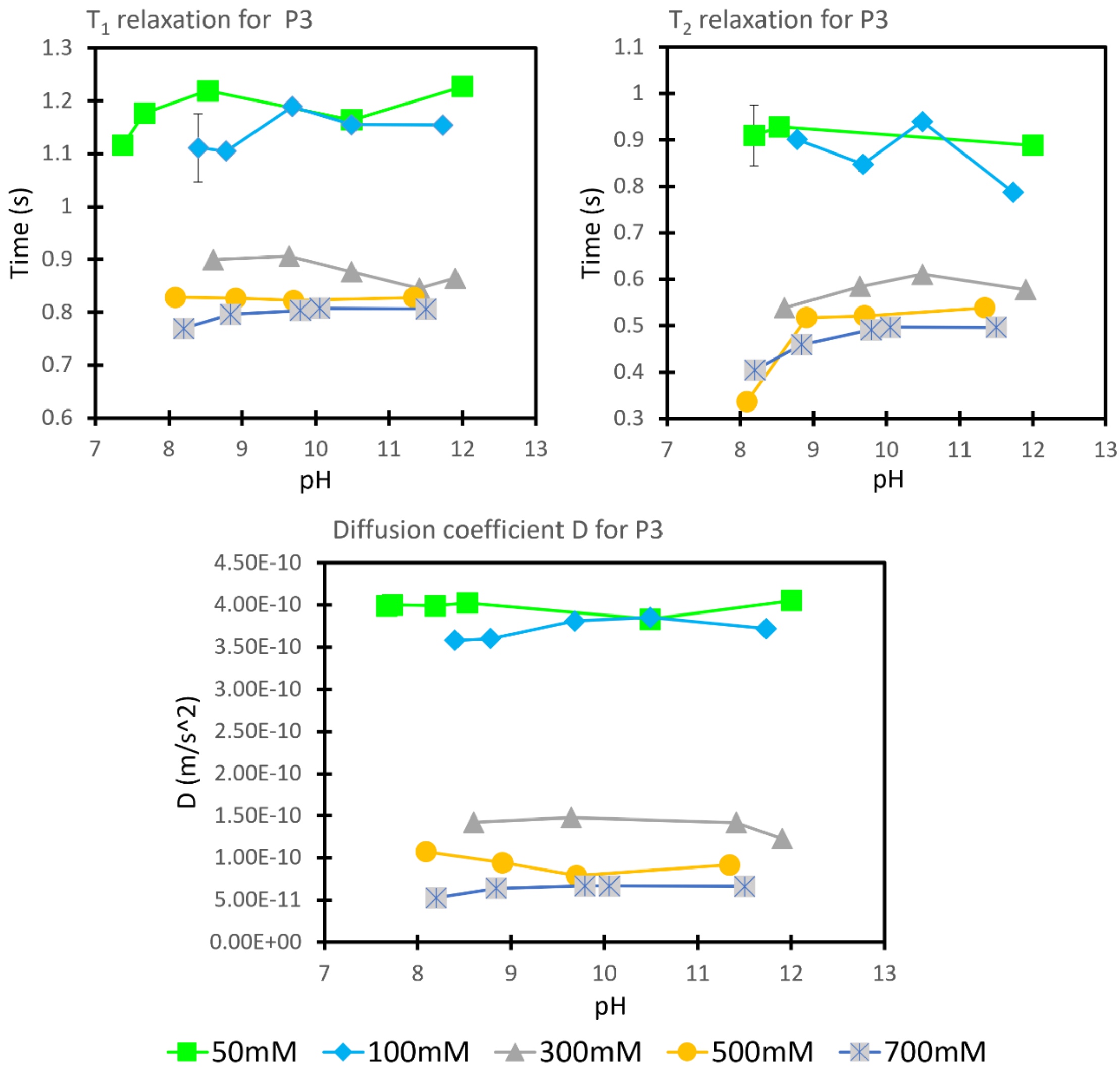

Figure S17. T_1 and T_2 relaxation times and diffusion coefficient D of sodium decanoate as a function of pH measured with 400 MHz spectrometer. The relaxation times and diffusion coefficients do not change much as a function of pH. This implies that CMC does not change significantly in the pH range of 7.5-12.

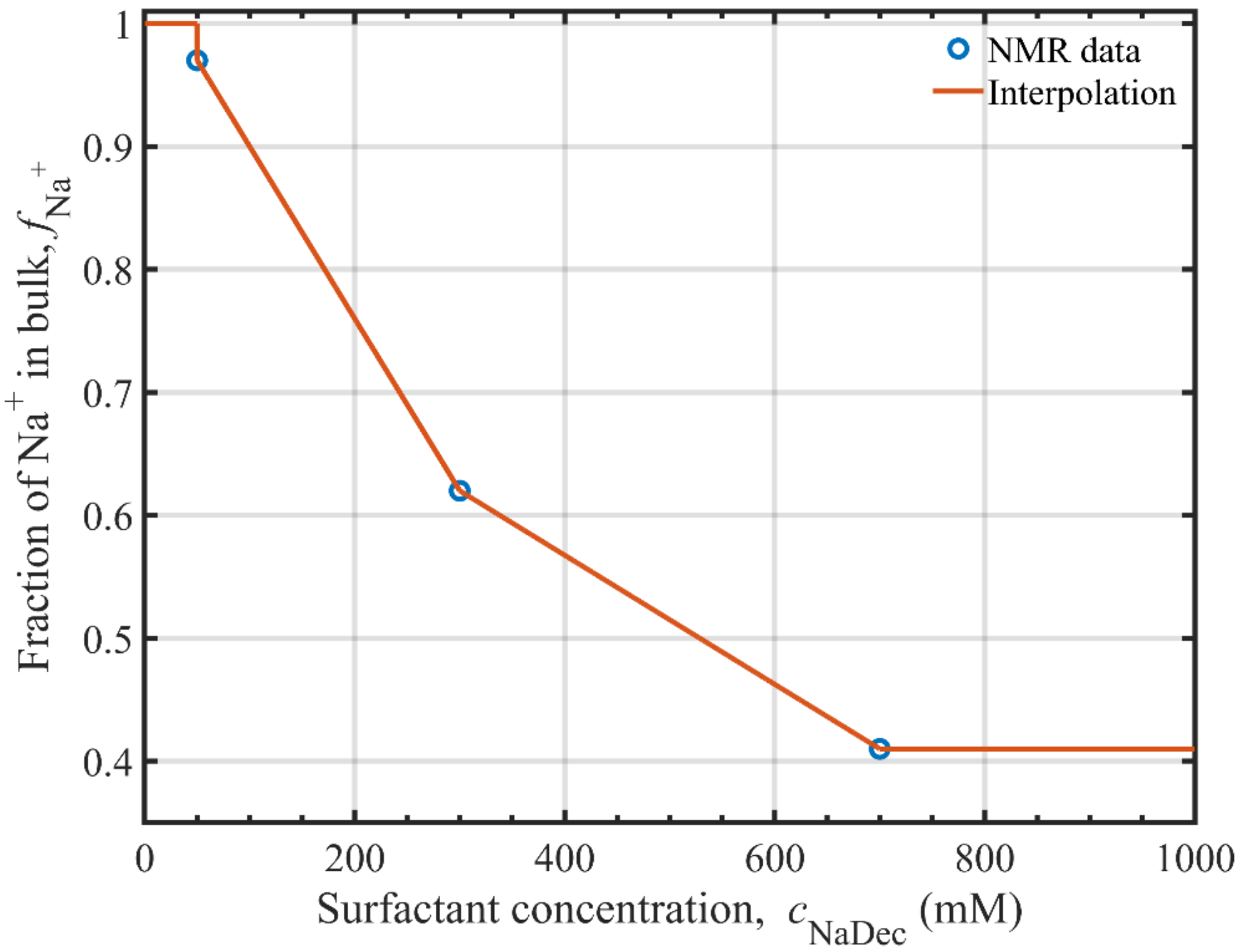

Figure S18. Fraction of free sodium ions in droplet bulk. The fraction of Na^+ ions $\boldsymbol{f}_{Na^+}$ was evaluated by ^{23}Na NMR relaxation measurements and associated quadrupolar relaxation modelling.[54] Solid line indicates the interpolation and extrapolation used in the CluMicNa model. Numerical values are shown in Table S8.

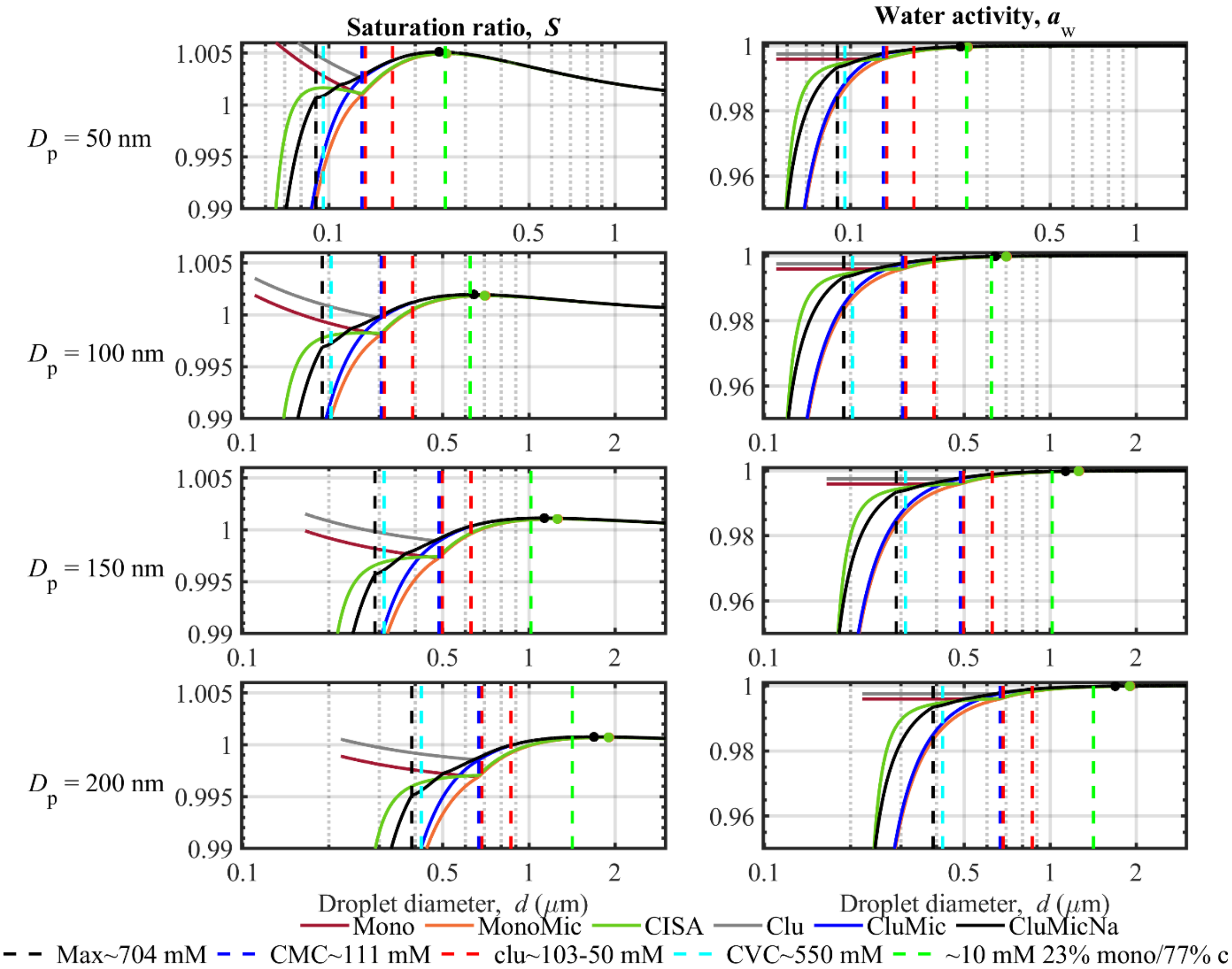

Figure S19. Cloud droplet formation. Köhler growth curves (*d, S*) for spherical sodium decanoate particles with diameters D_p = 50, 100, 150, and 200 nm, calculated from Eq. 20 with different water activity models (left) and the corresponding droplet water activity a_w along the Köhler curves (right). The droplet sizes marked as dashed vertical lines correspond to various NaDec concentrations from the NMR experiments: the largest concentration measured (704 mM), the CVC (550 mM), the region where all monomers are thought to form clusters (103−50 mM) and a concentration where 23% are thought to remain as monomers and the rest form small clusters (10 mM), as well as the CMC (111 mM) calculated with Eq. 43. The marked concentrations are calculated from the bulk phase composition of the droplets for the MonoMic, CISA, CluMic, and CluMicNa models.

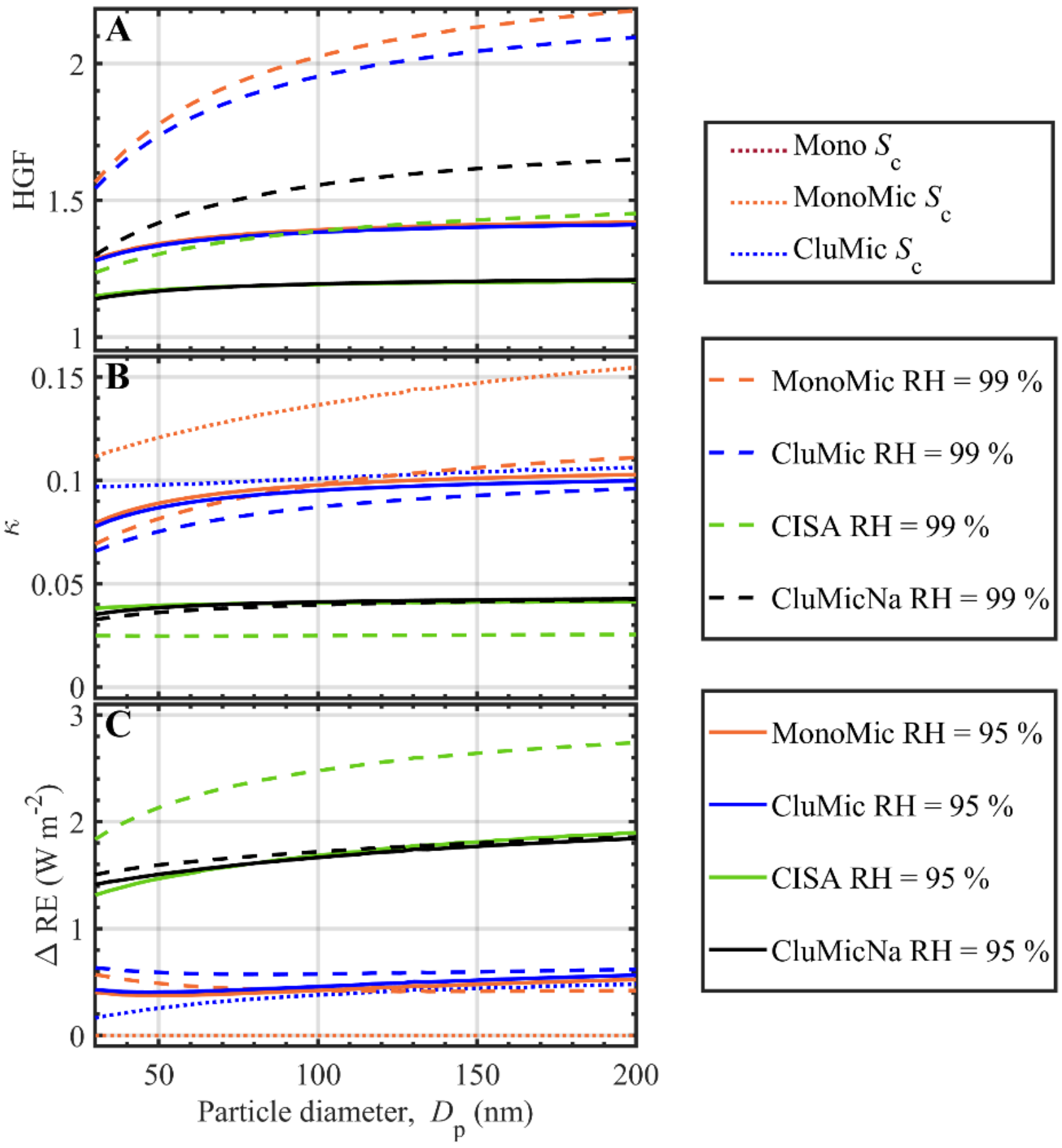

Figure S20. Particle water uptake and climate radiative forcing. (**A**) Hygroscopic growth factors (HGF) for spherical sodium decanoate particles at ambient relative humidity RH = 95% and RH = 99 % calculated for a range of dry particle sizes D_p = 30−200 nm using the predictions of Eq. 20 for the MonoMic, CluMic, CluMicNa, and CISA water activity models. (**B**) Hygroscopicity parameter (κ) values for the same sodium decanoate particles, calculated by fitting Eq. 46 to the predictions of S_c from Eq. 20 with the MonoMic and CluMic models, and with Eq. 47 at RH = 95 % and RH = 99 % from the predictions of the MonoMic, CluMic, CluMicNa, and CISA models, in each case taking the droplet surface tension into account. (**C**) Estimated change in aerosol–cloud−climate radiative effect (ΔRE) from the reference case (predictions using the κ-values obtained from the Mono model at the critical supersaturations for each size) when considering formation of clusters and micelles in the droplets, as a function of the smallest activating particle diameter (D_p). κ-Köhler calculations of S_c for determining ΔRE (Eq. 48) are made by extrapolating hygroscopic properties predicted with the

different water activity models (κ values in panel b) at the critical supersaturation and sub-saturated conditions using Eq. 46. The same surface tension as for the reference case is used for all κ-Köhler predictions, to assess the impact of micelles, clusters, and counter-ion binding on changing the droplet water activity without confounding influences of surface tension.

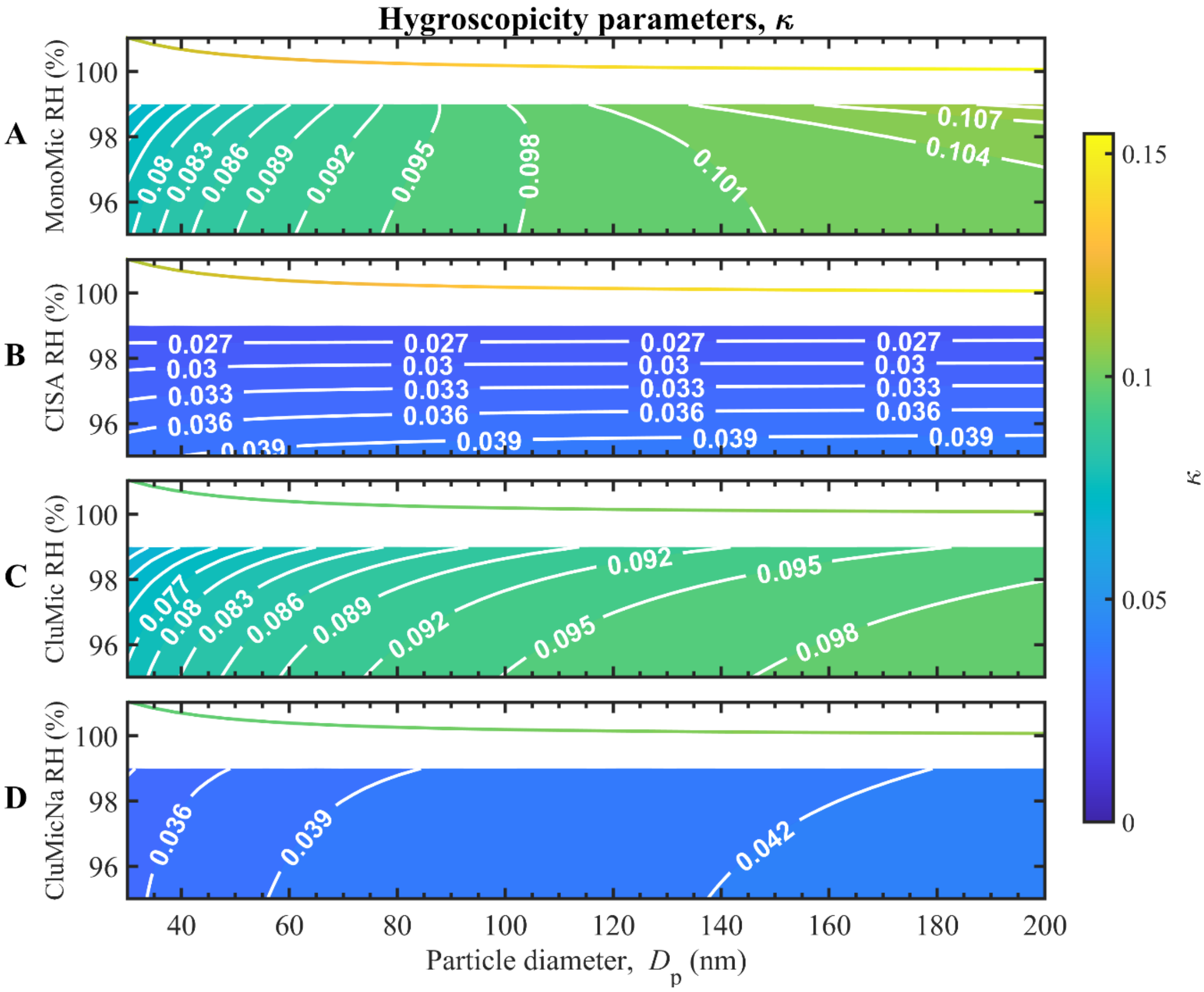

Figure S21. Hygroscopicity parameters. Ranges of the hygroscopicity parameter (κ) calculated for sodium decanoate particles at the critical supersaturation, by fitting Eq. 46 to the predictions of Eq. 20 (colored line), and at subsaturated relative humidities RH = 95−99 %, calculated with Eq. 47 (contour plots), each from the predictions of the MonoMic (**A**), CISA (**B**), CluMic (**C**), and CluMicNa (**D**), water activity models for particles with dry diameters D_p = 30−200 nm.

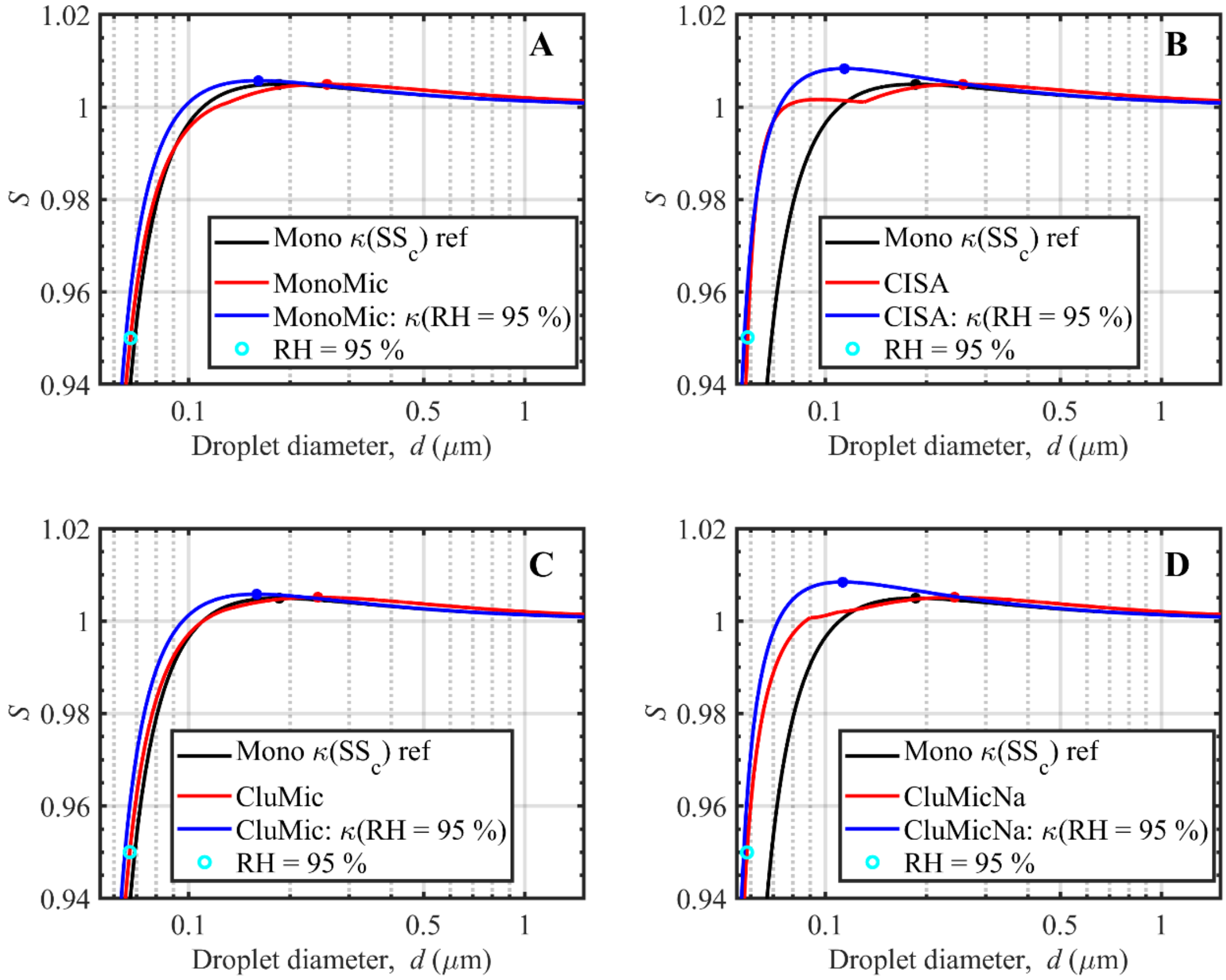

Figure S22. Examples of κ-Köhler curves. The κ-Köhler curves (Eq. 46) calculated for sodium decanoate particles with dry sizes $D_{\mathrm{p}} = 50$ nm using κ-values evaluated at RH = 95 % (Eq. 47, Fig. S20B) and surface tension equal to the Mono model predictions at the critical point are shown in blue for the (**A**) MonoMic, (**B**) CISA, (**C**) CluMic, and (**D**) CluMicNa models. The corresponding original Köhler curves (Eq. 20) for each of the water activity models are shown in red. The κ-Köhler curve with κ and σ obtained from the Mono model at the critical supersaturation (the reference case for ΔRE) is shown in black in each panel.

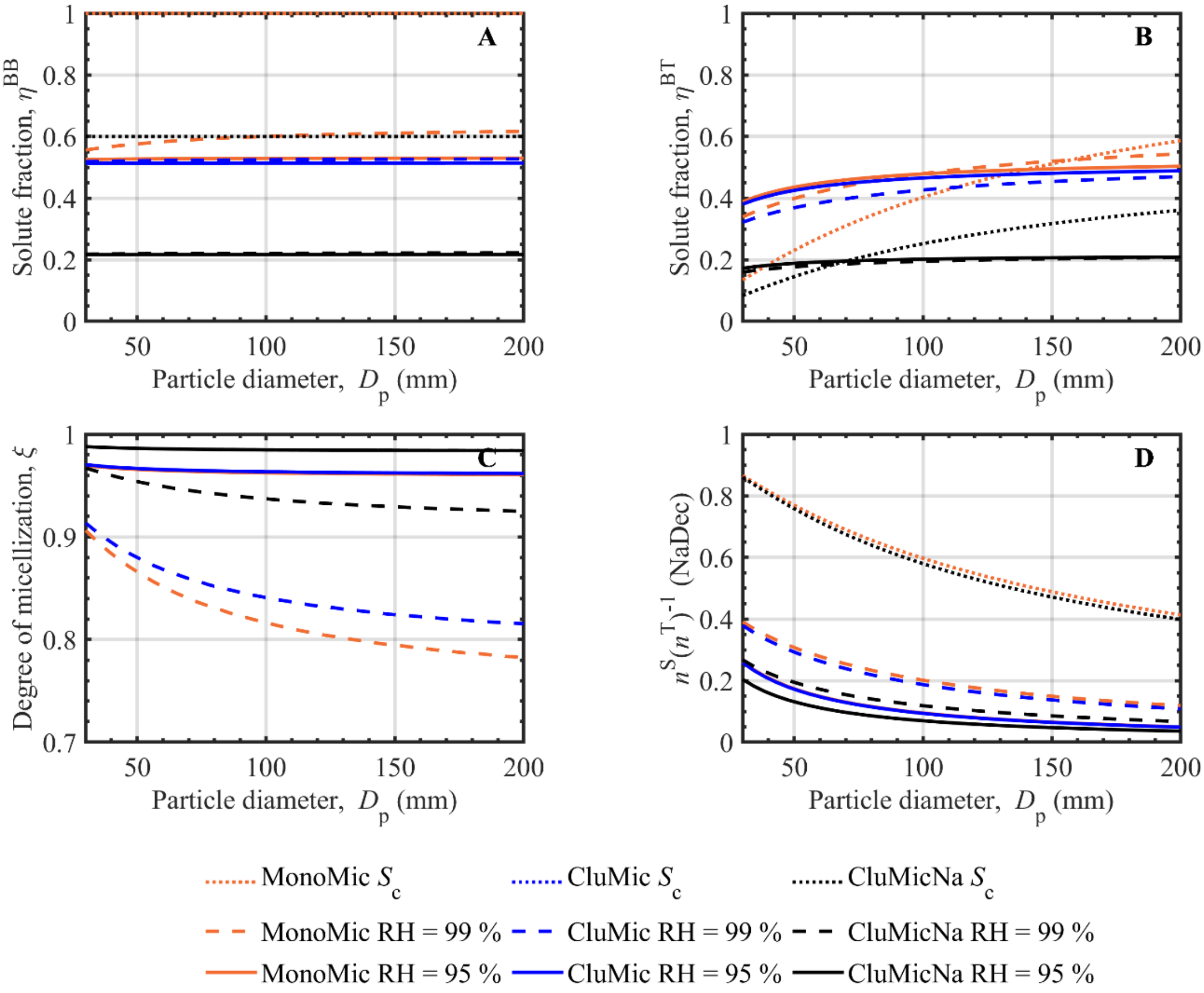

Figure S23. Droplet solution state for different humidity conditions and dry particle sizes. For the MonoMic, CluMic, and CluMicNa models is shown: (**A**) The solute fraction η^{BB}, given as the amount of solute entities (sum of monomers or clusters, micelles, and free Na^{+} ions) in the droplet bulk phase divided by the maximum amount of possible solute entities in the droplet bulk (amount of fully dissociated NaDec monomers left in the droplet bulk after accounting for bulk-surface partitioning). (**B**) The solute fraction η^{BT}, given as the amount of solute entities in the droplet bulk phase divided by the maximum amount of possible solute entities in the whole droplet (amount of fully dissociated NaDec monomers based on the total amount of NaDec in both the droplet bulk and surface phases). (**C**) The degree of micellization (Eq. 44), describing the fraction of NaDec monomers in the bulk forming micelles, after accounting for bulk−surface partitioning. (**D**) Surface partitioning factor for undissociated NaDec, calculated as the amount of NaDec monomers in the surface phase

relative to the total amount of NaDec monomers in the whole droplet. The solute fractions $\eta^{\mathrm{BT}}$and η^{BB}show the complex dependencies of droplet state on bulk–surface partitioning and micellization as droplets grow and become more dilute. Droplets formed on larger particles have smaller surface-to-bulk ratios, resulting in smaller surface partitioning factors (D) and in turn higher bulk concentrations and lower surface tensions, at a given HGF. This leads to higher HGF for larger D_{p} (Fig. S20A), lower bulk concentrations, and lower degree of micellization (C), at a given humidity condition. The bulk solute fraction η^{BT} with respect to the maximum amount of solute entities for the whole droplet increases with D_{p} (B), reflecting both these effects, whereas the fraction η^{BB} with respect to the maximum amount of solute entities for the droplet bulk changes relatively little (A).

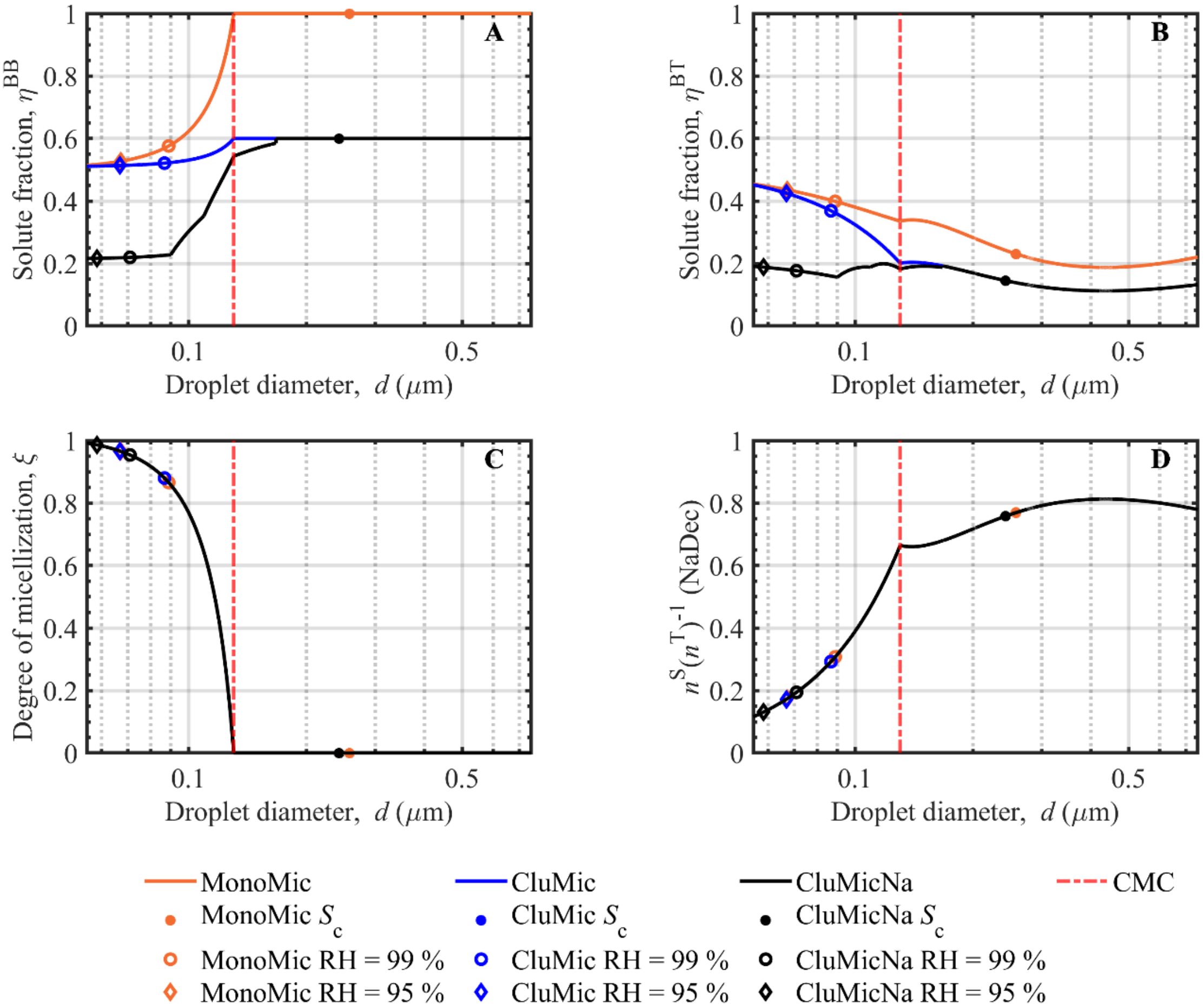

Figure S24. Solution state in growing droplets. Examples of the parameters from Fig. S23. for growing droplets, corresponding to increasing humidity conditions, formed on dry sodium decanoate particles with $D_p = 50$ nm. (**A**) The solute fraction η^{BB} with respect to the maximum amount of solute entities in the bulk. (**B**) The solute fraction η^{BT} with respect to the maximum amount of solute entities in the whole droplet. (**C**) The degree of micellization (Eq. 44). (**D**) The surface partitioning factor of undissociated NaDec monomers. As the droplet grows and dilutes, total surface area increases and a larger fraction of the fixed amount of solute partitions to the surface (D). Both dilution and bulk−surface partitioning lead to a decreased degree of micellization (C), which increases the solute fraction in the bulk (A), but the bulk solute fraction with respect to the total (B) decreases due to the stronger effect of increased bulk−surface partitioning.

S3 Supplementary tables

Table S1. Parameters in the cluster simulations. The number of surfactants in the cluster is given in the first row, and the second row indicates the simulation method. The third row shows the number of molecules in the water solvent (water forces were computed with the classical MM method). The timespan of pressure equilibrium at 1 bar is given in the fourth row. The two last rows indicate the final production times and the simulation box sizes (averages over x, y and z). In addition, five 67-surfactant simulations with 11521 waters where one is 160 ns, to explore surfactant residence time, and four are 20 ns, study of micelle water pocket.

#Surfactant	1	2	3	4	5	11	25	67	67
Force calc.	QM	QM	QM	QM	QM	MM	MM	MM	MM
#Water	1900	8400	10700	4800	5700	3709	5068	8601	11521
Equil. (ns)	0.3	0.9	1	1	1	3	3	6	6
Prod. (ns)	2	7.4	1	0.6	2.4	2	1.2	5	4×20, 160
Box (Å)	38	55	68	50	55	48	54	65	72

Table S2. The parameters resulting from the lipid DD-TCF MD simulations (Eq. 5). The clusters were simulated with the PM3 QM Hamiltonian. The weights in Eq. 4 are $s_i = \{a_1(1-S^2), a_2(1-S^2), S^2\}$, where $a_2 = 1 - a_1$.

proton#	τ_1 (ps)	τ_2 (ps)	a_1	a_2	S^2	r_{eff} (Å)
**Monomer** [$\tau_R = 76$ ps; $R_H = 3.35$ Å; $D_{tr} = 6.4 \cdot 10^{-10}$ m^{2}s^{-1}]						
P1	0.7	3.8	0.60	0.40	0.042	1.66
P2	0.8	20	0.72	0.28	0.04	1.63
P3	0.33	5.8	0.53	0.47	0.03	1.60
P4	0.22	2.60	0.70	0.30	0.01	1.52
**Dimer** [$\tau_R = 212$ ps; $R_H = 4.73$ Å; $D_{tr} = 4.5 \cdot 10^{-10}$m^{2}s^{-1}]						
P1	0.47	6.7	0.50	0.40	0.11	1.64
P2	0.48	6.5	0.61	0.39	0.04	1.60
P3	0.55	4.8	0.72	0.28	0.01	1.58
P4	0.13	1.1	0.59	0.41	0.005	1.51
**Trimer** [$\tau_R = 273$ ps; $R_H = 5.15$ Å; $D_{tr} = 4.2 \cdot 10^{-10}$m^{2}s^{-1}]						
P1	0.16	14.0	0.65	0.35	0.05	1.57
P2	0.45	12.9	0.76	0.24	0.02	1.58
P3	0.29	7.9	0.73	0.27	0.025	1.53
P4	0.09	3.8	0.80	0.20	0.01	1.49
**Tetramer** [$\tau_R = 371$ ps; $R_H = 5.7$ Å; $D_{tr} = 3.8 \cdot 10^{-10}$m^{2}s^{-1}]						
P1	0.06	3.0	0.62	0.38	0.115	1.63
P2	0.09	5.0	0.65	0.35	0.062	1.56
P3	0.06	2.4	0.58	0.42	0.074	1.53
P4	0.08	3.1	0.85	0.15	0.015	1.49

**Pentamer** [$\tau_R = 498$ ps; $R_H = 6.3$ Å; $D_{tr} = 3.4 \cdot 10^{-10} m^2 s^{-1}$]						
P1	0.5	13	0.54	0.46	0.09	1.59
P2	0.2	3.1	0.4	0.6	0.12	1.56
P3	0.004	6.0	0.40	0.60	0.06	1.52
P4	0.20	3.6	0.70	0.30	0.03	1.47

Table S3. The parameters resulting from DD-TCF MD simulations (Eq. 5) of the micelles including 11, 25 and 66 surfactants. The weights in Eq. 4 are $s_i = \{a_1(1 - S^2), a_2(1 - S^2), S^2\}$, where $a_2 = 1 - a_1$.

**Micelle (11 surfactants)** [τ_{eff} = 460 ps; $R_H = 16 \pm 1$ Å]						
proton#	τ_1 (ps)	τ_2 (ps)	a_1	a_2	S^2	r_{eff} (Å)
P1	2.0	59	0.46	0.54	0.035	1.63
P2	0.6	34	0.34	0.66	0.085	1.62
P3	0.6	28	0.37	0.63	0.075	1.58
P4	0.5	19	0.52	0.48	0.02	1.51
**Micelle (25 surfactants)** [τ_{eff} = 650 ps; $R_H = 19 \pm 1$ Å]						
P1	1.2	54	0.5	0.5	0.105	1.63
P2	1.1	45	0.45	0.55	0.10	1.60
P3	0.25	25	0.37	0.63	0.14	1.57
P4	2.1	30	0.69	0.31	0.025	1.49
**Micelle (66 surfactants)** [τ_{eff} = 1800 ps; $R_H = 30 \pm 2$ Å]						
P1	2.3	87	0.54	0.46	0.1	1.63
P2	9.2	98	0.63	0.37	0.07	1.61
P3	7.0	132	0.70	0.30	0.04	1.57
P4	3.5	47	0.82	0.18	0.04	1.50

Table S4. The parameters resulting from the global fit of Model 1 with the diffusion and relaxation data above the CMC. The model is based on the MD-simulation with a fixed small size cluster (pentamer) and a large 66-surfactant micelle. The micelle radius r_{eff} and the intramolecular DD relaxation rate were adjustable parameters. The sum of the square cost-function $\chi^2 = 2.3 \cdot 10^5$. First row: mean of five MCMC trajectories, second row: 95% confidence interval and third row parameter prior boundaries. From parameterization $\tau_D = 1.8$ ns and $\tau_R = 21$ ns.

	P1, r_{eff} (Å)	P2, r_{eff} (Å)	P3, r_{eff} (Å)	P4, r_{eff} (Å)	R (Å)
Estimate(x):	1.67	1.64	1.60	1.52	26.64
95%-conf:	upper bound	upper bound	upper bound	upper bound	[26.5, 26.7]
Boundary	[1.61, 1.67]	[1.59, 1.64]	[1.54, 1.60]	[1.47, 1.52]	[10, 100]

Table S5. The parameters resulting from the global fit of Model 2 with the diffusion and relaxation data above the CMC. The micelle radii extracted from the diffusion data are 6.3, 8, 11, 12 and 23 Å at 153, 206, 299, 353 and 704 mM. Fast dynamics was predicted with MD-simulation of 66-surfactant micelle. The sum of the square cost-function $\chi^2 = 5.9 \cdot 10^5$. The fits resulted in the effective distance (r_{eff}), and the fitted effective correlation time (τ_{eff}).

	P1, r_{eff} (Å)	P2, r_{eff} (Å)	P3, r_{eff} (Å)	P4, r_{eff} (Å)	τ_{eff} (ns)
Estimate(x):	1.67	1.64	1.60	1.52	2.332
95%-conf:	upper bound	upper bound	upper bound	upper bound	[2.33,2.34]
Boundary	[1.61, 1.67]	[1.59, 1.64]	[1.54, 1.60]	[1.47, 1.52]	[1, 400]

Table S6. The parameters resulting from the global fit of Model 3 with the diffusion and relaxation data above the CMC. The model is based on the MD-simulation with a fixed small size cluster (pentamer) and a large 66-surfactant micelle. From determined radius, the correlation times τ_R and τ_D are 21 and 1.8 ns, respectively. The sum of the square cost-function $\chi^2 = 2.5 \cdot 10^3$.

	P1, *f*	P2, *f*	P3, *f*	P4, *f*	*R* (Å)	τ_m (ns)
Estimate(*x*):	0.51	0.64	0.527	0.706	27.4	44
95%-conf:	[0.5, 0.515]	[0.63, 0.644]	[0.52, 0.53]	[0.69, 0.72]	[26.6, 30.1]	[5.7, 99]
Boundary	[0, 1]	[0, 1]	[0, 1]	[0, 1]	[10 100]	[0.1,100]

Table S7. The Bayesian evidence computed with MCMC and numerical integration of Eq. 19.

Model	-ln[P($EXP|M_j$)]	χ^2_{opt}
1	232630±0.2	$233 \cdot 10^3$
2	587300±0.1	$587 \cdot 10^3$
3	2740±9	$2.5 \cdot 10^3$

Table S8. Fraction of free sodium ions in droplet bulk. The fraction was evaluated by ^{23}Na NMR relaxation measurements and associated quadrupolar relaxation modelling. [54]

NaDec concentration (mM)	$\boldsymbol{f}_{\mathbf{Na^+}}$,
**50**	0.97
**300**	0.62
**700**	0.41

Table S9. CISA model parameters used to calculate activity coefficients in aqueous solutions of sodium decanoate. [55] M, X and Mic here designate Na^+, Dec^- and micelles, respectively. Parameter $\beta_{\mathrm{ca}}^{(0)}$ and $\beta_{\mathrm{ca}}^{(1)}$ values for Eq. 33 are listed in the first and second row, respectively.

Solute	$\boldsymbol{\beta}_{\mathbf{MX}}$ (kg mol^{-1})	$\boldsymbol{C}_{\mathbf{MX}}^{\boldsymbol{\phi}}$ (kg^2 mol^{-2})	$\boldsymbol{\beta}_{\mathbf{MMic}}$ (kg mol^{-1})	$\boldsymbol{C}_{\mathbf{MMic}}^{\boldsymbol{\phi}}$ (kg^2 mol^{-2})	$\boldsymbol{T}$ (K)
**NaDec**	-0.9259	4.6700	1.1596	-0.6462	298.15
	1.7760		8.1080		

Table S10. The molar masses (*M*), liquid and solid densities (ρ_l and ρ_s) and surface tensions (σ) of water and sodium decanoate at 298.15 K (unless stated otherwise).

Compound	M (g mol^{-1})	ρ_l (kg m^{-3})	ρ_s (kg m^{-3})	σ (mN m^{-1})
**Water**	18.0153	997.0[a]		72.0[b]
**Sodium decanoate**	194.2470	1099.2[c]	1099.2[c]	22.5[d]

[a]Pátek et al. [58]

[b]IAPWS, *Revised Release on Surface Tension of Ordinary Water Substance*: International Association for the Properties of Water and Steam, 2014.

[c]Extended from the binary density of water–NaDec calculated via the method of Calderón and Prisle. [62]

[d]Surface tension of pure sub-cooled NaDec assumed equal to the surface tension for a NaDec solution calculated with Eq. 35 at the CMC (Eq. 43).

Table S11. Droplet diameters (d_c), supersaturations (SS_c) and surface tensions (σ_c) at the critical point of droplet activation, corresponding to Fig. S19, predicted with the different water activity models at 298.15 K for dry sodium decanoate particles across a range of diameters (D_p).

Parameter	d_c (nm)	SS_c (%)	σ_c (mN m^{-1})	d_c (nm)	SS_c (%)	σ_c (mN m^{-1})	d_c (nm)	SS_c (%)	σ_c (mN m^{-1})
**D_p (nm)**	Mono			MonoMic			CISA		
**50**	258	0.49	46.7	258	0.49	46.7	257	0.5	46.5
**100**	705	0.18	50.3	705	0.18	50.3	700	0.18	50.0
**150**	1265	0.1	52.9	1265	0.1	52.9	1256	0.1	52.7
**200**	1909	0.07	54.9	1909	0.07	54.9	1897	0.07	54.7
**D_p (nm)**	Clu			CluMic			CluMicNa		
**50**	243	0.51	44.5	243	0.51	44.5	243	0.51	44.5
**100**	642	0.2	47.3	642	0.2	47.3	642	0.2	47.3
**150**	1131	0.11	49.6	1131	0.11	49.6	1131	0.11	49.6
**200**	1687	0.08	51.5	1687	0.08	51.5	1687	0.08	51.5

References

1 R. L. Vold, J. S. Waugh, M. P. Klein and D. E. Phelps, *J. Chem. Phys.*, 1968, **48**, 3831–3832.

2 S. Meiboom and D. Gill, *Rev. Sci. Instrum.*, 1958, **29**, 688–691.

3 J. E. Tanner, *J. Chem. Phys.*, 1970, **52**, 2523–2526.

4 R. M. Cotts, M. J. R. Hoch, T. Sun and J. T. Markert, *J. Magn. Reson.*, 1989, **83**, 252–266.

5 O. Mankinen, V. V. Zhivonitko, A. Selent, S. Mailhiot, S. Komulainen, N. L. Prisle, S. Ahola and V.-V. Telkki, *Nat. Commun.*, 2020, **11**, 3251.

6 O. Söderman and P. Stilbs, *Prog. Nucl. Magn. Reson. Spectrosc.*, 1994, **26**, 445–482.

7 O. Söderman, P. Stilbs and W. S. Price, *Concepts Magn. Reson. A*, 2004, **23A**, 121–135.

8 J. Zhao and B. M. Fung, *Langmuir*, 1993, **9**, 1228–1231.

9 D. A. Case, H. Metin Aktulga, K. Belfon, D. S. Cerutti, G. A. Cisneros, V. Wilian, D. Cruzeiro, N. Forouzesh, T. J. Giese, A. W. Götz, H. Gohlke, S. Izadi, K. Kasavajhala, M. C. Kaymak, E. King, T. Kurtzman, T.-S. Lee, P. Li, J. Liu, T. Luchko, R. Luo, M. Manathunga, M. R. Machado, H. M. Nguyen, K. A. O'hearn, A. V Onufriev, F. Pan, S. Pantano, R. Qi, A. Rahnamoun, A. Risheh, S. Schott-Verdugo, A. Shajan, J. Swails, J. Wang, H. Wei, X. Wu, Y. Wu, S. Zhang, S. Zhao, Q. Zhu, T. E. Cheatham, D. R. Roe, A. Roitberg, C. Simmerling, D. M. York, M. C. Nagan and K. M. Merz, *Cite This: J. Chem. Inf. Model*, 2023, **63**, 6191.

10 Y. Wu, H. L. Tepper and G. A. Voth, *J. Chem. Phys.*, DOI:10.1063/1.2136877.

11 J. Liu, D. Li and X. Liu, *J. Chem. Phys.*, DOI:10.1063/1.4954990.

12 T. J. Giese and D. M. York, *J. Chem. Theory. Comput.*, 2016, **12**, 2611–2632.

13 R. C. Walker, M. F. Crowley and D. A. Case, *J. Comput. Chem.*, 2008, **29**, 1019–1031.

14 A. S. Christensen, T. Kubař, Q. Cui and M. Elstner, *Chem. Rev.*, 2016, **116**, 5301–5337.

15 C. J. Dickson, L. Rosso, R. M. Betz, R. C. Walker and I. R. Gould, *Soft Matter*, 2012, **8**, 9617.

16 J. Kowalewski and L. Mäler, *Nuclear Spin Relaxation in Liquids Theory, Experiments, and Applications Second Edition*, Taylor & Francis Group, New York, USA, 2006.

17 D. M. Brink and G. R. Satchler, *Angular Momentum*, Oxford University PressOxford, 1994.

18 K. Åman, E. Lindahl, O. Edholm, P. Håkansson and P.-O. Westlund, *Biophys. J.*, 2003, **84**, 102–115.

19 P. Håkansson, T. Boirin and J. Vaara, *Langmuir*, 2018, **34**, 3755–3766.

20 B. Halle and H. Wennerström, *J. Chem. Phys.*, 1981, **75**, 1928–1943.

21 H. Wennerstrom, G. Lindblom and B. Lindman, *Chem. Scr.*

22 K. Aidas, C. Angeli, K. L. Bak, V. Bakken, R. Bast, L. Boman, O. Christiansen, R. Cimiraglia, S. Coriani, P. Dahle, E. K. Dalskov, U. Ekström, T. Enevoldsen, J. J. Eriksen, P. Ettenhuber, B. Fernández, L. Ferrighi, H. Fliegl, L. Frediani, K. Hald, A. Halkier, C. Hättig, H. Heiberg, T. Helgaker, A. C. Hennum, H. Hettema, E. Hjertenæs, S. Høst, I. Høyvik, M. F. Iozzi, B. Jansík, H. J. Aa. Jensen, D. Jonsson, P. Jørgensen, J. Kauczor, S. Kirpekar, T. Kjærgaard, W. Klopper, S. Knecht, R. Kobayashi, H. Koch, J. Kongsted, A. Krapp, K. Kristensen, A. Ligabue, O. B. Lutnæs, J. I. Melo, K. V. Mikkelsen, R. H. Myhre, C. Neiss, C. B. Nielsen, P. Norman, J. Olsen, J. M. H. Olsen, A. Osted, M. J. Packer, F. Pawlowski, T. B. Pedersen, P. F. Provasi, S. Reine, Z. Rinkevicius, T. A. Ruden, K. Ruud, V. V. Rybkin, P. Sałek, C. C. M. Samson, A. S. de Merás, T. Saue, S. P. A. Sauer, B. Schimmelpfennig, K. Sneskov, A. H. Steindal, K. O. Sylvester-Hvid, P. R. Taylor, A. M. Teale, E. I. Tellgren, D. P. Tew, A. J. Thorvaldsen, L. Thøgersen, O. Vahtras, M. A. Watson, D. J. D. Wilson, M. Ziolkowski and H. Ågren, *WIREs Comput. Mol. Sci.*, 2014, **4**, 269–284.

23 P. J. W. Debye, *Polar Molecules*, Chemical Catalog Company, Incorporated, 1929.

24 C. C. Miller, *Proc. R. Soc. Lond. Ser. A-Contain. Pap. Math. Phys. Character*, 1924, **106**, 724–749.

25 The International Association for the Properties of Water and Steam, *Release on the IAPWS Formulation 2008 for the Viscosity of Ordinary Water Substance*, Berlin, 2008.

26 J. Kestin, M. Sokolov and W. A. Wakeham, *J. Phys. Chem. Ref. Data*, 1978, **7**, 941–948.

27 I. Teraoka, *Polymer Solutions*, Wiley, 2002.

28 J. S. Murday and R. M. Cotts, *J. Chem. Phys.*, 1970, **53**, 4724–4725.

29 M. Schönhoff, *Curr. Opin. Colloid Interface Sci.*, 2013, **18**, 201–213.

30 B. Halle, *J. Chem. Phys.*, 1991, **94**, 3150–3168.

31 J. Perlo, C. J. Meledandri, E. Anoardo and D. F. Brougham, *J. Phys. Chem. B*, 2011, **115**, 3444–3451.

32 P. Håkansson, M. A. Javed, S. Komulainen, L. Chen, D. Holden, T. Hasell, A. Cooper, P. Lantto and V.-V. Telkki, *Phys. Chem. Chem. Phys.*, 2019, **21**, 24373–24382.

33 U. von Toussaint, *Rev. Mod. Phys.*, 2011, **83**, 943–999.

34 I. Furó, *J. Mol. Liq.*, 2005, **117**, 117–137.

35 A. F. de Moura and L. C. G. Freitas, *Chem. Phys. Lett.*, 2005, **411**, 474–478.

36 B. Jönsson, H. Wennerström, P. G. Nilsson and P. Linse, *Colloid. Polym. Sci.*, 1986, **264**, 77–88.

37 J. N. Israelachvili and H. Wennerstroem, *J. Phys. Chem.*, 1992, **96**, 520–531.

38 J.-P. Douliez, A. Léonard and E. J. Dufourc, *J. Phys. Chem.*, 1996, **100**, 18450–18457.

39 N. L. Prisle, M. Dal Maso and H. Kokkola, *Atmos. Chem. Phys.*, 2011, **11**, 4073–4083.

40 P. Håkansson, *Phys. Chem. Chem. Phys.*, 2021, **23**, 2637–2648.

41 R. E. Kass and A. E. Raftery, *Bayes factors. J. Am. Stat. Assoc*, 1995, **90**, 773–795.

42 H. Köhler, *Trans. Faraday Soc.*, 1936, **32**, 1152–1161.

43 N. L. Prisle, T. Raatikainen, R. Sorjamaa, B. Svenningsson, A. Laaksonen and M. Bilde, *Tellus Ser. B-Chem. Phys. Meteorol.*, 2008, **60**, 416.

44 S. Vepsäläinen, S. M. Calderón and N. L. Prisle, *Atmos. Chem. Phys.*, 2023, **23**, 15149–15164.

45 N. L. Prisle, T. Raatikainen, A. Laaksonen and M. Bilde, *Atmos. Chem. Phys.*, 2010, **10**, 5663–5683.

46 H. A. Khwaja, *Atmos. Environ.*, 1995, **29**, 127–139.

47 M. Mochida, Y. Kitamori, K. Kawamura, Y. Nojiri and K. Suzuki, *J. Geophys. Res. Atmos.*, 2002, **107**, AAC 1-1-AAC 1-10.

48 M. Mochida, K. Kawamura, N. Umemoto, M. Kobayashi, S. Matsunaga, H.-J. Lim, B. J. Turpin, T. S. Bates and B. R. T. Simoneit, *J. Geophys. Res. Atmos.*, DOI:https://doi.org/10.1029/2002JD003249.

49 J. Malila and N. L. Prisle, *J. Adv. Model Earth Syst.*, 2018, **10**, 3233–3251.

50 B. R. Bzdek, J. P. Reid, J. Malila and N. L. Prisle, *Proc. Natl. Acad. Sci.*, 2020, **117**, 8335–8343.

51 S. Vepsalainen, S. M. Calderon, J. Malila and N. L. Prisle, *Atmos. Chem. Phys.*, 2022, **22**, 2669–2687.

52 J. J. Lin, T. B. Kristensen, S. M. Calderón, J. Malila and N. L. Prisle, *Environ. Sci. Process. Impacts*, 2020, **22**, 271–284.

53 J. J. Lin, J. Malila and N. L. Prisle, *Environ. Sci. Process. Impacts*, 2018, **20**, 1611–1629.

54 P. Håkansson, P. M. Delgado, A. Selent, R. Ghanghas, I. Ainasoja, S. Komulainen, J. Mareš, P. Lantto, N. L. Prisle and V.-V. Telkki, *Soft Matter*, 2026, **22**, 2800–2808.

55 S. M. Calderón, J. Malila and N. L. Prisle, *J. Atmos. Chem.*, 2020, **77**, 141–168.

56 E. Vikingstad, A. Skauge and H. Høiland, *J. Colloid Interface Sci.*, 1978, **66**, 240–246.

57 K. S. Pitzer and C. R. C. Press, *Activity coefficients in electrolyte solutions*, CRC press Boca Raton, FL, 1991, vol. 2.

58 J. Pátek, J. Hrubý, J. Klomfar, M. Součková and A. H. Harvey, *J. Phys. Chem. Ref. Data*, 2009, **38**, 21–29.

59 D. P. Fernández, A. R. H. Goodwin, E. W. Lemmon, J. M. H. Levelt Sengers and R. C. Williams, *J. Phys. Chem. Ref. Data*, 1997, **26**, 1125–1166.

60 R. M. Smith and A. E. Martell, *Critical stability constants: second supplement*, Springer, 1989, vol. 6.

61 N. L. Prisle, J. Vanhanen, T. Raatikainen, J. J. Lin and A.-P. Hyvärinen, .

62 S. M. Calderón and N. L. Prisle, *J. Atmos. Chem.*, 2021, **78**, 99–123.

63 E. Blanco, A. González-Pérez, J. M. Ruso, R. Pedrido, G. Prieto and F. Sarmiento, *J. Colloid Interface Sci.*, 2005, **288**, 247–260.

64 R. De Lisi, G. Perron and J. E. Desnoyers, *Can. J. Chem.*, 1980, **58**, 959–969.

65 N. L. Prisle, *Atmos. Chem. Phys.*, 2021, **21**, 16387–16411.

66 M. D. Petters and S. M. Kreidenweis, *Atmos. Chem. Phys.*, 2007, **7**, 1961–1971.

67 N. L. Prisle, J. J. Lin, S. Purdue, H. Lin, J. C. Meredith and A. Nenes, *Atmos. Chem. Phys.*, 2019, **19**, 4741–4761.

68 A. Jaatinen, S. Romakkaniemi, T. Anttila, A.-P. Hyvärinen, L.-Q. Hao, A. Kortelainen, P. Miettinen, S. Mikkonen, J. N. Smith and A. Virtanen, *Boreal environment research*.

69 M. D. Petters and S. M. Kreidenweis, *Atmos. Chem. Phys.*, 2008, **8**, 6273–6279.

70 C. M. Carrico, M. D. Petters, S. M. Kreidenweis, J. L. Collett, G. Engling and W. C. Malm, *J. Geophys. Res. Atmos.*, DOI:10.1029/2007JD009274.

71 P. Massoli, A. T. Lambe, A. T. Ahern, L. R. Williams, M. Ehn, J. Mikkilä, M. R. Canagaratna, W. H. Brune, T. B. Onasch, J. T. Jayne, T. Petäjä, M. Kulmala, A. Laaksonen, C. E. Kolb, P. Davidovits and D. R. Worsnop, *Geophys. Res. Lett.*, DOI:10.1029/2010GL045258.

72 D. O. Topping and G. McFiggans, *Atmos. Chem. Phys.*, 2012, **12**, 3253–3260.

73 I. Riipinen, N. Rastak and S. N. Pandis, *Atmos. Chem. Phys.*, 2015, **15**, 6305–6322.

74 A. M. K. Hansen, J. Hong, T. Raatikainen, K. Kristensen, A. Ylisirniö, A. Virtanen, T. Petäjä, M. Glasius and N. L. Prisle, *Atmos. Chem. Phys.*, 2015, **15**, 14071–14089.

75 T. B. Kristensen, N. L. Prisle and M. Bilde, *Atmos. Res.*, 2014, **137**, 167–175.

76 M. C. Facchini, M. Mircea, S. Fuzzi and R. J. Charlson, *Nature*, 1999, **401**, 257–259.

77 R. J. Charlson, J. E. Lovelock, M. O. Andreae and S. G. Warren, *Nature*, 1987, **326**, 655–661.

78 R. Wood, *Mon. Weather Rev.*, 2012, **140**, 2373–2423.
